# Spiro-helicenoid Carbofluoresceins: CPL-Active Emitters for Bioimaging

**DOI:** 10.1021/acs.orglett.6c02744

**Published:** 2026-08-12

**Authors:** Laura Erviti-Marticorena, Silvia Simón-Fuente, Sandra Míguez-Lago, José Manuel Paredes, María Ribagorda, María Dolores Girón-González, Alba Millán, Juan M. Cuerva, Delia Miguel

**Affiliations:** † Department of Organic Chemistry, Faculty of Sciences, 16741 University of Granada, 18071 Granada, Spain; ‡ Department of Organic Chemistry, Faculty of Sciences, 16722 Universidad Autónoma de Madrid, 28049 Madrid, Spain; § Unidad de Excelencia de Química Aplicada a Biomedicina y Medioambiente, University of Granada, 18071 Granada, Spain; ∥ Nanoscopy-UGR Laboratory, Department of Physical Chemistry, Faculty of Pharmacy, Universidad de Granada, 18071 Granada, Spain; ⊥ Institute for Advanced Research in Chemical Sciences (IAdChem), Universidad Autónoma de Madrid, 28049 Madrid, Spain; # Department of Biochemistry and Molecular Biology II, Faculty of Pharmacy, University of Granada, 18071 Granada, Spain

## Abstract

Chiral fluorescent probes based on small organic molecules present a promising platform for advanced bioimaging applications. In this work, we have developed a helically chiral spiro-shaped carbofluorescein with red emission (λ_em_ ≤ 613 nm). Configurationally stable enantiomers were separated, and electronic circular dichroism (ECD) and circularly polarized luminescence (CPL) evaluated. Preliminary results showed cellular permeability and intracellular emission.

## Graphical abstract

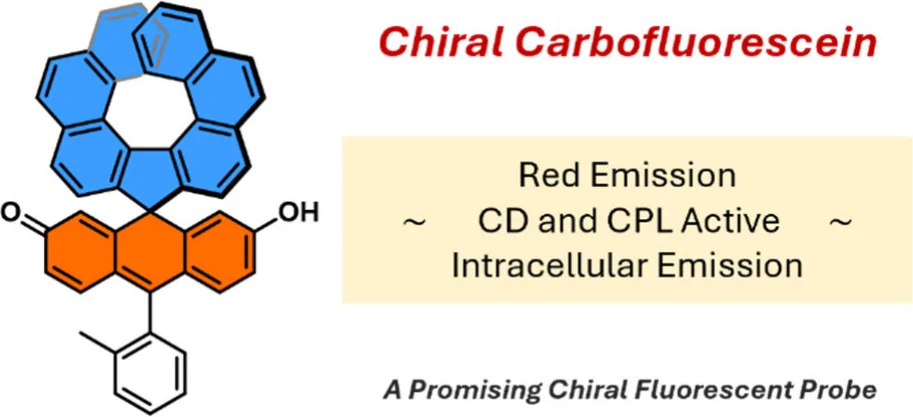

Fluorescent dyes are indispensable tools in biology for tracking and imaging molecules in their native contexts.^1,2^ Among the vast number of fluorophores reported in the literature, sensors emitting in the red or near-infrared (NIR) regions offer critical advantages for bioimaging over traditional green-emitting probes.^3,4^ Specifically, red-shifted emitters present several benefits related to reduced phototoxicity and lower energy, deeper tissue penetration, and a higher signal-to-noise ratio, as autofluorescence typically increases in the green spectrum. Some of the most widely used scaffolds are cyanines,^5^ silicon-rhodamines,^6,7^ and derivatives of prototypical fluorescein, in which the emission is red-shifted upon replacement of the oxygen atom of the xanthene core with silicon^8–10^ or carbon atoms.^11^ In the latter case, the family of carbofluoresceins shifts the optical response directly toward the near-IR region, precisely matching the therapeutic window of light (approximately 650–900 nm). Despite this outstanding property, this skeleton remains relatively underexplored in the literature, with key examples including the Virginia orange derivatives synthesized by Lavis and co-workers (Figure 1)^11–14^ and the sulfonated carbofluorescein reported by Miller for voltage imaging.^15^

**Figure 1:**
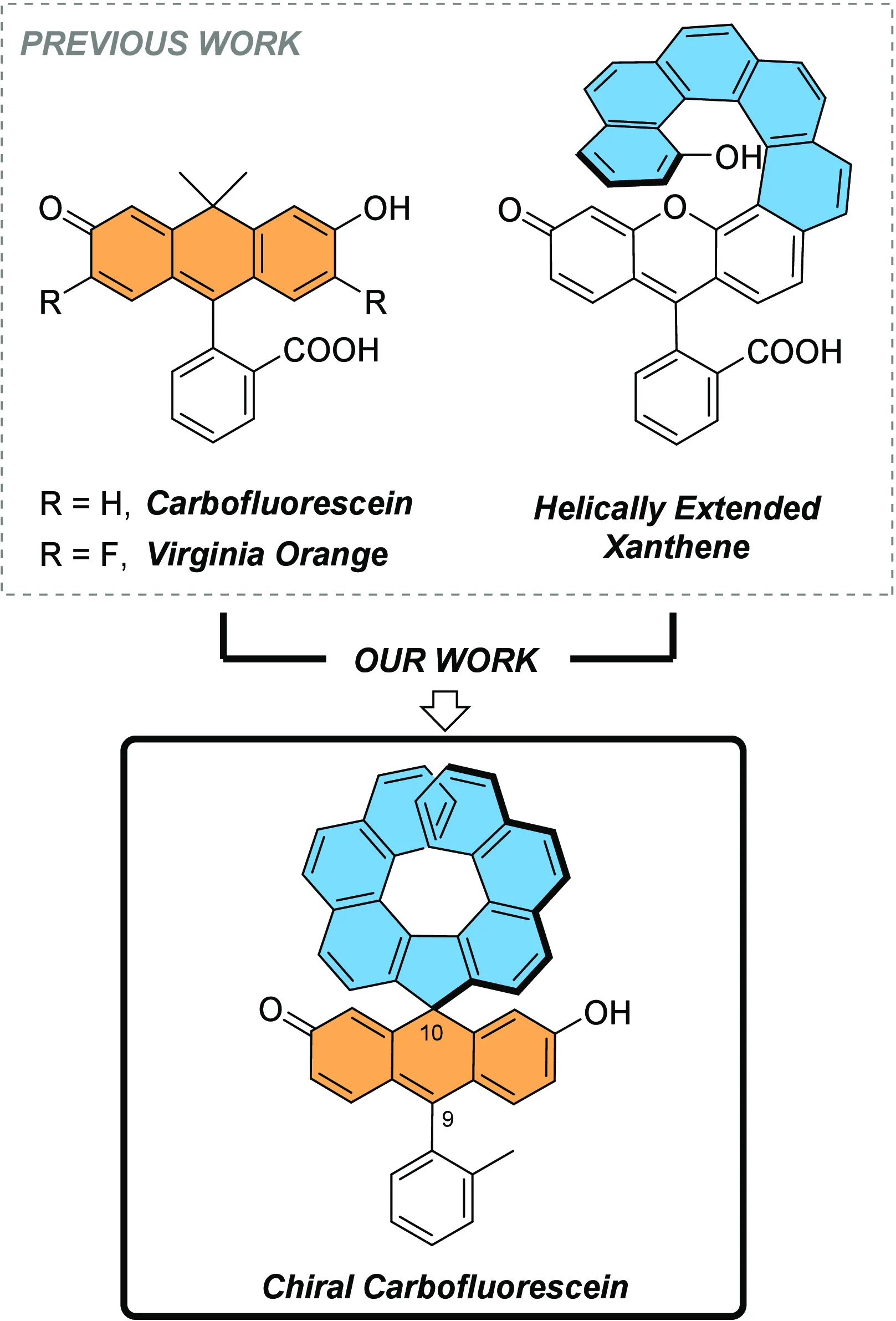
Fluorescein isologues described in the literature and the chiral carbofluorescein developed in this work.

Furthermore, all of these scaffolds that possess excellent photophysical properties for biological applications are inherently achiral. Considering the three-dimensional chiral space of biological environments, chiral fluorescent dyes offer vast untapped potential. Consequently, there is growing interest in developing chiral probes for biosensing purposes,^16^ such as enantiomer discrimination^17–19^ or enantioselective chiral recognition.^20^ In addition, chiral emitters exhibit circularly polarized luminescence (CPL),^21,22^ which refers to the differential emission of left- and right-handed circularly polarized light, which provides an additional degree of freedom in the context of cell imaging.^23^ State-of-the-art microscopy techniques, such as a CPL-laser scanning confocal microscope (LSCM), utilize this phenomenon to distinguish or monitor chiral systems in live cell bioimaging studies.^24,25^ Therefore, the design of new CPL-active and stimulus-responsive dyes or probes, such as carbofluoresceins, is highly desirable. However, the incorporation of chiral entities into the fluorescein family has not been explored in depth, with examples limited to a helically extended xanthene core (Figure 1).^26^ In this context, merging the red-shifted carbofluorescein scaffold with molecular asymmetry introduces a promising new dimension to fluorescein-based probes, paving the way for high-contrast and enantioselective chiroptical sensing in complex biological environments.

On the basis of these antecedents, we have designed a chiral carbofluorescein by attaching a chiral scaffold to the carbon at position 10 (Figure 1). Since helicenes and helicenoids stand out as chiral motifs due to their excellent chiroptical responses, we selected [5]- and [7]-helicenoid as sources of chirality.^27,28^ Moreover, to hamper a potential deactivation of the fluorescence, we introduced an *ortho*-substituted aryl group at C-9. This modular design would allow a fine-tuning of the properties by careful selection of the central substituents of the carbofluorescein core.

The synthesis of the target dyes is depicted in Scheme 1. The spirocyclic scaffold was assembled from 2-bromodiarylmethane **2** ^29^ through an initial nucleophilic addition to helical ketones *rac*-**1a** ([7]-helicenoid) or *rac*-**1b** ([5]-helicenoid),^30^ mediated by a bromolithium exchange and followed by acid-mediated Friedel–Crafts cyclization to afford compound *rac*-**3a** or *rac*-**3b**, respectively. Then, the methoxy groups (-OMe) were strategically converted into methoxymethyl ethers (-OMOM), and a subsequent benzylic oxidation of these compounds gave ketone *rac*-**5a** or *rac*-**5b**, respectively, in good yield. Final helical carbofluorescein derivatives *rac-* **7H-CF** and *rac-* **5H-CF** were obtained as purple solids by nucleophilic addition using *o*-tolyllithium, followed by acid-mediated MOM deprotection and concomitant double-bond formation (see the Supporting Information for further details).

**Scheme 1:**
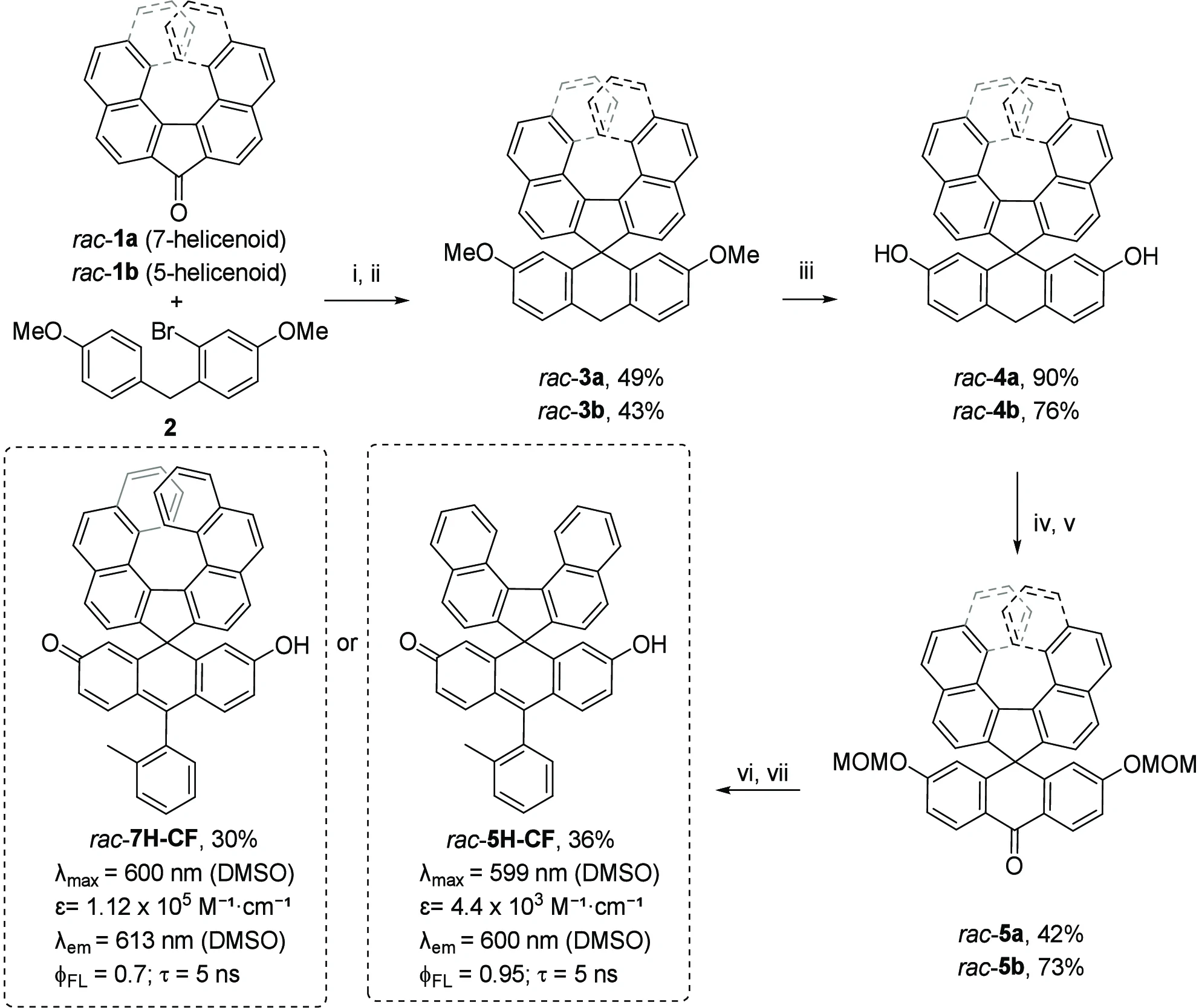
Synthesis of Carbofluoresceins *rac-* **7H-CF** and *rac-* **5H-CF** ^a^

a Reagents and conditions: (i) *n*-BuLi, THF, −78 °C to rt, 3 h; (ii) MeSO_3_H, DCM, rt, 3 h; (iii) BBr_3_, DCM, 0 °C, 3 h; (iv) NaH, MOM-Cl, DMF, 0 °C to rt, 16 h; (v) KMnO_4_, MeCN, 60 °C, 16 h; (vi) *n*-BuLi, 2-bromotoluene, THF, −78 °C to rt, 3 h; (vii) aqueous HCl, MeOH, rt, 16 h.

Next, we investigated their photophysical properties. We initially determined the absorption and emission spectra of *rac*-**5H-CF** in EtOH and DMSO solutions, observing quite similar profiles in both solvents (see Figure S30). In EtOH, the maximum appeared as a narrow band at 579 nm (ε_579_ = 3.99 × 10^3^ M^–1^ cm^–1^) with a shoulder at 540 nm, with additional contributions at 470, 371, and 354 nm. In DMSO, a similar profile was observed, albeit with a bathochromic shift of approximately 20 nm (ε_597_ = 7.4 × 10^3^ M^–1^ cm^–1^) and the absence of the band around 440 nm. Regarding the emission spectra, *rac*-**5H-CF** presents a band spanning up to ∼700 nm with emission maxima at 597 nm in EtOH and 616 nm in DMSO. As expected, *rac*-**7H-CF** showed almost identical optical properties but with ε values one order of magnitude higher due to the larger π-extension of the helicenoid unit. We initially measured the absorption (Figure 2a, solid line) and emission (Figure 2a, dashed line) spectra of *rac*-**7H-CF** in EtOH. The absorption profile exhibits an intense, narrow band (λ_max_ = 580 nm; ε = 3.69 × 10^4^ M^–1^ cm^–1^) and a less intense band spanning 420–520 nm. Regarding emission, excitation at 530 nm yielded a maximum wavelength of 595 nm. In the case of *rac*-**5H-CF**, similarly shaped but red-shifted profiles were obtained in DMSO (λ_max,abs_ = 600 nm; ε = 1.12 × 10^5^ M^–1^ cm^–1^; λ_max,em_ = 613 nm). This orange emission is slightly superior to that of sila-fluorescein TokyoMagenta.^10^ In addition, considering the presence of the helicene moiety, samples were also excited at 320 nm (Figure S32), showing a shoulder between 400 and 420 nm that is more prominent for the case of **7H-CF**. This emission is possible due to the minimal overlap in both the absorption and emission spectra of the two subunits of the molecule: the helicenoid and the carbofluorescein core.

**Figure 2:**
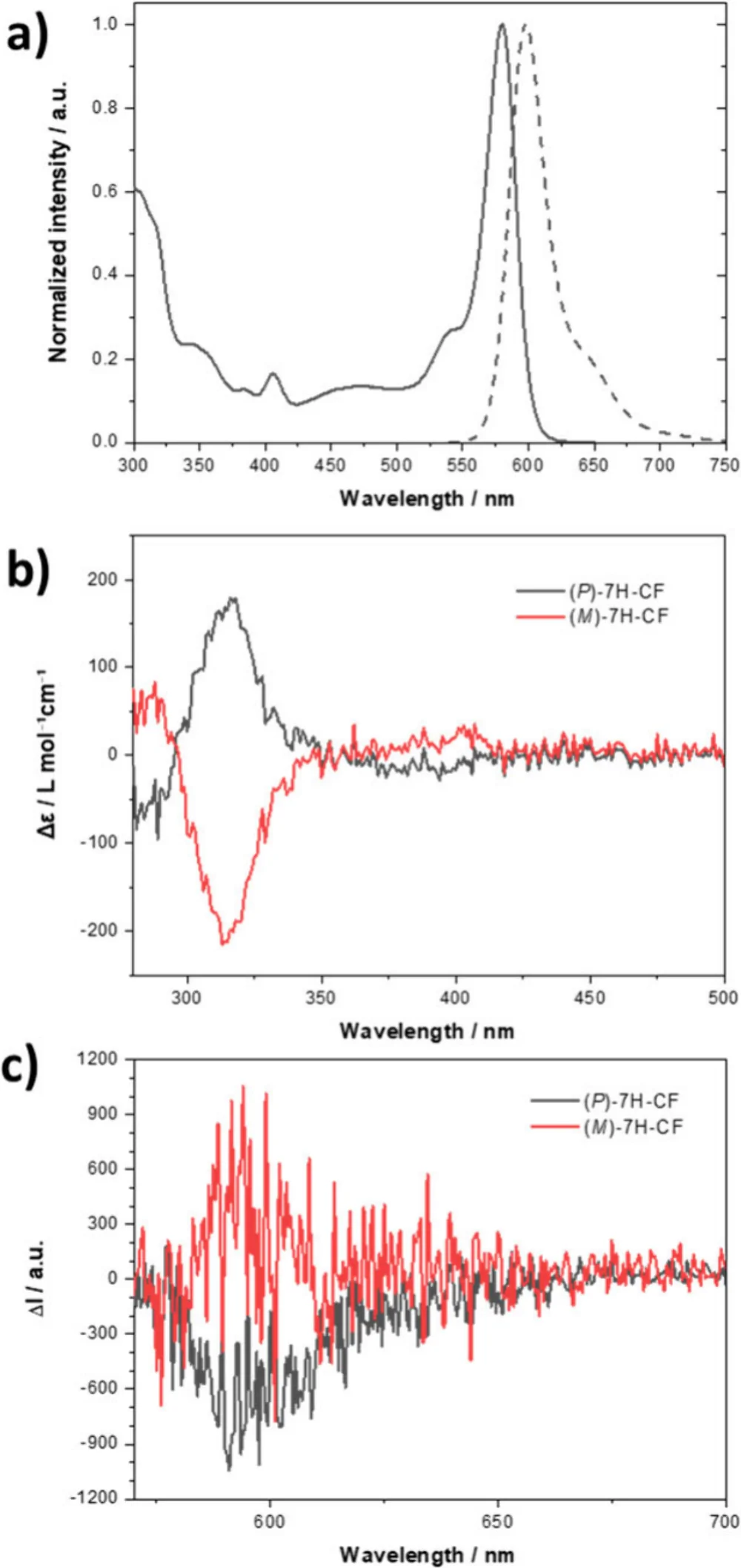
(a) Normalized absorption (solid line) and emission (dashed line; λ_exc_ = 530 nm) spectra of *rac*-**7H-CF** in EtOH. (b) ECD and (c) CPL spectra of both enantiomers of **7H-CF** in EtOH.

We completed the photophysical study by evaluating the efficiency of the emission process. Both carbofluoresceins showed good quantum yield (QY) values in EtOH (74% for *rac*-**7H-CF** and 65% for *rac*-**5H-CF**) and DMSO (70% for *rac*-**7H-CF**), with a remarkable value of 95% for *rac*-**5H-CF** in DMSO. On the other hand, fluorescence lifetimes fit to a monoexponential function, with values of around 4 ns for both compounds in EtOH and 5 ns in DMSO for *rac*-**7H-CF** (see Tables S1 and S2). Finally, we evaluated the effect of pH on absorption and photoluminescence due to its importance for biological application. For *rac*-**7H-CF**, a decrease in the intensity of the absorption band around 470 nm is observed as the pH increases and the reverse effect is observed for the band at 580 nm (Figure S38). This suggests that the former band corresponds to the neutral species and the latter to the anionic form. Consequently, the fluorescence emission intensity around 590 nm (λ_exc_ = 530 nm) increases with pH. The same trend was observed for *rac*-**5H-CF**. From these variations, we determine a p*K*_a_ value of 8.25 and a p*K*_a_* value of 9.22 for **7H-CF**, whereas *rac*-**5H-CF** presented slightly lower values (p*K*_a_ = 8.16, and p*K*_a_* = 9.10) (see Figures S39–S42).

Next, we investigated the chiroptical properties. We focused only on **7H-CF** due to the relatively low enantiomerization barrier found in [5]-helicenoids of this type.^31^ To this end, the (*P*)-**7H-CF** and (*M*)-**7H-CF** enantiomers were separated by high-performance liquid chromatography (HPLC) using a chiral stationary phase and a recirculating eluent system (*n*-hexane/EtOAc (3:7, v/v)). We first measured the electronic circular dichroism (ECD) in EtOH, the first eluted peak being a positive band at 317 nm. As expected, the two enantiopure samples displayed perfect mirror-image ECD spectra (Figure 2b). The spectra of (*P*)-**7H-CF** showed the previously mentioned prominent positive Cotton effect (Δε = 176 M^–1^ cm^–1^; λ = 317 nm) with opposite sign bands at 280 nm (Δε = −70 M^–1^ cm^–1^) and 400 nm (Δε = −17 M^–1^ cm^–1^). The absorption dissymmetry factor (*g*_abs_) reaches 8 × 10^–4^ (λ = 318 nm). We then turned our attention to the excited-state chirality by measuring the CPL in EtOH using a 490 nm LED excitation source, achieving a maximum CPL at 597 nm. Once again, mirror-image CPL signals were recorded (Figure 2c). The resulting luminescence dissymmetry factor (*g*_lum_) was found to be on the order of 5 × 10^–4^.

Although the sign of the Cotton effect at 317 nm suggests a *P* configuration for the first eluted peak and an *M* configuration for the second eluted fraction, a rigorous absolute configuration assignation was performed using TD-DFT calculations at the CAM-B3LYP/def2SVP level of theory (PCM = EtOH). For the neutral and anionic species, the predicted ECD signal at 317 nm is negative for the *M* enantiomer (Figure S53), confirming the absolute configuration of the second eluted peak. Interestingly, these calculations also elucidate the pH-dependent behavior of the fluorescence, the oscillator strength (*f*) of the neutral form (*f* = 0.6132) being weaker than that of the anionic species (*f* = 0.7274); the feature becomes even more noticeable in the calculated emission (*f* = 0.0004 for the neutral species vs *f* = 0.00174 for the anionic one), accounting for a 4-fold increase in *f*. Furthermore, the experimental dissymmetry factor aligns well with theoretical TD-DFT predictions (*g*_abs_ of ca. 10^–4^ for the less energetic transition). Regarding configurational stability, theoretical calculations predict a racemization energy barrier of 35.0 kcal mol^–1^ (M06/TZVP; PCM = DMSO) for **7H-CF**, in the range of that of [6]-helicene (36.2 kcal mol^–1^). Experimentally, this stability was confirmed when a sample of enantiopure **7H-CF** was heated at 100 °C in DMSO for 1 h without showing almost any change in its ECD spectrum. However, exposure to a higher temperature (150 °C) unfortunately led to sample decomposition.

Finally, we analyzed the behavior of enantiopure fluorophores (*M*)- and (*P*)-**7H-CF** in cell-based assays as a preliminary evaluation of their potential biological application. The emission of the fluorophore was collected in two independent channels: the blue one (between 400 and 450 nm, with λ_exc_ = 375 nm (Figure 3, top)) corresponding to the helicene core and the red one (between 580 and 630 nm, with λ_exc_ = 530 nm (Figure 3, bottom)) attributed to the carbofluorescein moiety. Both enantiomers showed cellular permeability in HeLa cells after incubation at a concentration of 2 μM for 3 h in the HKBR buffer solution (Figure S50) with no significant differences between them. Although some signal can be detected in the blue region, the most intense emission appears in the red channel, as would be expected from the fluorescence spectra obtained in solution.

**Figure 3:**
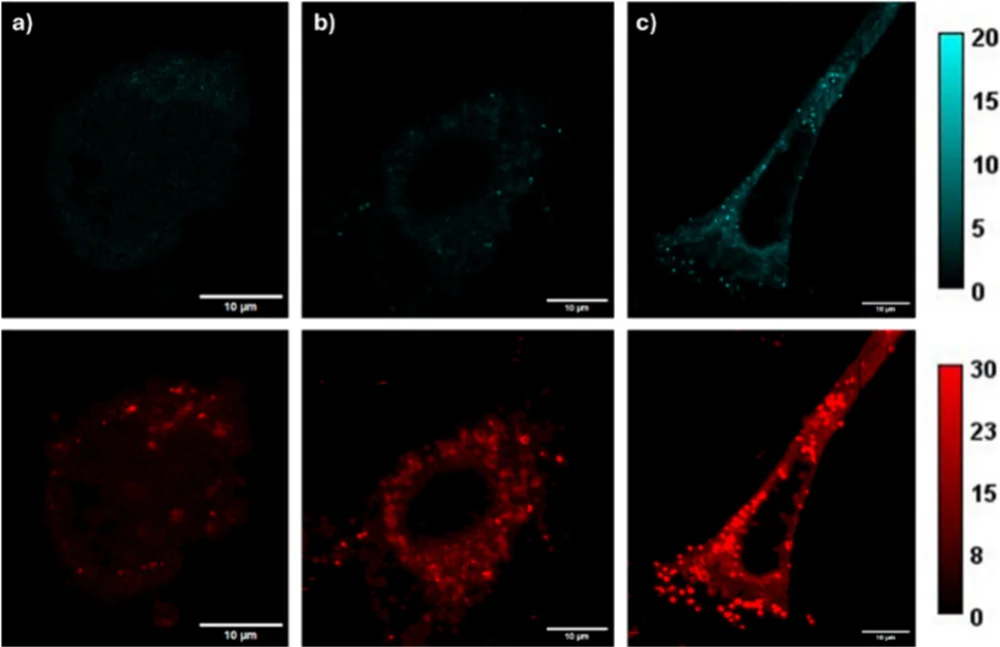
Live cell imaging of **7H-CF** recorded in the ranges of 400–450 (top) and 580–630 nm (bottom) of (a) cells without the compound and cells incubated with (b) (*P*)-**7H-CF** and (c) (*M*)-**7H-CF**.

In summary, we describe **7H-CF**, a novel chiral fluorescent probe that combines a carbofluorescein scaffold with a helicenoid unit to operate in the red wavelength region with high quantum yields. The enantiopure (*M*)- and (*P*)-**7H-CF** stereoisomers are configurationally stable, featuring a remarkably high calculated racemization energy barrier of 35.0 kcal mol^–1^. This structural stability enabled the comprehensive evaluation of their chiroptical properties, confirming that this spiro-helicenoid carbofluorescein is both ECD and CPL active. In addition, preliminary in vitro investigations demonstrated excellent cell permeability of both enantiomers in HeLa cells, along with intense intracellular fluorescence in the red channel.^32^ Consequently, **7H-CF** holds great promise as a probe for advanced bioimaging applications.

## Supplementary Material



## Data Availability

The data underlying this study are available in the published article and its Supporting Information.

## Notes

Synthetic procedures and characterization of compounds, copies of NMR spectra, additional photophysical studies and graphs, and details of theoretical calculation (PDF)
