# Supplementary material for: Spiro-helicenoid Carbofluoresceins: CPL-Active Emitters for Bioimaging
Source: Org Lett. 2026 Aug 12;28(34):10703–7. doi: 10.1021/acs.orglett.6c02744 (PMC13532169; doi:10.1021/acs.orglett.6c02744)
Supplement: Supplementary file 1 [file ol6c02744_si_001.pdf]

SUPPORTING INFORMATION

Spiro-helicenoid Carbofluoresceins: CPL-Active Emitters for Bioimaging

Laura Erviti-Marticorena,[a] Silvia Simón-Fuente,[b] Sandra Míguez-Lago,[a] José Manuel Paredes,[c,d] María Ribagorda,[b,e] María Dolores Girón-González,[c,f] Alba Millán,*[a,c] Juan M. Cuerva,*[a,c] Delia Miguel*[c,d]

[a] Department of Organic Chemistry, Faculty of Sciences, University of Granada, 18071 Granada, Spain.

[b] Department of Organic Chemistry, Faculty of Sciences, Universidad Autónoma de Madrid, 28049 Madrid, Spain

[c] Unidad de Excelencia de Química Aplicada a Biomedicina y Medioambiente, 18071, University of Granada, Granada, Spain.

[d] Nanoscopy-UGR Laboratory, Departamento of Physical Chemistry, Faculty of Pharmacy, Universidad de Granada, 18071 Granada, Spain.

[e] Institute for Advanced Research in Chemical Sciences (IAdChem), Universidad Autónoma de Madrid, 28049, Spain

[f] Department of Biochemistry and Molecular Biology II, Faculty of Pharmacy, University of Granada, Granada, 18071, Spain.

Table of content

1. CHEMICAL SYNTHESIS........ S3
   1.1. General Information........ S3
   1.2. Experimental Details and Characterization of Compounds........ S4
   1.3. ^{1}H- and ^{13}C-NMR Spectra and HRMS spectra of **5H-CF** and **7H-CF**........ S12
2. PHOTOPHYSICAL STUDIES........ S29
   2.1. General Information........ S29
   2.2. Absorbance and Emission in EtOH and DMSO........ S29
   2.3. Determination of Molar Extinction Coefficient (ε)........ S30
   2.4. Quantum Yield and Lifetime........ S32
   2.5. Effect of pH S34
      2.5.1. Absorbance and Fluorescence........ S34
      2.5.2. Determination of pK_a........ S35
3. CHIROPTICAL STUDIES........ S37
   3.1. General Information........ S37
   3.2. HPLC Separation of Enantiomers........ S37
   3.3. Electronic Circular Dichroism........ S39
   3.4. Dissymmetry Factors........ S39
   3.5. Thermal stability of (*P*)-**7H-CF**........ S41
4. LIVE-CELL IMAGING........ S42
5. THEORETICAL CALCULATIONS........ S45
6. REFERENCES........ S58

1. CHEMICAL SYNTHESIS

1.1. General Information

Compounds *rac*-**1a**, *rac*-**1b** and **2** were prepared according to literature procedures.[S1,S2] Otherwise stated, all reagents and solvents used in the procedures were obtained from commercial suppliers and used as received. Anhydrous THF was distilled over Na/benzophenone. Monitoring of the reaction and purification processes was performed using EMD Millipore thin-layer chromatography (TLC) on silica gel 60 F254, observed under ultraviolet light (254 nm) or using general-purpose developers such as phosphomolybdic acid (5% ethanol solution). All compounds were generally purified by flash column chromatography using a Merck 60 silica gel (particle size 0.040–0.063 mm, ASTM 230–400 mesh) as the stationary phase and a mixture of solvents as the mobile phase. The final products were ultimately purified by EMD Millipore silica gel 60 F254 preparative layer chromatography (PLC). All compounds were identified and characterized by high resolution mass spectrometry (HRMS) and ^{1}H- and ^{13}C-NMR. The following instruments were used: a WATERS XEVO TQ-XS Triple Quadrupole mass spectrometer and a BRUKER Avance NEO high-definition NMR spectrometer with frequencies of 400 MHz, 500 MHz, or 600 MHz. Deuterated chloroform and methanol were used as solvents, depending on the solubility of the compounds. NMR spectra were processed using MestReNova v.14 software. Chemical shifts were expressed in ppm and referenced to the residual solvent peak. Coupling constants (*J*) were expressed in hertz (Hz). Multiplicities were abbreviated as follows: s = singlet, d = doublet, t = triplet, m = multiplet, dd = double doublet, td = triple doublet, ddd = double double doublet, dt = double triplet. Proton assignment was performed using 2D NMR experiments (HSQC, HMBC, TOCSY, TOCSY-HSQC). Carbon assignment was performed using DEPT techniques.

1.2. Experimental Details and Characterization of Compounds

The synthesis of *rac*-**5H-CF** and *rac*-**7H-CF** required prior preparation of the corresponding fluorenones derivatives *rac*-**1a** and *rac*-**1b**[S1] and the brominated derivative **2**[S2] and according to previous literature reports. As outlined in Scheme S1, the synthetic route involved an initial nucleophilic addition of **2** to the carbonyl group of *rac*-**1a**/*rac*-**1b**, followed by intramolecular Friedel-Crafts reaction. Then a protecting group exchange and an oxidation step gave *rac*-**5a**/*rac*-**5b**. Then, a second nucleophilic addition to the carbonyl group was carried out using the corresponding aryl-lithium, followed by deprotection of phenols and final quinone formation, affording the carbofluorescein derivatives *rac*-**5H-CF** and *rac*-**7H-CF**.

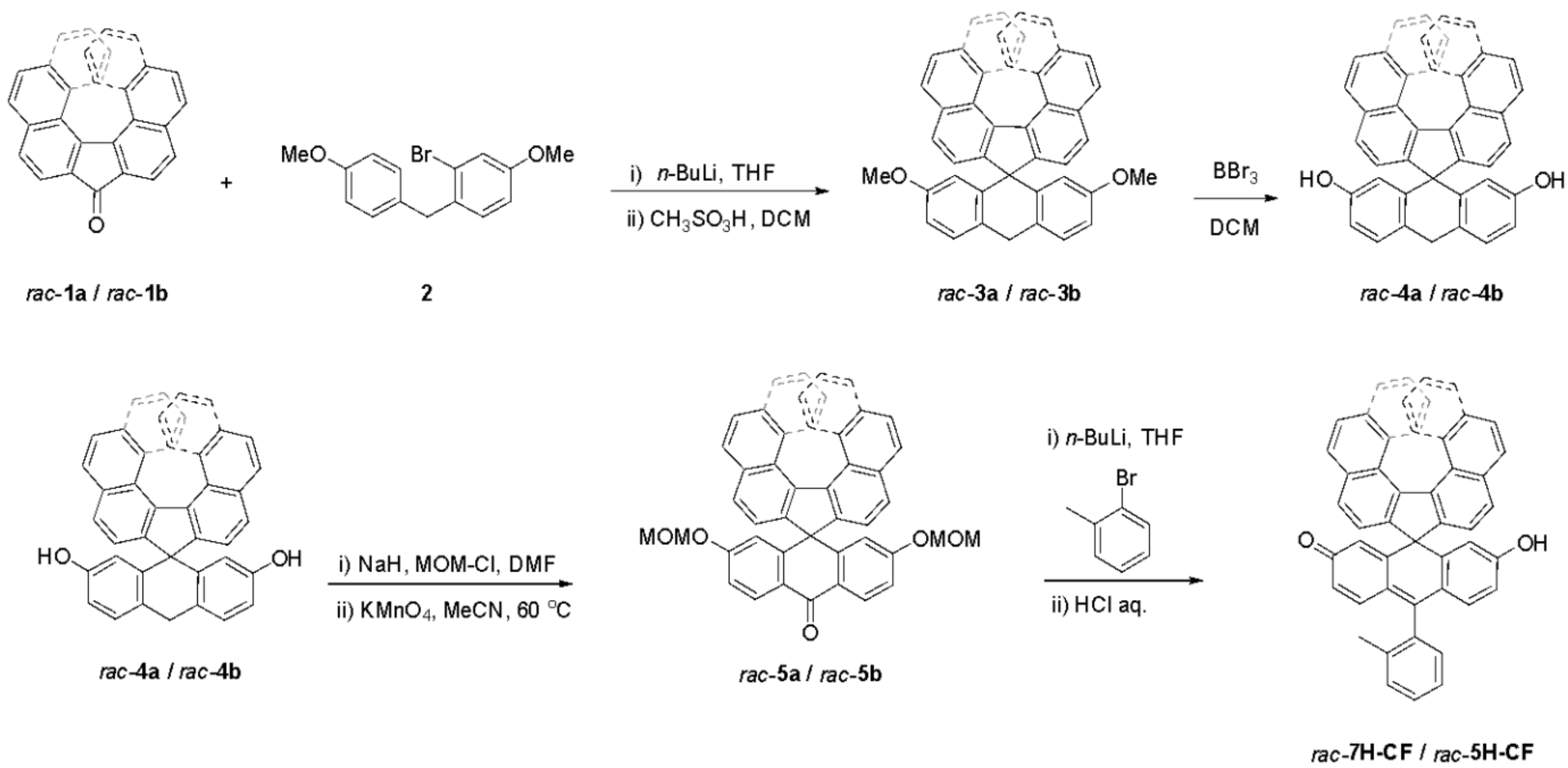

Scheme S1. General synthetic route for carbofluoresceines *rac*-**7H-CF** and *rac*-**5H-CF**.

Compound *rac*-3a

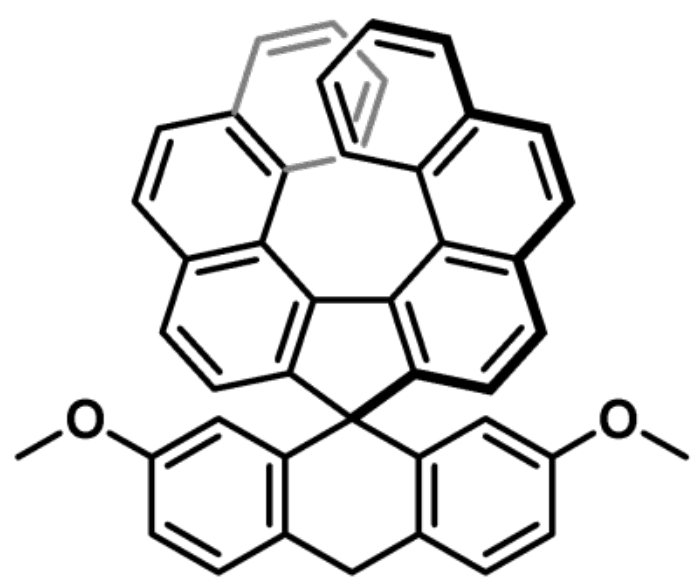

To a solution of the corresponding bromo derivative **2**[S2] (300 mg, 0.97 mmol, 4 equiv.) in anhydrous THF (4 mL) and under an argon atmosphere, *n*-BuLi 2.5 M in *n*-hexane (0.34 mL, 0.85 mmol, 3.5 equiv.) was added at -78 °C. The mixture was stirred at this temperature for 45 min, and it changed color to orange. Then, the lithiated derivative was added dropwise to a solution

of the fluorenone *rac*-**1a**[S1] (90 mg, 0.24 mmol, 1 equiv.) dissolved in anhydrous THF (1 mL) and under an argon atmosphere. The resulting solution was stirred at room temperature for 3 h and it changed color to brown. The reaction was quenched with a few drops of H_2O at 0 °C, and THF was removed. Then, EtOAc was added, and the organic phase washed with brine. The organic phase was dried over anhydrous Na_2SO_4, filtered, and the solvent was concentrated under reduced pressure.

Subsequently, the residue obtained was dissolved in anhydrous CH_2Cl_2 (2 mL) under an argon atmosphere. Methanesulfonic acid (0.10 mL, 0.25 mmol, 1 equiv.) was added and the reaction changed color to dark brown. The mixture was stirred at room temperature overnight. Then, the reaction was neutralized with a saturated $NaHCO_3$ aqueous solution and the aqueous layer extracted with CH_2Cl_2. The organic phase was dried over anhydrous Na_2SO_4, filtered, and the solvent was concentrated under reduced pressure. The product was purified by flash column chromatography on silica gel using *n*-hexane/EtOAc 9:1 as eluent, affording compound *rac*-**3a** (62 mg, 0.10 mmol, 49%) as a green-grey solid.

^{1}H NMR (400 MHz, $CDCl_3$) δ 7.77 – 7.72 (m, 8H), 7.64 (d, *J* = 6.6 Hz, 2H), 7.38 (d, *J* = 8.5 Hz, 2H), 7.34 (d, *J* = 7.9 Hz, 2H), 7.13 (ddd, *J* = 8.0, 6.9, 1.2 Hz, 2H), 6.78 (dd, *J* = 8.5, 2.7 Hz, 2H), 6.34 (ddd, *J* = 8.4, 7.0, 1.4 Hz, 2H), 6.13 (d, *J* = 2.6 Hz, 2H), 4.52 (s, 2H), 3.36 (s, 6H). **^{13}C NMR** (101 MHz, $CDCl_3$) δ 158.1 (C), 156.3 (C), 139.0 (C), 137.1 (C), 132.4 (C), 131.7 (C), 131.1 (C), 129.6 (CH), 129.4 (CH), 127.9 (C), 127.6 (CH), 127.4 (C), 126.9 (CH), 126.8 (CH), 126.6 (CH), 126.5 (CH), 123.4 (CH), 123.1 (CH), 113.0 (CH), 112.7 (CH), 60.3 (C), 55.0 (CH_3), 33.4 (CH_2). Note: Oxidation at the benzylic position occurs spontaneously under ambient atmospheric conditions, resulting in trace amounts of the corresponding ketone (minor impurities in the NMR spectra shown). **HRMS (ESI)** $C_{44}H_{30}O_2Na$ $[M+Na]^+$ calcd: 613.2143; found: 613.2159.

Compound *rac*-3b

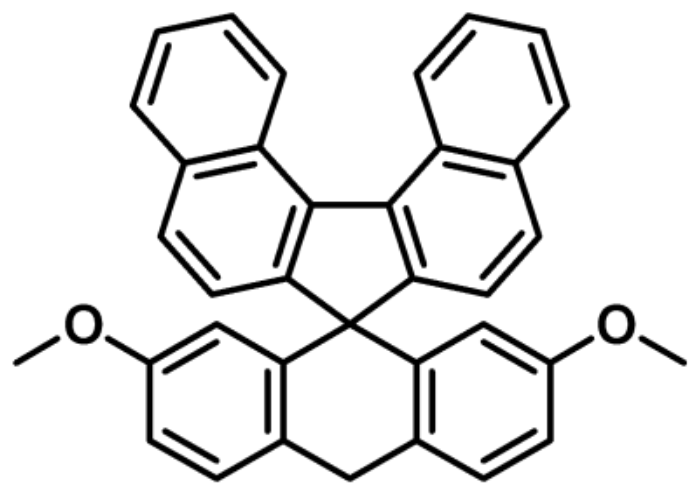

Following the procedure used for the synthesis of *rac*-**3a** and using *n*-hexane/EtOAc 9:1 as eluent in flash chromatography, compound *rac*-**3b** (75 mg, 0.15 mmol, 43%) was obtained as a pinkish solid from the corresponding bromo derivative **2**[S1] (438 mg, 1.42 mmol, 4 equiv.) and the fluorenone *rac*-**1b**[S1] (100 mg, 0.35 mmol, 1 equiv.).

¹H NMR (500 MHz, $CDCl_3$) δ 8.89 (d, *J* = 8.6 Hz, 2H), 7.97 (d, *J* = 8.1 Hz, 2H), 7.79 (d, *J* = 8.2 Hz, 2H), 7.68 (t, *J* = 7.0 Hz, 2H), 7.59 (t, *J* = 6.9 Hz, 2H), 7.39 (d, *J* = 8.5 Hz, 2H), 7.29 (d, *J* = 8.2 Hz, 2H), 6.81 (dd, *J* = 8.5, 2.7 Hz, 2H), 6.02 (d, *J* = 2.7 Hz, 2H), 4.52 (s, 2H), 3.43 (s, 6H). **¹³C NMR** (126 MHz, $CDCl_3$) δ 158.1 (C), 155.1 (C), 138.5 (C), 136.9 (C), 134.5 (C), 129.6 (CH), 129.4 (CH), 129.0 (CH), 128.9 (C), 127.4 (CH), 127.4 (C), 125.3 (CH), 125.3 (CH), 123.0 (CH), 112.9 (CH), 112.5 (CH), 60.9 (C), 55.0 (CH_3), 33.5 (CH_2). **HRMS** (ESI) m/z: $C_{36}H_{26}O_2Na$ $[M+Na]^+$ calcd: 513.1830; found: 513.1844.

Compound *rac*-4a

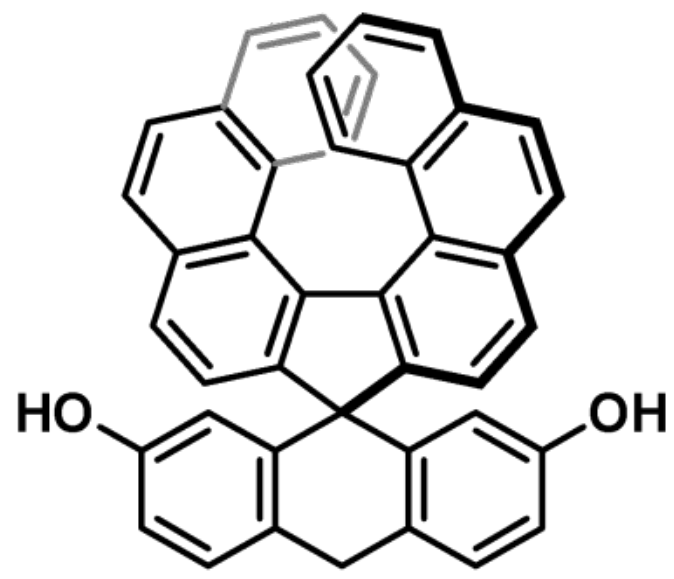

Compound *rac*-**3a** (105 mg, 0.17 mmol, 1 equiv.) was dissolved in anhydrous CH_2Cl_2 (1 mL) under an argon atmosphere. Boron tribromide 1M in CH_2Cl_2 (0.44 mL, 0.44 mmol, 2.5 equiv.) was added dropwise at 0 °C and the reaction changed color to dark purple. The mixture was stirred at room temperature for 3 h. Then, the reaction was neutralized with a saturated $NaHCO_3$ aqueous solution and the aqueous layer extracted with CH_2Cl_2. The organic phase was dried over anhydrous Na_2SO_4, filtered, and the solvent was concentrated under reduced pressure. The product was purified by flash column chromatography on silica gel using *n*-hexane/EtOAc 8:2 → *n*-hexane/EtOAc 7:3 as eluent, affording compound *rac*-**4a** (90 mg, 0.15 mmol, 90%) as a green-grey solid. NOTE: This compound is prone to be oxidized under ambient atmosphere to the ketone (CH_2 to CO, minor impurities in the NMR spectra shown).

¹H NMR (500 MHz, $CDCl_3$) δ 7.78 – 7.75 (m, 4H), 7.74 – 7.71 (m, 4H), 7.65 (d, J = 6.7 Hz, 2H), 7.33 (d, *J* = 3.1 Hz, 2H), 7.31 (d, *J* = 3.8 Hz, 2H), 7.14 (ddd, *J* = 8.0, 7.0, 1.2 Hz, 2H), 6.70 (dd, *J* = 8.3, 2.7 Hz, 2H), 6.35 (ddd, *J* = 8.4, 6.9, 1.4 Hz, 2H), 5.99 (d, *J* = 2.7 Hz, 2H), 4.48 (s, 2H), 4.29 (s, 2H). **¹³C NMR** (126 MHz, $CDCl_3$) δ 156.2 (C), 154.0 (C), 138.8 (C), 137.1 (C), 132.5 (C), 131.8 (C), 131.0 (C), 130.1 (CH), 129.5 (CH), 127.9 (C), 127.7 (CH), 127.4 (C), 127.0 (CH), 126.9 (CH), 126.8 (CH), 126.6 (CH), 123.4 (CH), 123.2 (CH), 115.0 (CH), 113.6 (CH), 59.9 (C), 33.2 (CH_2). **HRMS** (ESI) $C_{42}H_{25}O_2$ $[M-H]^+$ calcd: 561.1855; found: 561.1857.

Compound *rac*-4b

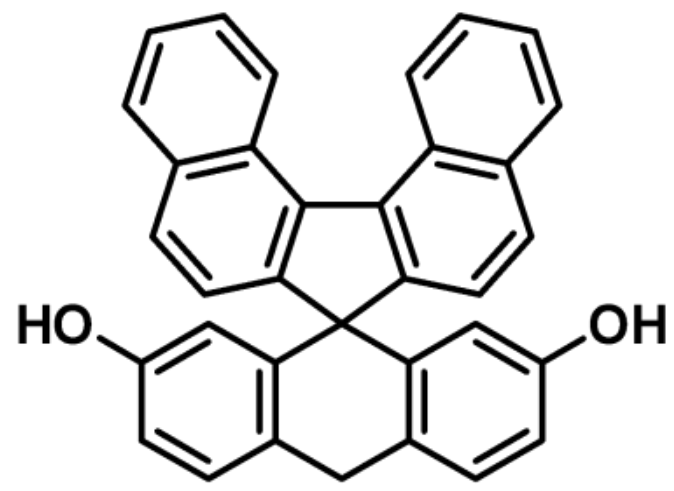

Following the procedure used for the synthesis of *rac*-**4a**, compound *rac*-**4b** (100 mg, 0.21 mmol, 76%) was obtained as a reddish solid from *rac*-**3b** (140 mg, 0.28 mmol, 1 equiv.) and BBr_3 (1M in DCM, 0.71 mL, 0.71 mmol, 1 equiv.). In this case, a gradient from *n*-hexane/EtOAc 8:2 to 7:3 was used as eluent in flash chromatography .

^{1}**H NMR** (400 MHz, $CDCl_3$) δ 8.82 (d, *J* = 8.5 Hz, 2H), 7.93 (d, *J* = 8.1 Hz, 2H), 7.74 (d, *J* = 8.3 Hz, 2H), 7.64 (t, *J* = 7.7 Hz, 2H), 7.55 (t, *J* = 7.5 Hz, 2H), 7.27 (d, *J* = 8.2 Hz, 2H), 7.20 (d, *J* = 8.2 Hz, 2H), 6.67 (dd, *J* = 8.3, 2.7 Hz, 2H), 5.82 (d, *J* = 2.6 Hz, 2H), 4.42 (s, 2H), 4.24 (s, 2H). ^{13}**C NMR** (101 MHz, $CDCl_3$) δ 155.1 (C), 154.0 (C), 138.2 (C), 136.8 (C), 134.5 (C), 130.0 (CH), 129.5 (CH), 129.0 (CH), 128.8 (C), 127.4 (CH), 127.3 (C), 125.5 (CH), 125.4 (CH), 123.1 (CH), 115.0 (CH), 113.3 (CH), 60.5 (C), 33.2 (CH_2). **HRMS** (ESI) m/z: $C_{34}H_{21}O_2$ $[M-H]^+$ calcd: 461.1542; found: 461.1543.

Compound *rac*-5a

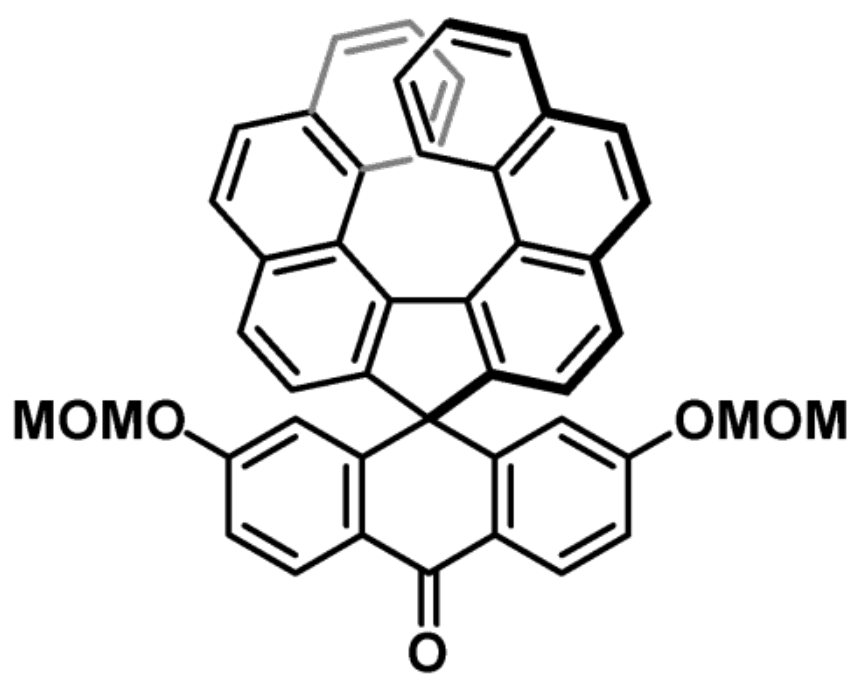

To a solution of compound *rac*-**4a** (90 mg, 0.16 mmol, 1 equiv.) in anhydrous DMF (6 mL) and under an argon atmosphere, NaH (60%) (25 mg, 0.64 mmol, 4 equiv.) was added at 0 °C. The mixture was stirred at this temperature for 30 min and the colour changed from yellow to red. Then, MOM-Cl (64 mg, 0.80 mmol, 5 equiv.) was added. The resulting solution was stirred at room temperature for 3 h. The reaction was quenched with a few drops of H_2O at 0 °C. Then, EtOAc was added, and the organic phase washed with brine. The organic phase was dried over anhydrous Na_2SO_4, filtered, and the solvent was concentrated under reduced pressure. The

product was purified by flash column chromatography on silica gel using *n*-hexane/EtOAc 9:1 as eluent, affording a beige solid (52 mg, 0.08 mmol, 50%).

Subsequently, the residue obtained (52 mg, 0.07 mmol, 1 equiv.) was dissolved in anhydrous CH_3CN (4 mL) and $KMnO_4$ (37 mg, 0.23 mmol, 3 equiv.) was added. The mixture was stirred at 60 ºC overnight. The reaction was quenched with a few drops of H_2O, and CH_3CN was removed. Then, CH_2Cl_2 was added, and the organic phase washed with a saturated $Na_2S_2O_3$ aqueous solution. The organic phase was dried over anhydrous Na_2SO_4, filtered, and the solvent was concentrated under reduced pressure. The product was purified by flash column chromatography on silica gel using *n*-hexane/EtOAc 8:2 as eluent, affording compound *rac*-**5a** (40 mg, 0.06 mmol, 85%) as a beige solid.

^{1}H NMR (500 MHz, $CDCl_3$) δ 8.48 (d, *J* = 8.9 Hz, 2H), 7.78 (d, *J* = 7.9 Hz, 2H), 7.76 – 7.73 (m, 6H), 7.67 (dd, *J* = 7.9, 1.4 Hz, 2H), 7.22 (d, *J* = 7.8 Hz, 2H), 7.17 (ddd, *J* = 8.0, 7.0, 1.2 Hz, 2H), 7.12 (dd, *J* = 8.9, 2.4 Hz, 2H), 6.38 (ddd, *J* = 8.2, 7.0, 1.4 Hz, 2H), 6.27 (d, *J* = 2.4 Hz, 2H), 4.86 (s, 4H), 3.16 (s, 6H). **^{13}C NMR** (126 MHz, $CDCl_3$) δ 182.5 (CO), 161.4 (C), 152.8 (C), 146.3 (C), 138.0 (C), 132.8 (C), 131.8 (C), 130.8 (C), 130.4 (CH), 129.8 (CH), 128.1 (C), 127.7 (CH), 127.3 (CH), 126.9 (CH), 126.9 (CH), 126.7 (C), 126.6 (CH), 123.6 (CH), 122.7 (CH), 115.1 (CH), 115.1 (CH), 94.3 (CH_2), 58.6 (C), 56.3 (CH_3). **HRMS** (ESI) $C_{46}H_{32}O_5Na$ $[M+Na]^+$ calcd: 687.2147; found: 687.2155.

Compound *rac*-5b

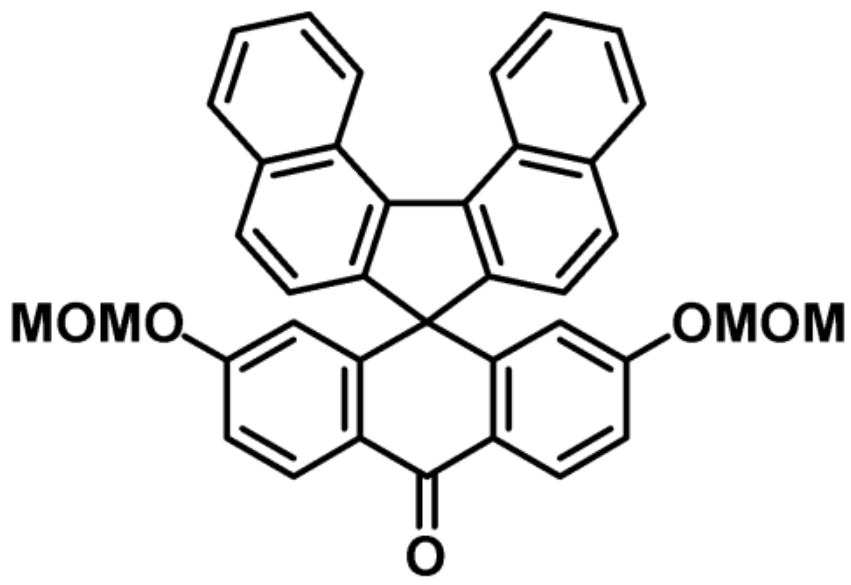

Following the procedure used for the synthesis of *rac*-**5a** and using *n*-hexane/EtOAc 8:2 as eluent in flash chromatography, compound *rac*-**5b** (25 mg, 0.04 mmol, 73%) was obtained as a beige solid from *rac*-**4b** (30 mg, 0.06 mmol, 1 equiv.) using NaH 60% (10 mg, 0.25 mmol, 4 equiv.) and MOM-Cl (25 mg, 0.31 mmol, 5 equiv.).

^{1}H NMR (500 MHz, $CDCl_3$) δ 8.85 (d, *J* = 8.5 Hz, 2H), 8.44 (d, *J* = 8.8 Hz, 2H), 7.95 (d, *J* = 6.9 Hz, 2H), 7.76 (d, *J* = 8.2 Hz, 2H), 7.68 (t, *J* = 7.0 Hz, 2H), 7.59 (t, *J* = 7.0 Hz, 2H), 7.10 (d, *J* = 3.0 Hz, 2H), 7.08 (d, *J* = 3.8 Hz, 2H), 6.07 (d, *J* = 2.2 Hz, 2H), 4.88 (s, 4H), 3.19 (s, 6H). **^{13}C NMR** (126 MHz, $CDCl_3$) δ 182.5 (CO), 161.3 (C), 151.8 (C), 145.5 (C), 138.0 (C), 134.7 (C), 130.3 (CH), 129.9 (CH),

129.1 (CH), 128.9 (C), 127.4 (CH), 126.7 (C), 125.8 (CH), 125.6 (CH), 122.4 (CH), 115.1 (CH), 114.7 (CH), 94.2 (CH_2), 59.2 (C), 56.3 (CH_3). **HRMS** (ESI) m/z: $C_{36}H_{24}O_3Na$ $[M+Na]^+$ calcd: 527.1623; found: 527.1631.

Compound *rac*-7H-CF

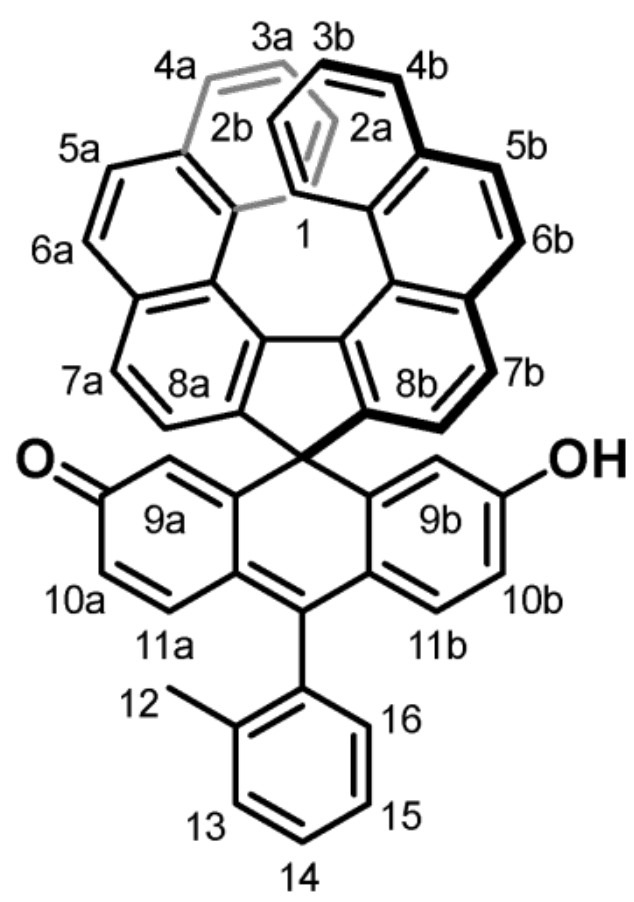

To a solution of 2-bromotoluene (0.02 mL, 0.24 mmol, 4 equiv.) in anhydrous THF (1 mL) and under an argon atmosphere, *n*-BuLi 2.5 M in *n*-hexane (0.08 mL, 0.21 mmol, 3.5 equiv.) was added at -78 °C. The mixture was stirred at this temperature for 45 min and it changed color to orange. Then, the lithiated derivative was added dropwise to a solution of the ketone *rac*-**5a** (40 mg, 0.06 mmol, 1 equiv.) dissolved in anhydrous THF (0.5 mL) and under an argon atmosphere. The resulting solution was stirred at room temperature for 3 h and it changed color to brown. The reaction was quenched with a few drops of H_2O at 0 °C, and THF was removed. Then, EtOAc was added, and the organic phase washed with brine. The organic phase was dried over anhydrous Na_2SO_4, filtered, and the solvent was concentrated under reduced pressure.

Subsequently, the residue obtained was dissolved in anhydrous MeOH (1 mL) and a HCl aqueous solution of increasing concentration 1N → 3N → 6N (1 mL) was added while monitoring the reaction by TLC. Generally, the deprotection of the MOM group and the formation of the quinone was obtained by adding 6N HCl and stirring overnight, otherwise the reaction could be heated to 60 °C. Then, the reaction was neutralized with a saturated $NaHCO_3$ aqueous solution and the aqueous layer extracted with EtOAc. The organic phase was dried over anhydrous Na_2SO_4, filtered, and the solvent was concentrated under reduced pressure. The product was purified by preparative thin layer chromatography using *n*-hexane/EtOAc 7:3 as eluent, affording compound *rac*-**7H-CF** (12 mg, 0.01 mmol, 30%) as a purple solid.

^{1}H NMR (600 MHz, MeOD) δ 7.93 (d, *J* = 7.9 Hz, 1H, 7a-CH), 7.91 (d, *J* = 7.9 Hz, 1H, 7b-CH), 7.82 (d, *J* = 3.6 Hz, 1H, 6a-CH), 7.81 (d, *J* = 3.5 Hz, 1H, 6b-CH), 7.78 (d, *J* = 2.2 Hz, 1H, 5a-CH), 7.76 (d,

J = 2.3 Hz, 1H, 5b-CH), 7.73 (d, *J* = 3.6 Hz, 1H, 1a-CH), 7.72 (d, *J* = 3.5 Hz, 1H, 1b-CH), 7.67 (d, *J* = 7.9 Hz, 2H, 4-CH), 7.55 – 7.52 (m, 2H, 13-CH and 15-CH), 7.50 (td, *J* = 6.5, 5.7, 2.7 Hz, 1H, 14-CH), 7.47 – 7.44 (m, 2H, 16-CH and 8a-CH), 7.40 (d, *J* = 7.9 Hz, 1H, 8b-CH), 7.13 (t, *J* = 6.8 Hz, 2H, 2-CH), 7.02 (dd, *J* = 9.4, 2.5 Hz, 2H, 10-CH), 6.33 – 6.28 (m, 4H, 3-CH and 11-CH), 6.00 (d, *J* = 2.1 Hz, 2H, 9-CH), 2.41 (s, 3H, 12-CH_3).Signals appearing at 3.34 and 4.88 correspond to traces of MeOH and H_2O respectively. **^{13}C NMR** (151 MHz, MeOD) δ 182.5 (CO), 163.5 (C), 155.2 (C), 155.0 (C), 151.4 (C), 139.0 (C), 138.9 (C), 138.5 (10-CH), 138.5 (10-CH), 138.0 (C), 137.2 (C), 134.0 (C), 134.0 (C), 133.2 (C), 132.0 (C), 131.3 (15-CH), 130.9 (7-CH), 130.9 (7-CH), 130.4 (16-CH), 130.0 (14-CH), 129.3 (C), 129.2 (C), 128.4 (1-CH), 128.1 (5-CH), 127.8 (6-CH and 2-CH), 127.6 (4-CH), 126.9 (13-CH), 124.4 (3-CH), 123.2 (11-CH), 123.2 (8-CH), 123.0 (8-CH), 122.4 (9-CH), 120.8 (C), 61.7 (spiro-C), 20.1 (12-CH_3). **HRMS** (ESI) $C_{49}H_{29}O_2$ $[M-H]^+$ calcd: 649.2168; found: 649.2171.

Compound *rac*-5H-CF

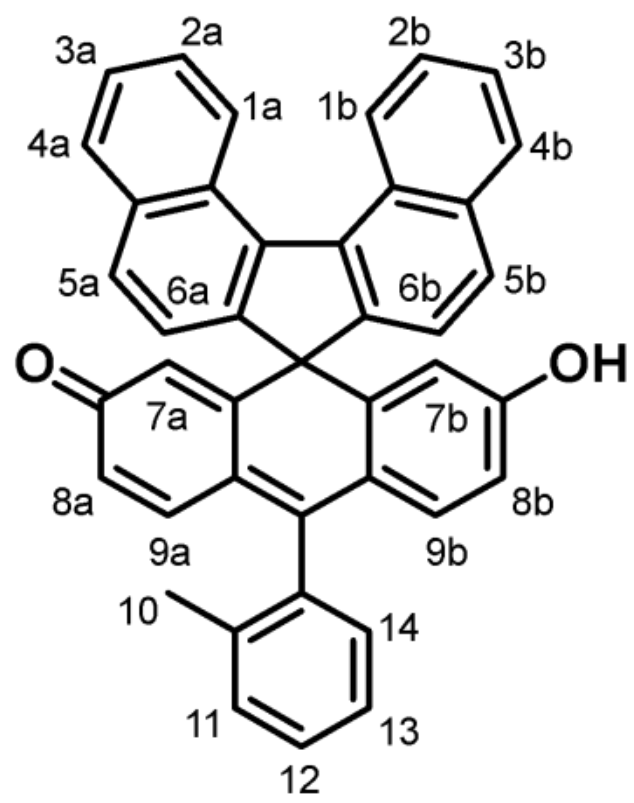

Following the procedure used for the synthesis of *rac*-**7H-CF** and using *n*-hexane/EtOAc 7:3 as eluent in flash chromatography, compound *rac*-**5H-CF** (8 mg, 0.01 mmol, 36%) was obtained as a purple solid from 2-bromotoluene (0.02 mL, 0.17 mmol, 4 equiv.) and *rac*-**5b** (25 mg, 0.04 mmol, 1 equiv.).

^{1}H NMR (600 MHz, MeOD) δ 8.78 (d, *J* = 8.6 Hz, 2H, 1-CH), 8.00 (d, *J* = 8.3 Hz, 2H, 4-CH), 7.89 (d, *J* = 9.0 Hz, 2H, 5-CH), 7.70 – 7.64 (m, 2H, 2-CH), 7.61 – 7.56 (m, 2H, 3-CH), 7.53 – 7.50 (m, 2H, 11-CH and 13-CH), 7.48 (td, *J* = 6.8, 2.5 Hz, 1H, 12-CH), 7.41 (d, *J* = 6.9 Hz, 1H, 14-CH), 7.31 (d, *J* = 8.3 Hz, 1H, 6a-CH), 7.27 (d, *J* = 8.2 Hz, 1H, 6b-CH), 6.98 (d, *J* = 9.3 Hz, 2H, 9-CH), 6.27 (dd, *J* = 9.4, 2.3 Hz, 2H, 8-CH), 5.77 (d, *J* = 2.3 Hz, 2H, 7-CH), 2.37 (s, 3H, 10-CH_3). Signals appearing at 3.34 and 4.88 correspond to traces of MeOH and H_2O respectively. Singlet at 1.90 ppm likely corresponds to trace amounts of acetic acid. **^{13}C NMR** (151 MHz, MeOD) δ 182.0 (CO), 163.2 (C), 154.1 (C), 154.0 (C), 150.4 (C), 138.8 (C), 138.7 (C), 138.4 (9-CH), 137.9 (C), 137.2 (C), 136.1

(C), 136.0 (C), 131.3 (13-CH), 131.1 (5-CH), 131.0 (5-CH), 130.3 (14-CH), 130.1 (12-CH), 130.1 (C), 130.1 (4-CH), 128.0 (1-CH), 126.9 (11-CH), 126.7 (3-CH), 126.6 (2-CH), 123.2 (8-CH), 122.9 (6-CH), 122.8 (6-CH), 121.8 (7-CH), 120.9 (C), 62.3 (spiro-C), 20.0 (10-CH_3). Peaks at 180.5 and 24.0 ppm likely correspond to trace amounts of acetic acid. **HRMS** (ESI) m/z: $C_{41}H_{25}O_2$ $[M-H]^+$ calcd: 549.1855; found 549.1855.

1.3. ^{1}H- and ^{13}C-NMR Spectra and HRMS spectra of 5H-CF and 7H-CF

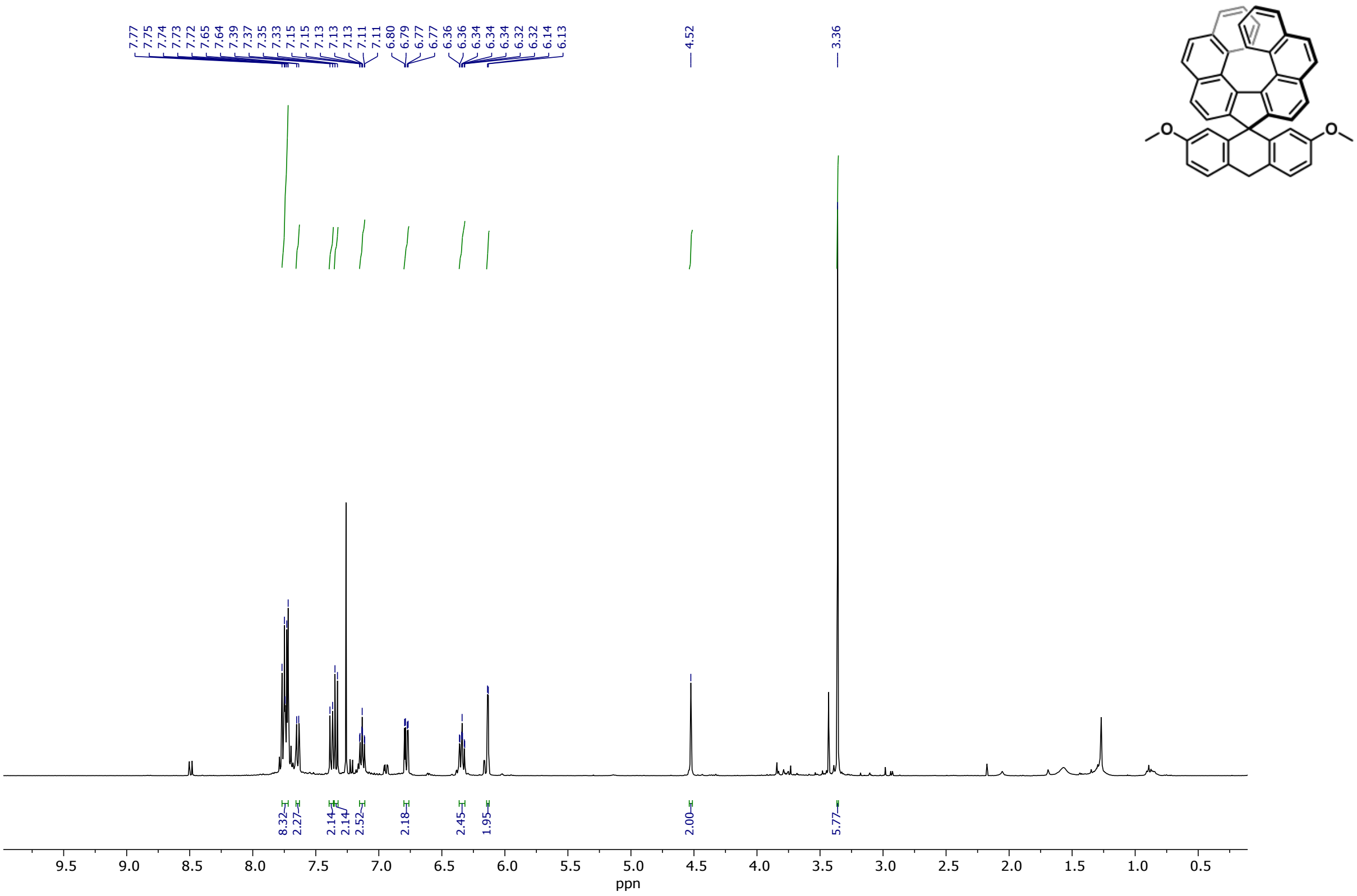

Figure S1. ^{1}H-NMR (400 MHz, $CDCl_3$) spectrum of compound *rac*-**3a**.

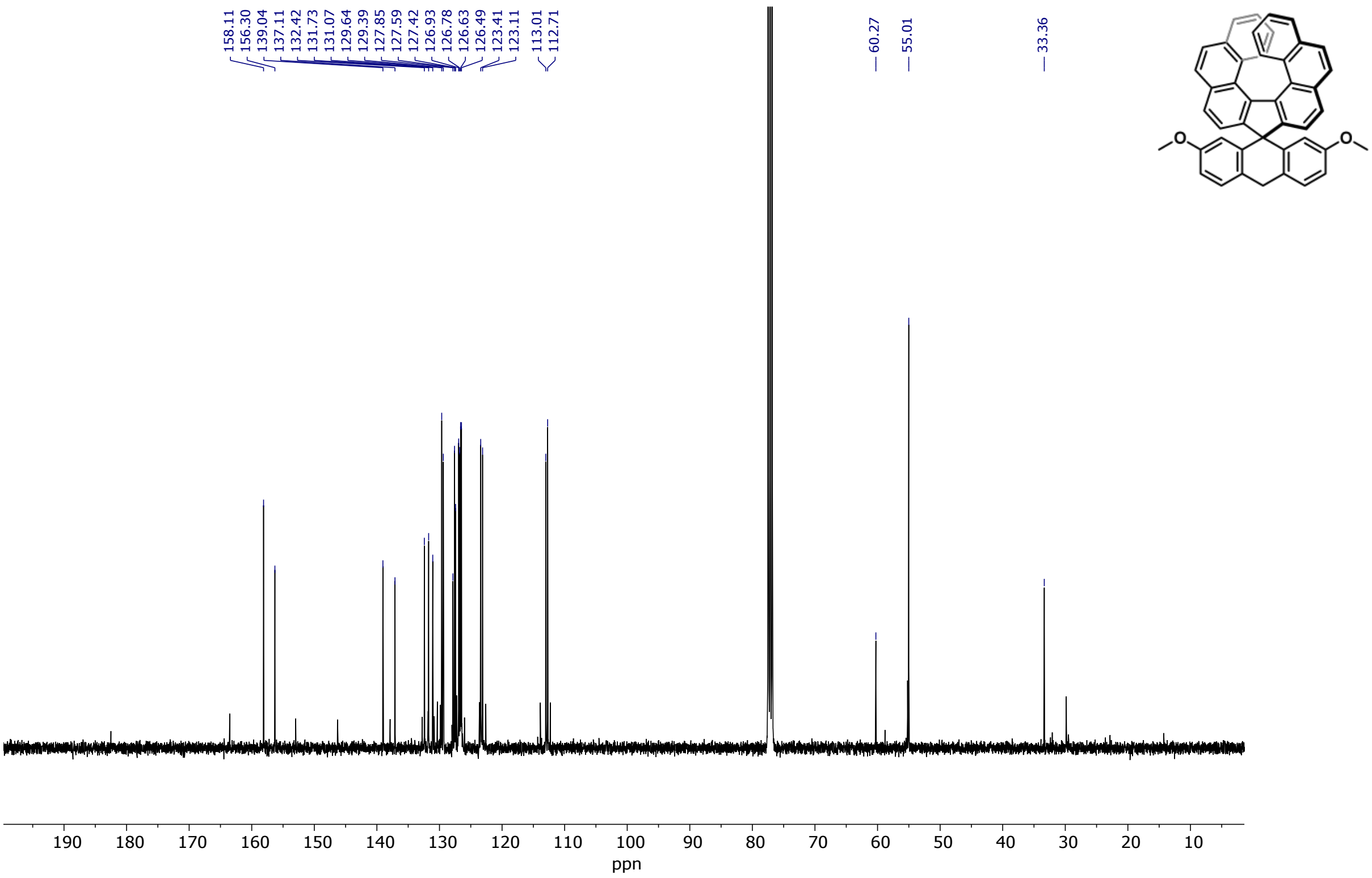

Figure S2. ^{13}C-NMR (101 MHz, $CDCl_3$) spectrum of compound *rac*-**3a.**

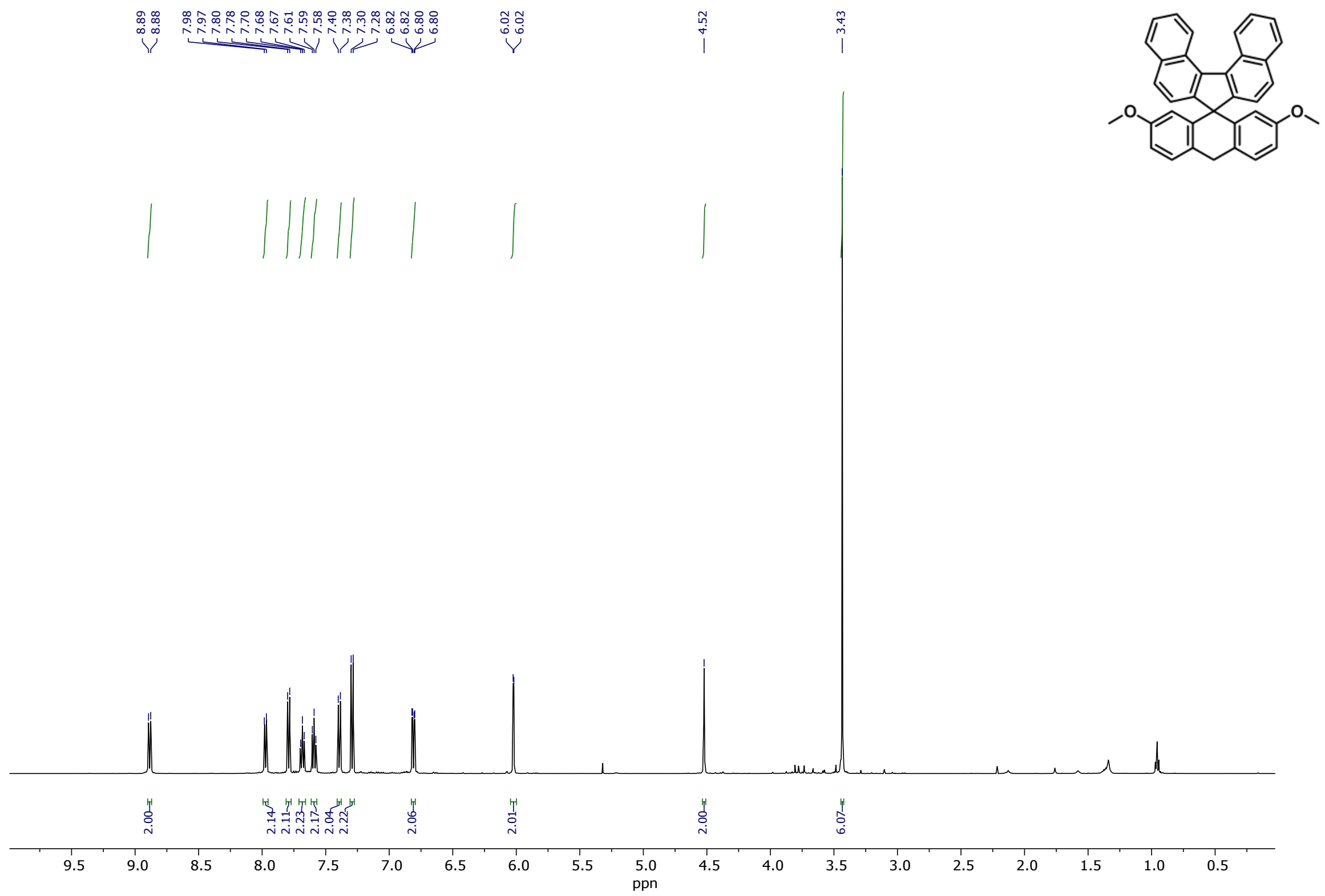

Figure S3. ^{1}H-NMR (500 MHz, $CDCl_3$) spectrum of compound *rac*-**3b**.

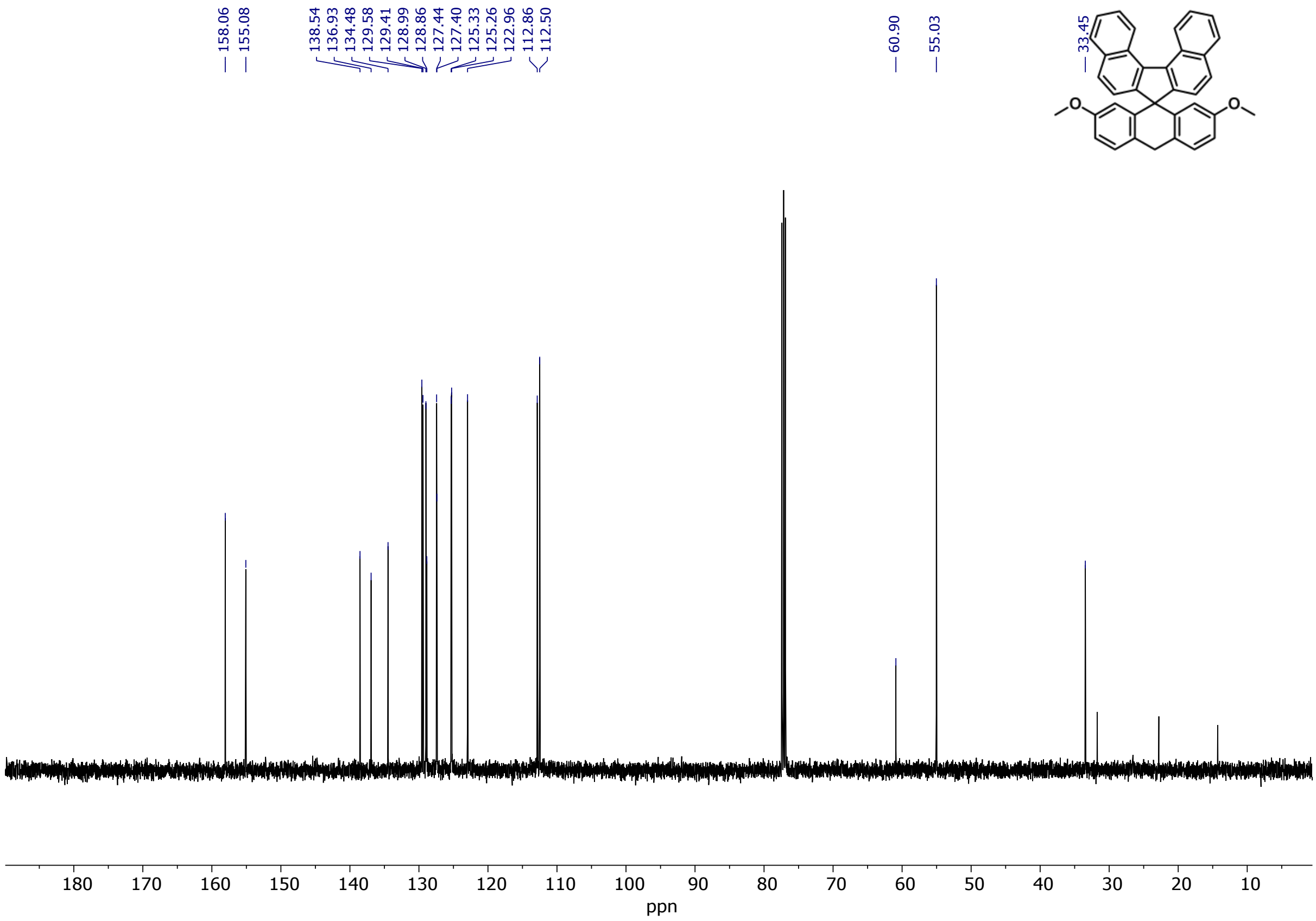

Figure S4. ^{13}C-NMR (126 MHz, $CDCl_3$) spectrum of compound *rac*-**3b**.

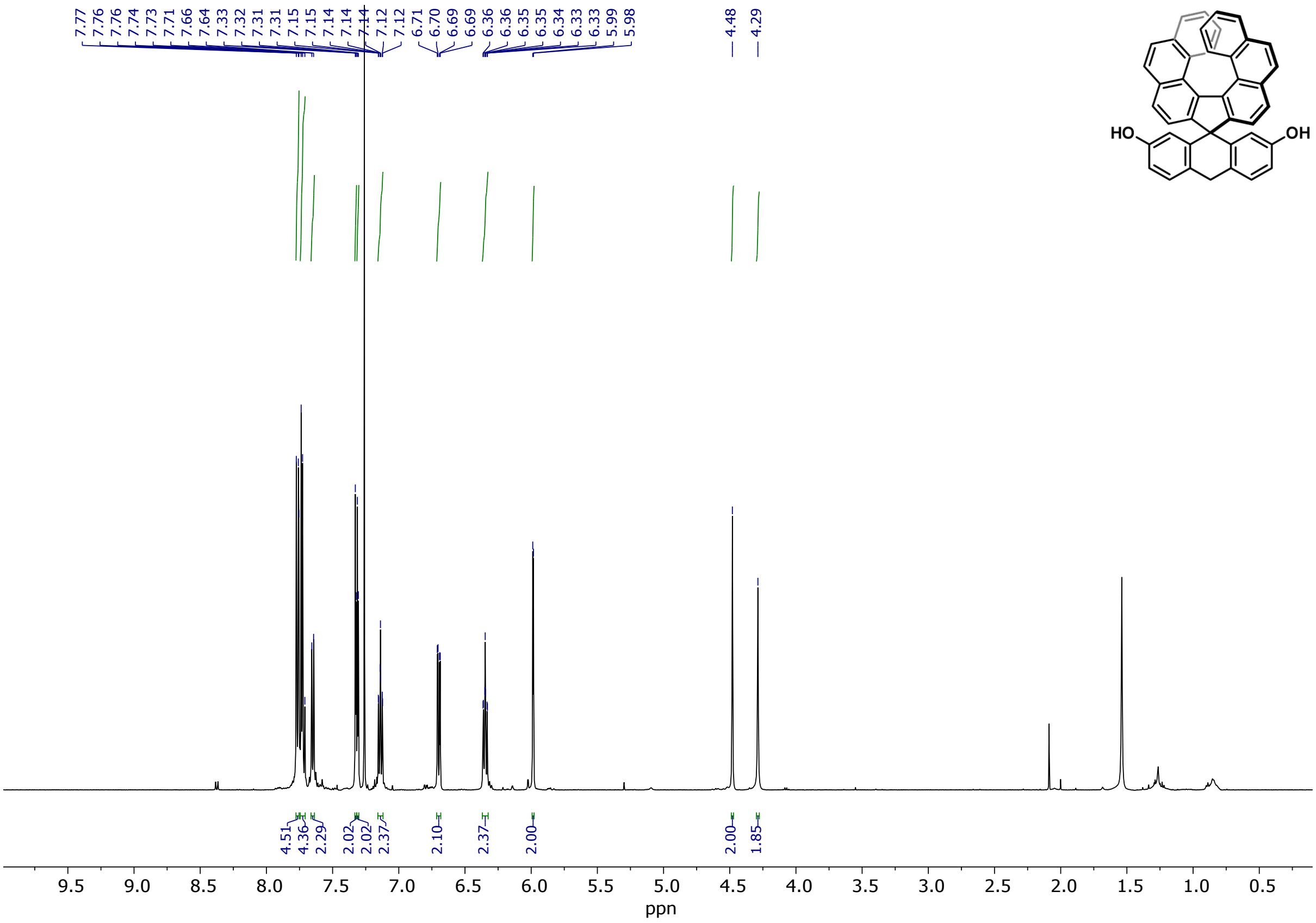

Figure S5. ^{1}H-NMR (500 MHz, $CDCl_3$) spectrum of compound *rac*-**4a**.

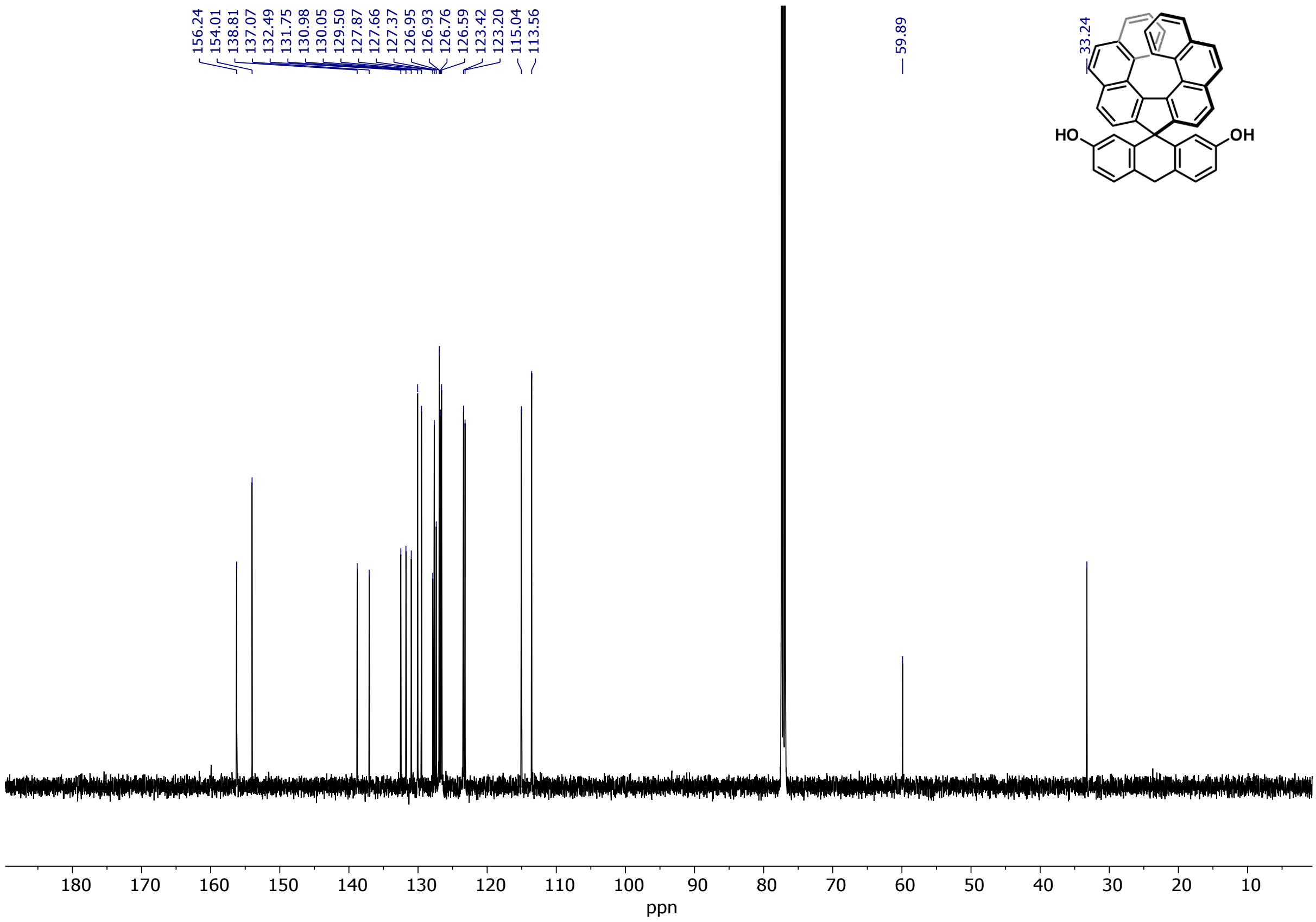

Figure S6. ^{13}C-NMR (126 MHz, $CDCl_3$) spectrum of compound *rac*-**4a**.

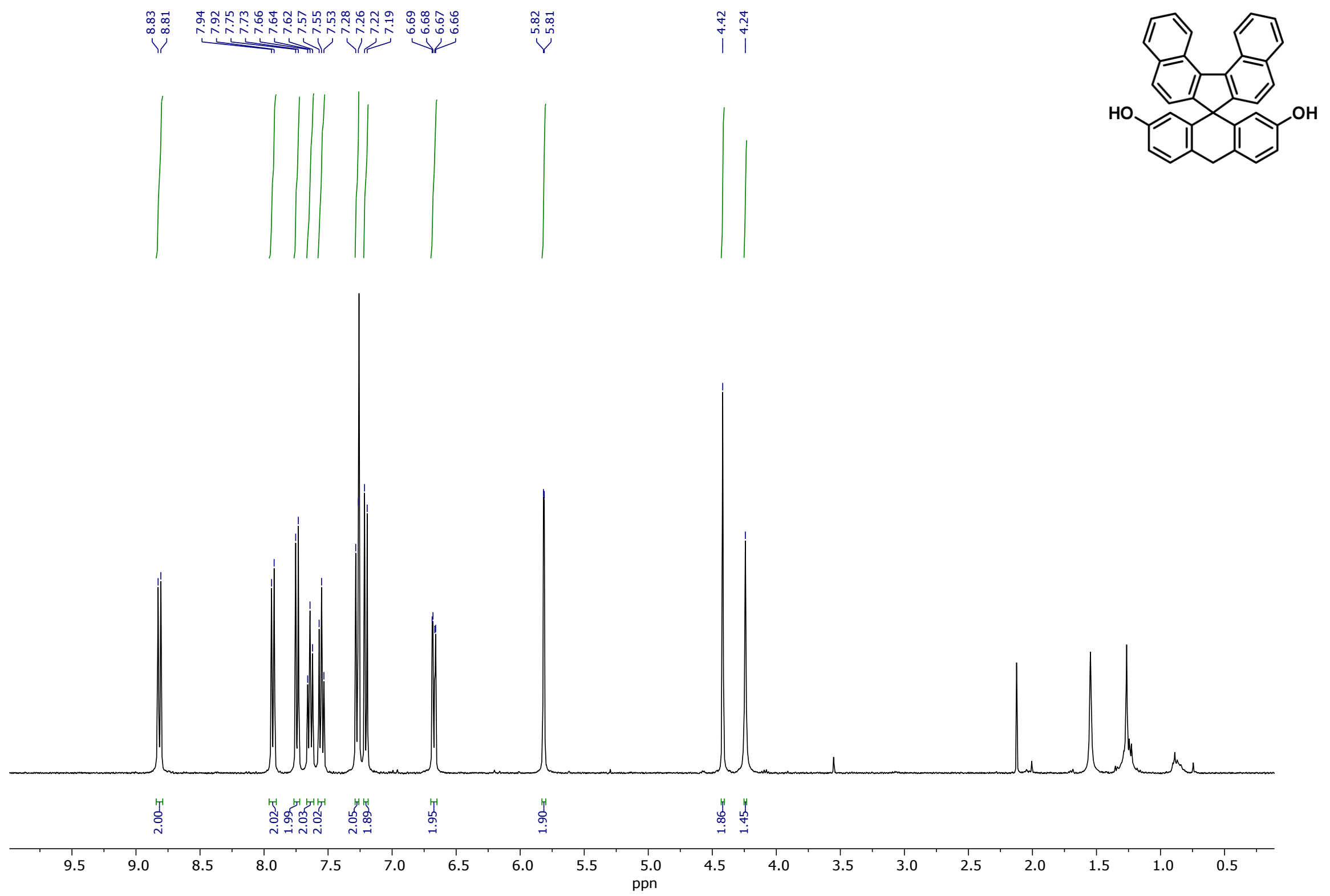

Figure S7. ^{1}H-NMR (400 MHz, $CDCl_3$) spectrum of compound *rac*-**4b**.

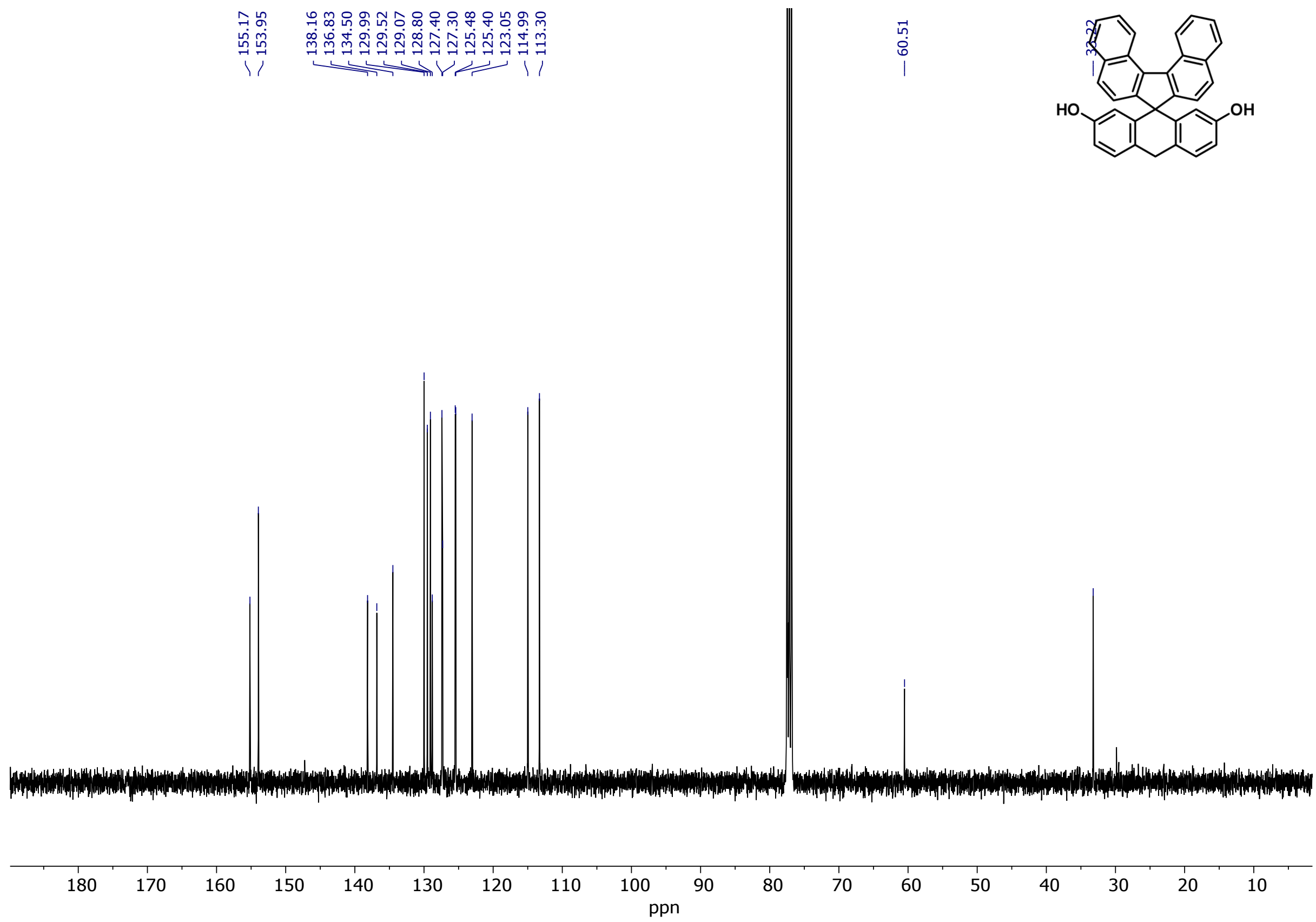

Figure S8. ^{13}C-NMR (101 MHz, $CDCl_3$) spectrum of compound *rac*-**4b**.

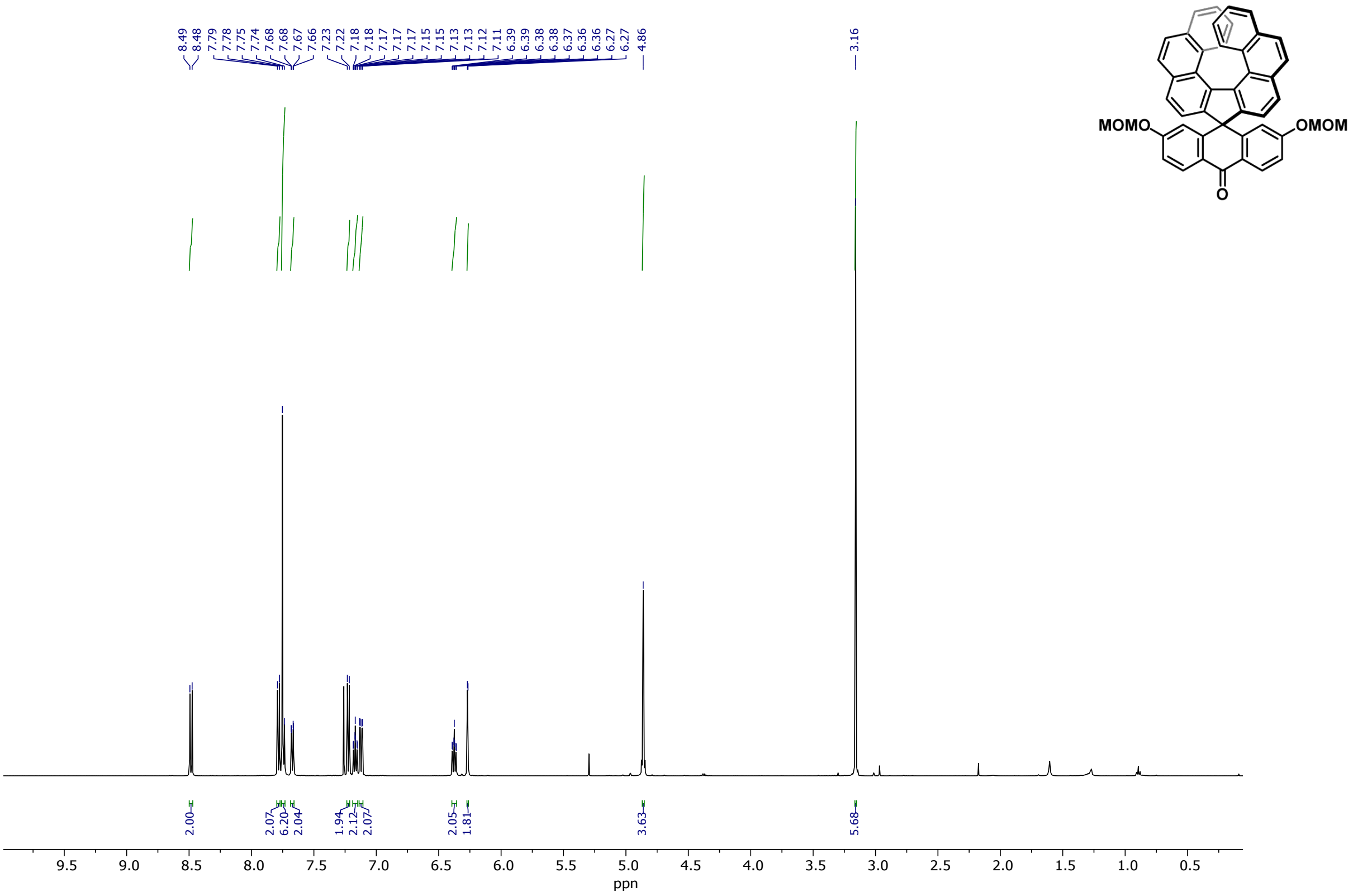

Figure S9. ^{1}H-NMR (500 MHz, $CDCl_3$) spectrum of compound *rac*-**5a**.

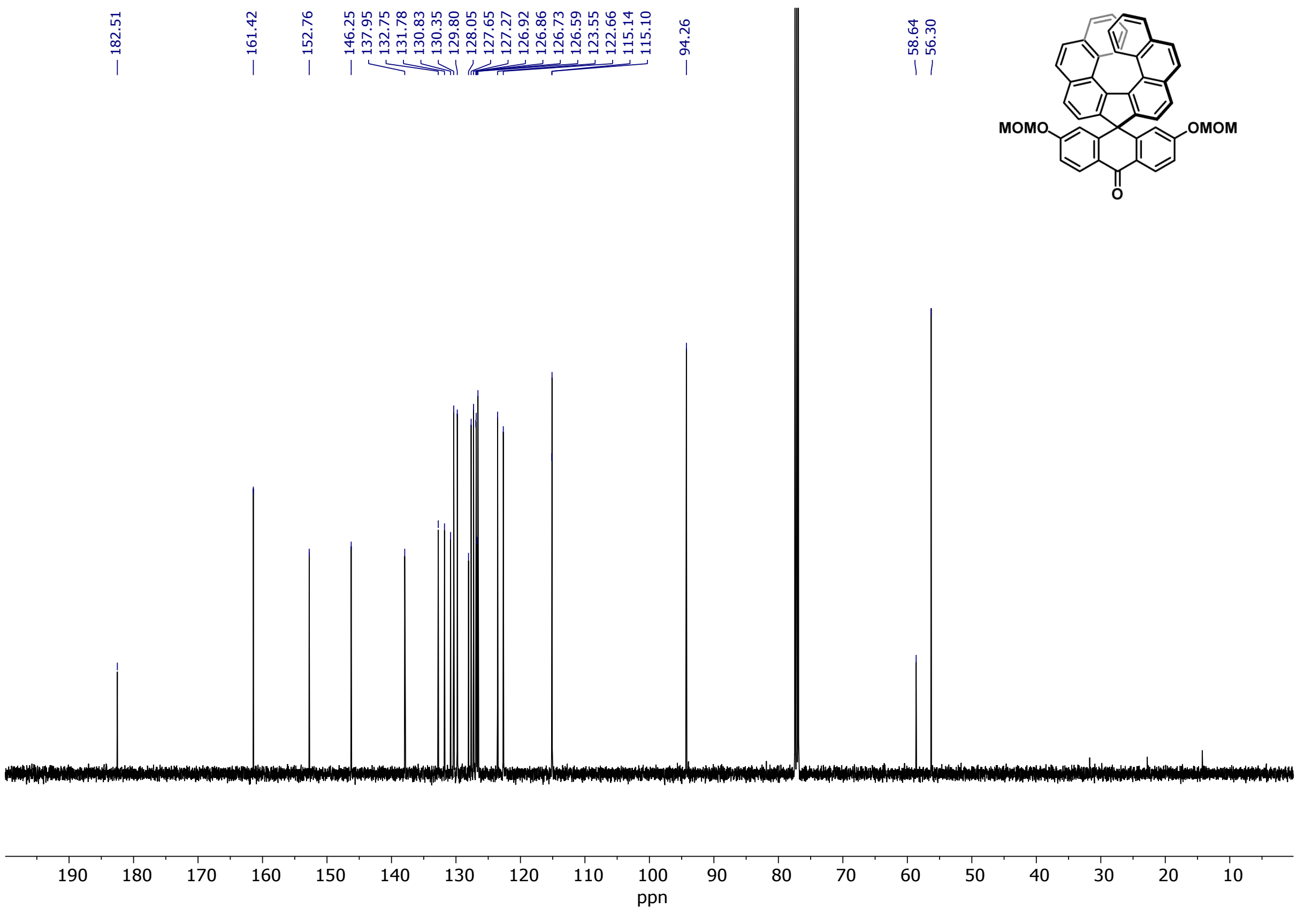

Figure S10. ^{13}C-NMR (126 MHz, $CDCl_3$) spectrum of compound *rac*-**5a**.

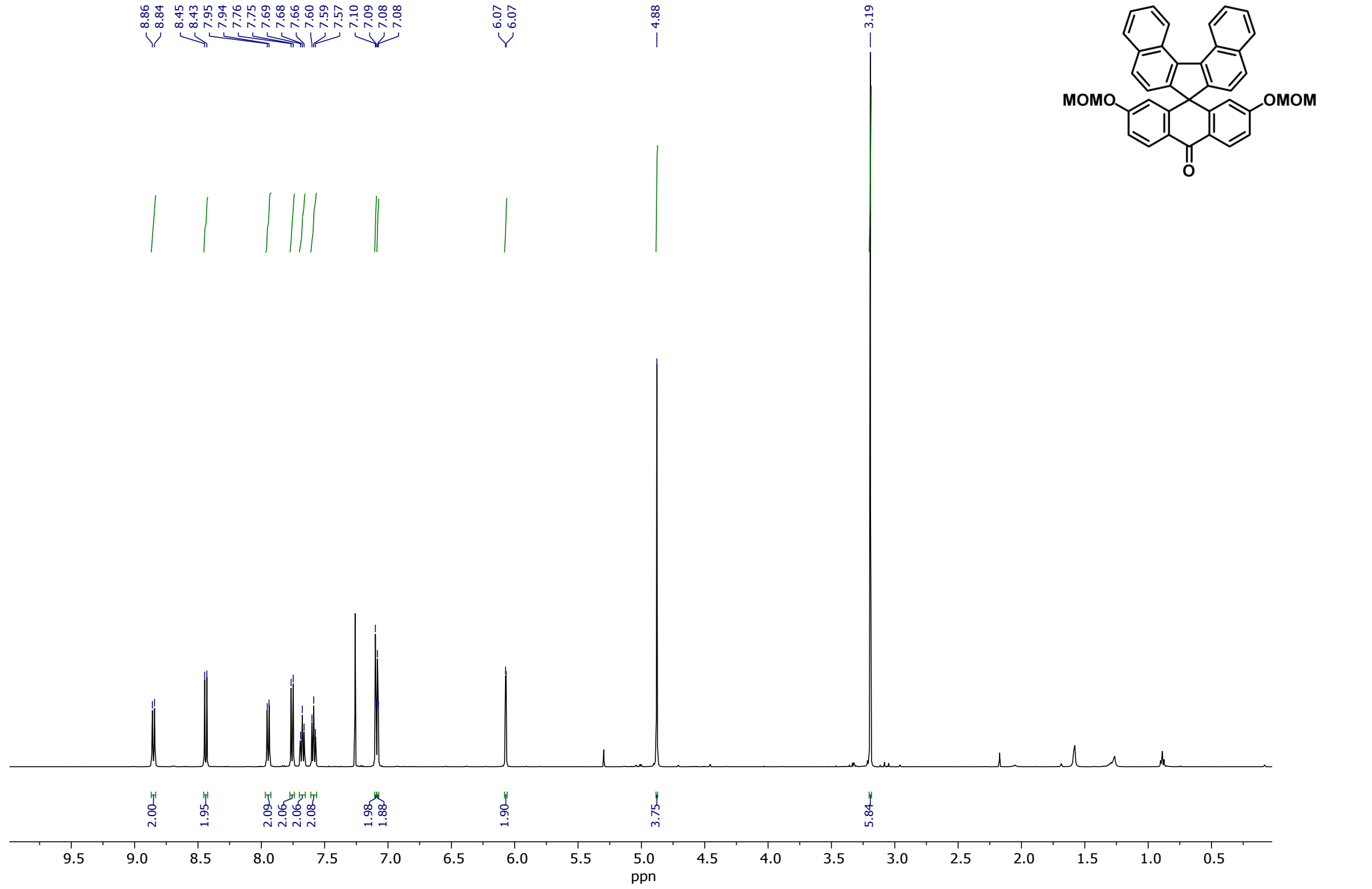

Figure S11. ^{1}H-NMR (500 MHz, $CDCl_3$) spectrum of compound *rac*-**5b**.

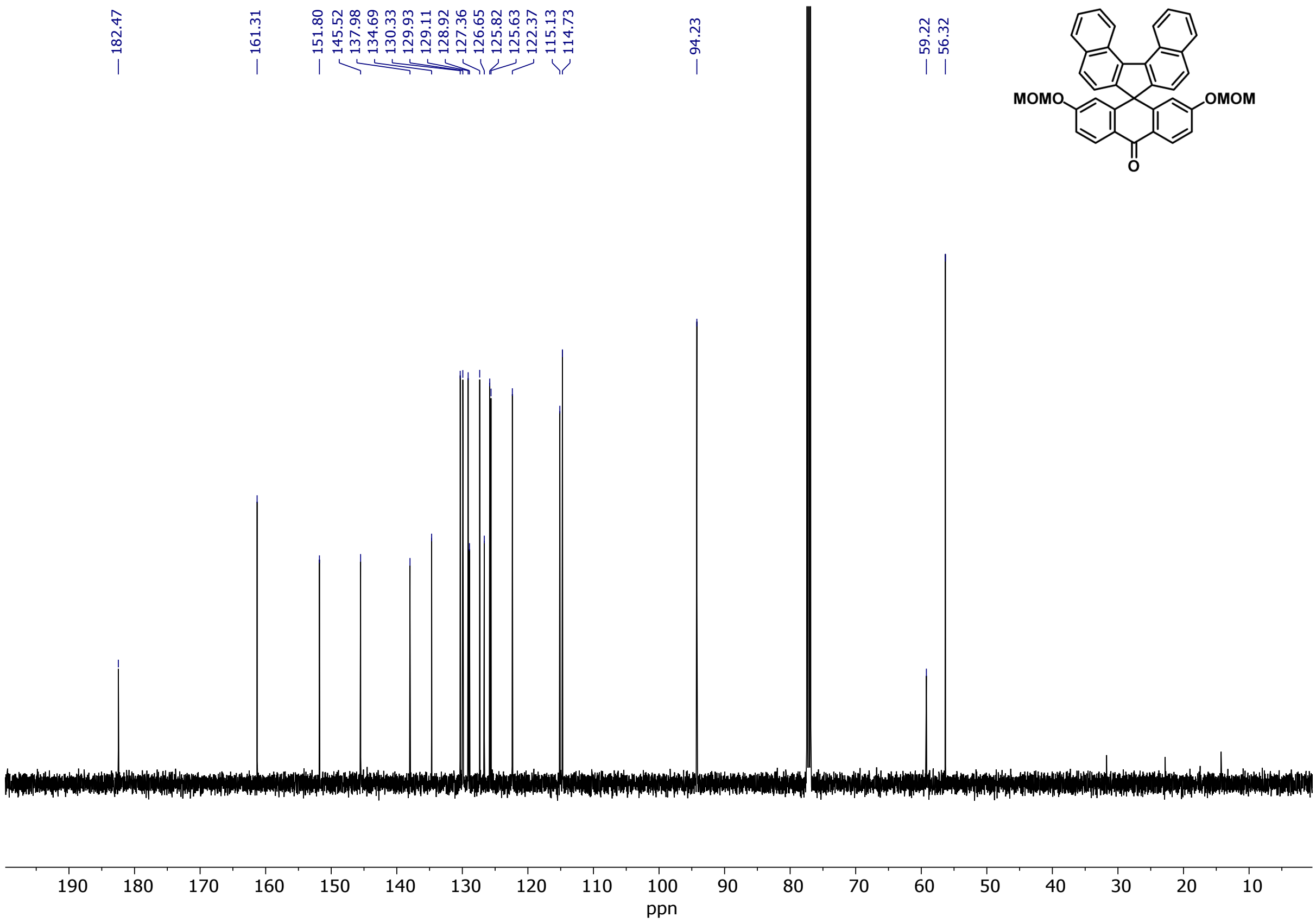

Figure S12. ^{13}C-NMR (126 MHz, $CDCl_3$) spectrum of compound *rac*-**5b**.

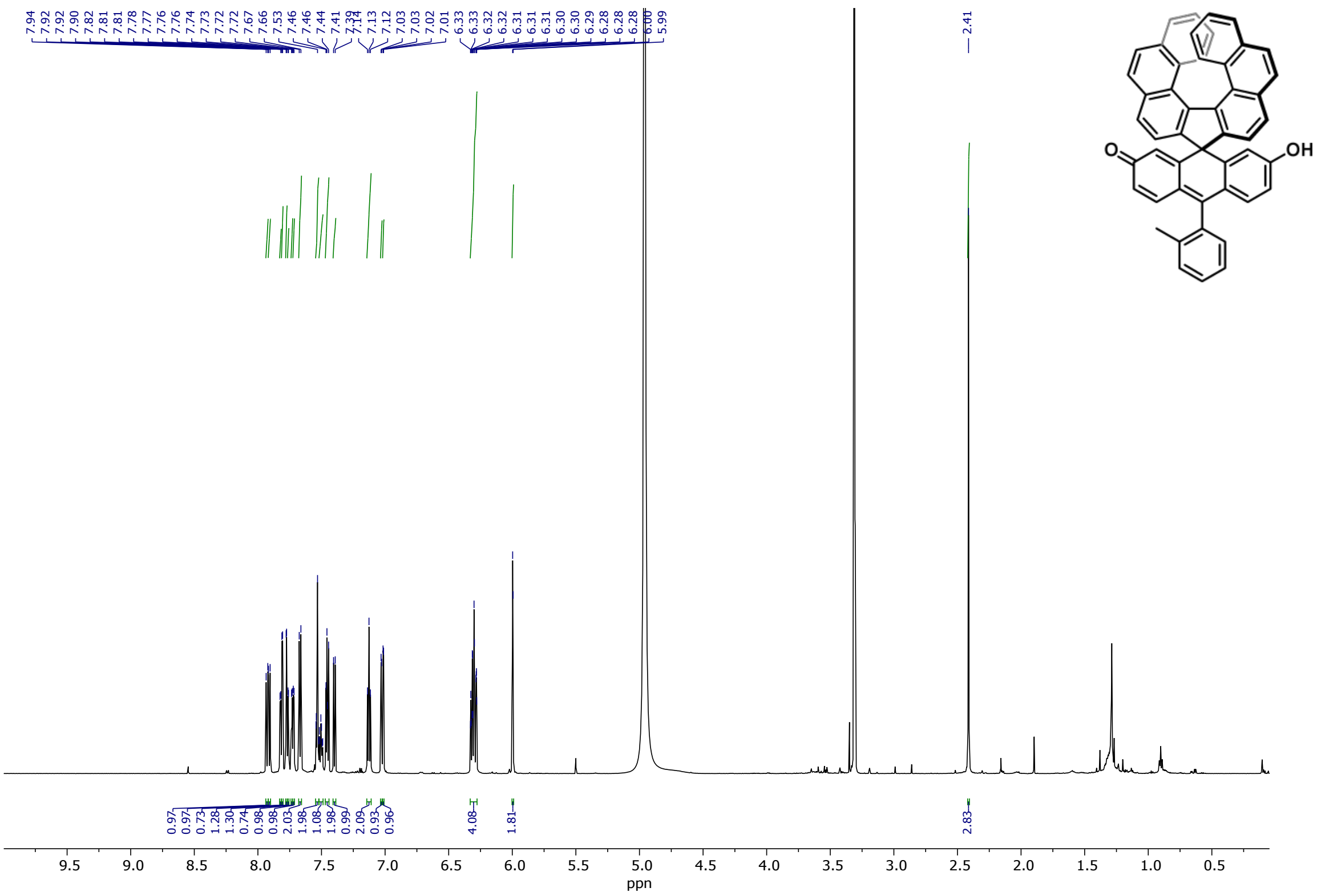

Figure S13. ¹H-NMR (600 MHz, CD_3OD) spectrum of compound *rac*-**7H-CF**.

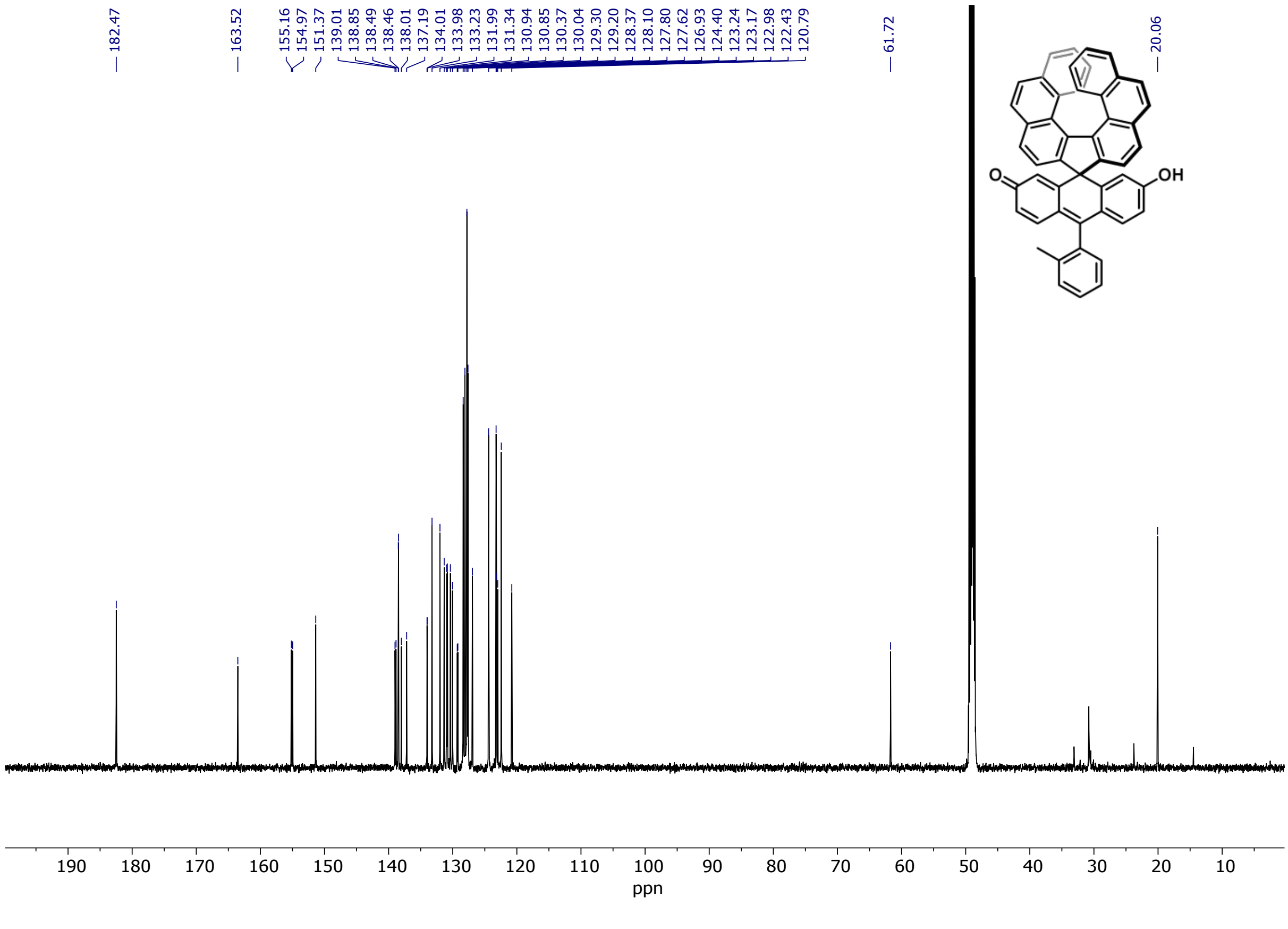

Figure S14. ¹³C-NMR (151 MHz, CD_3OD) spectrum of compound *rac*-**7H-CF**.

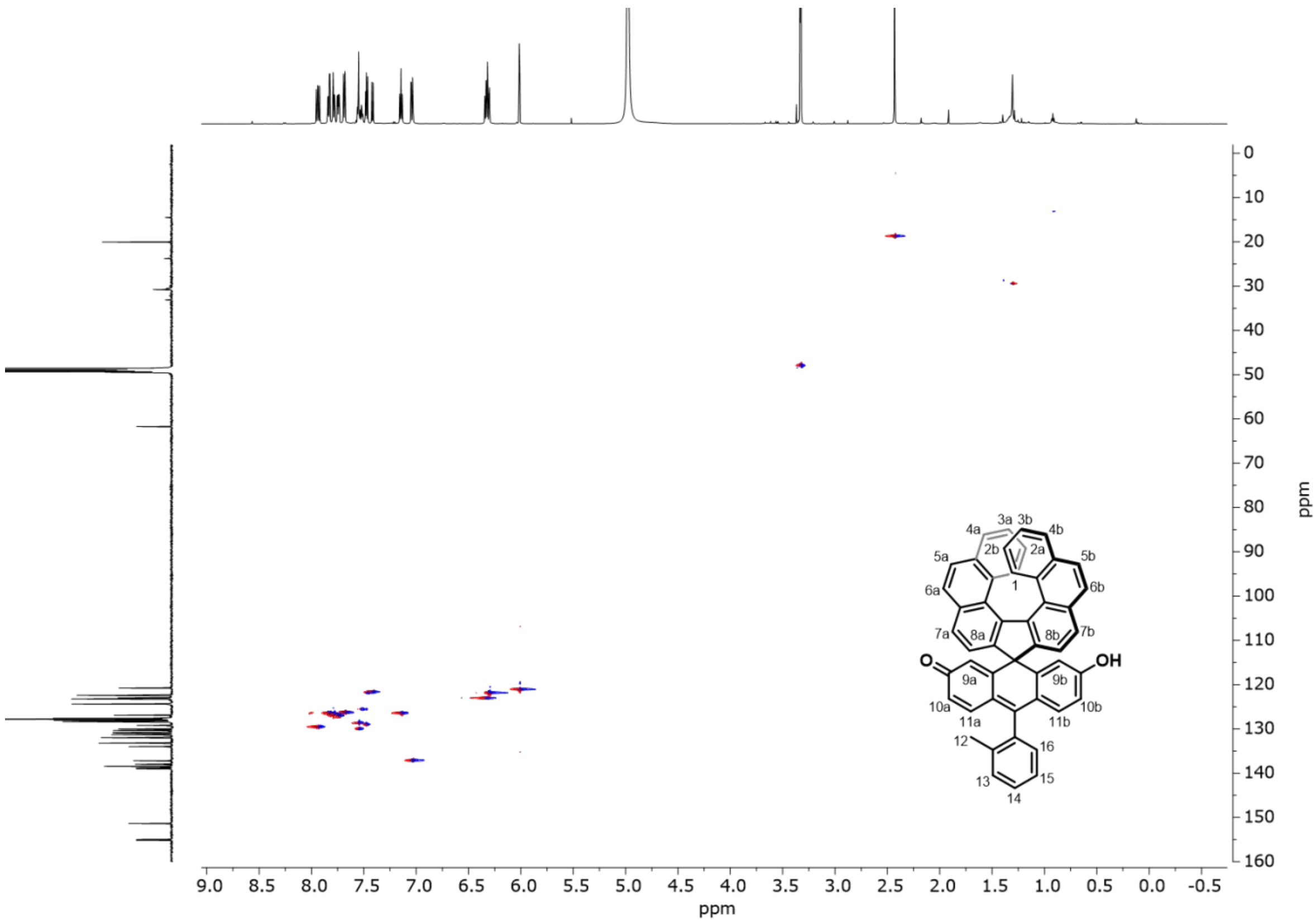

Figure S15. HSQC 2D spectrum of compound *rac*-**7H-CF**.

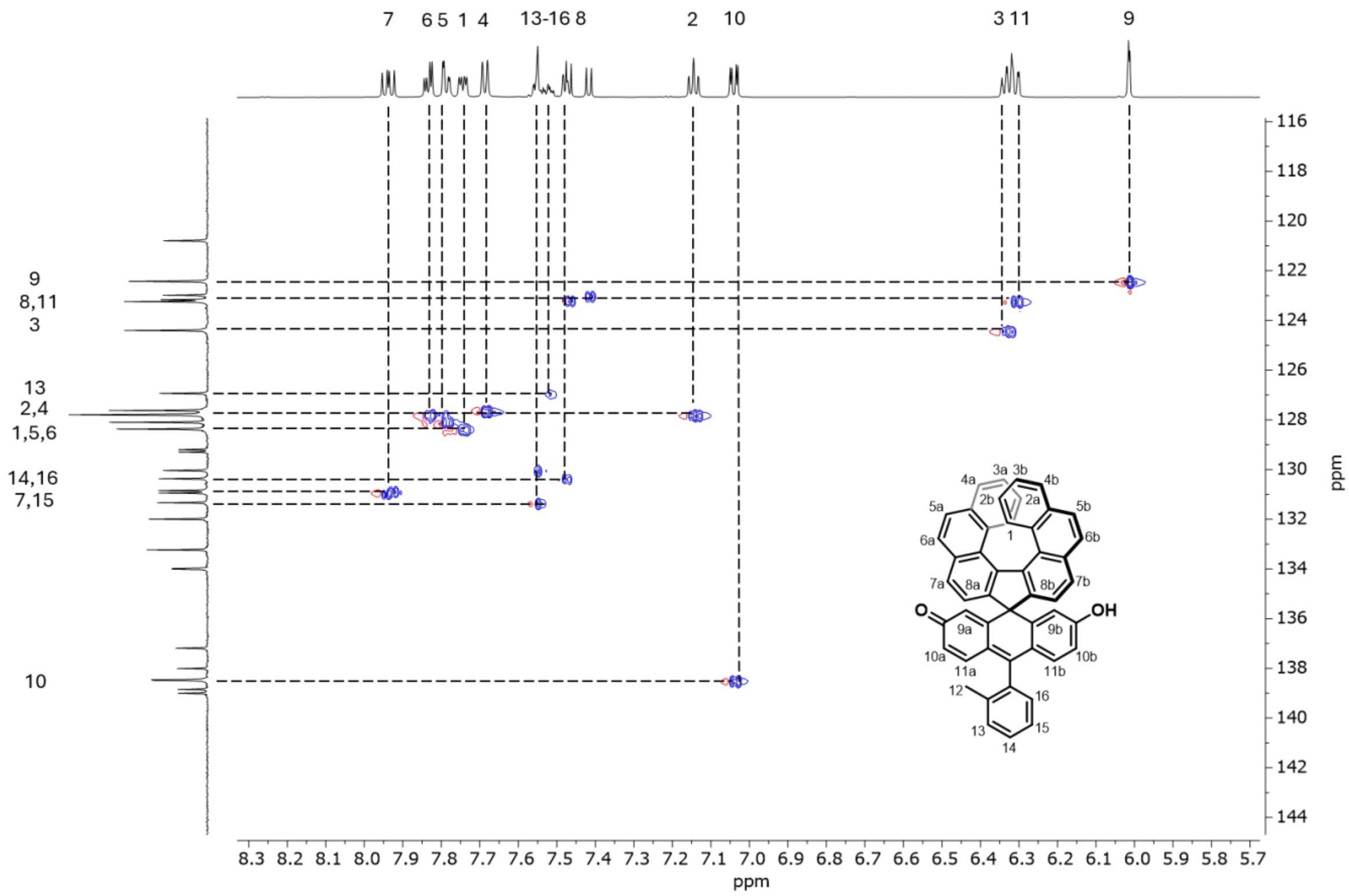

Figure S16. Zoom in and correlations in the 5.5-8.5 ppm region of the HSQC spectrum of *rac*-**7H-CF**.

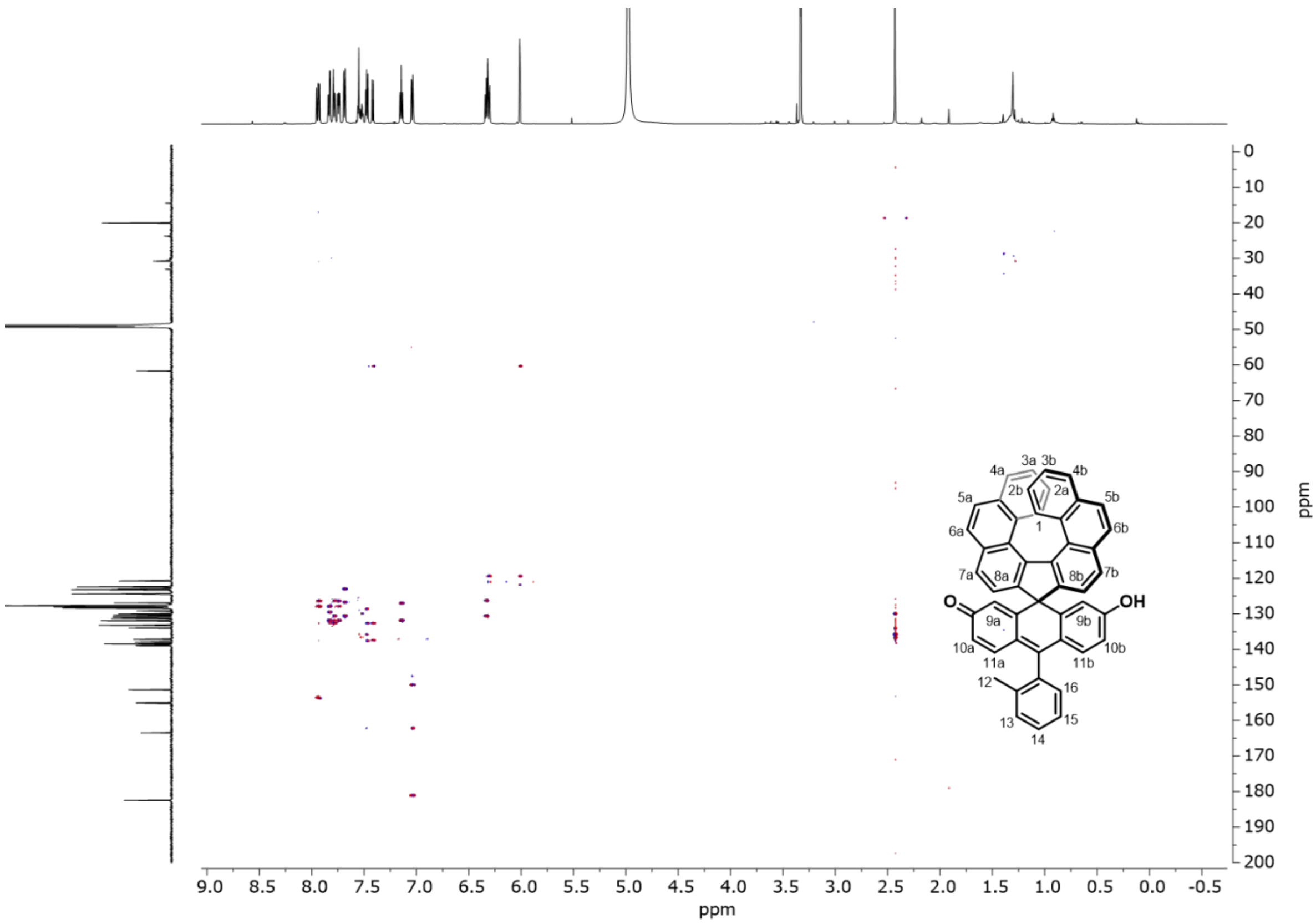

Figure S17. HMBC 2D spectrum of compound *rac*-**7H-CF**.

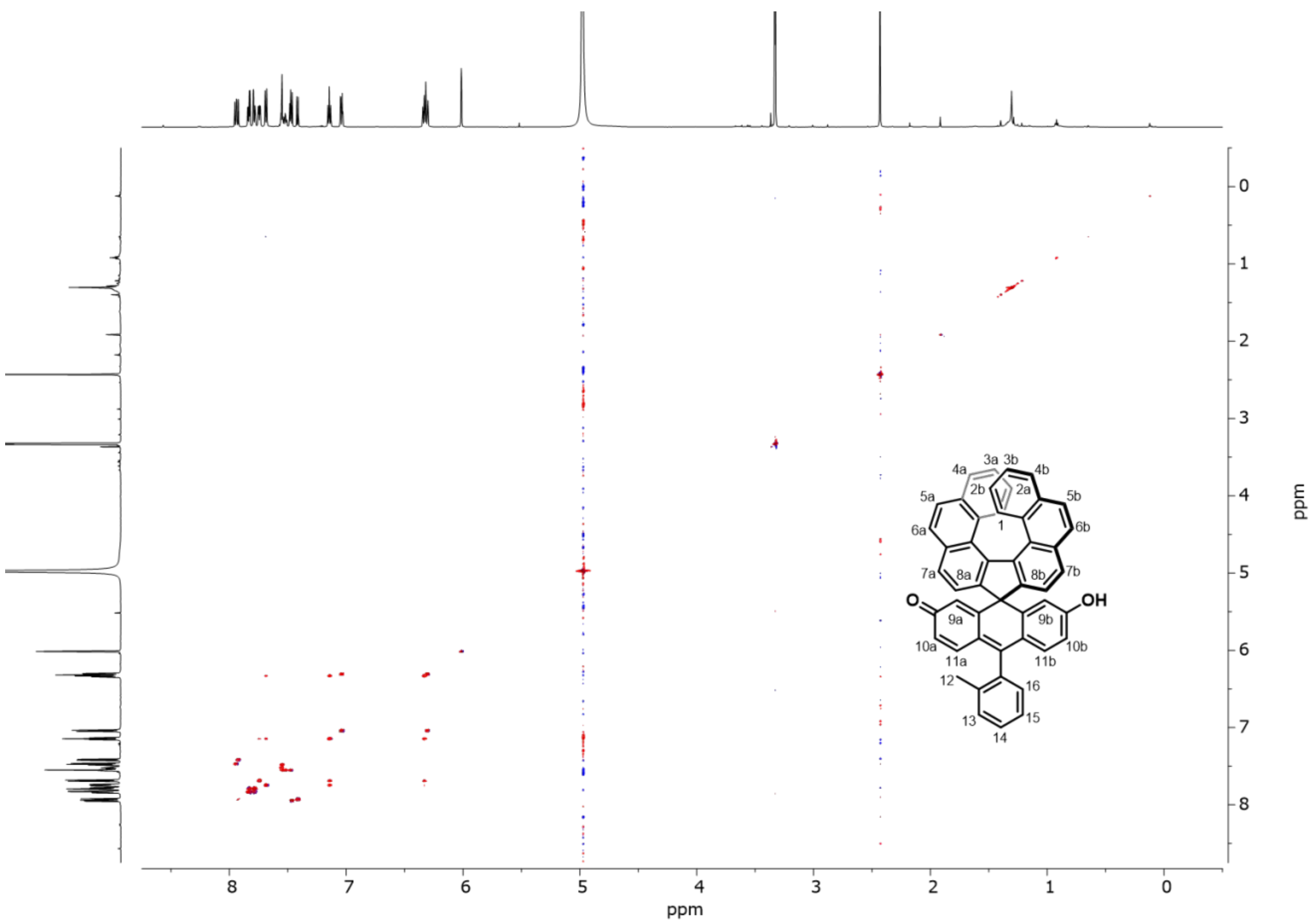

Figure S18. 2D TOCSY spectrum of compound *rac*-**7H-CF**.

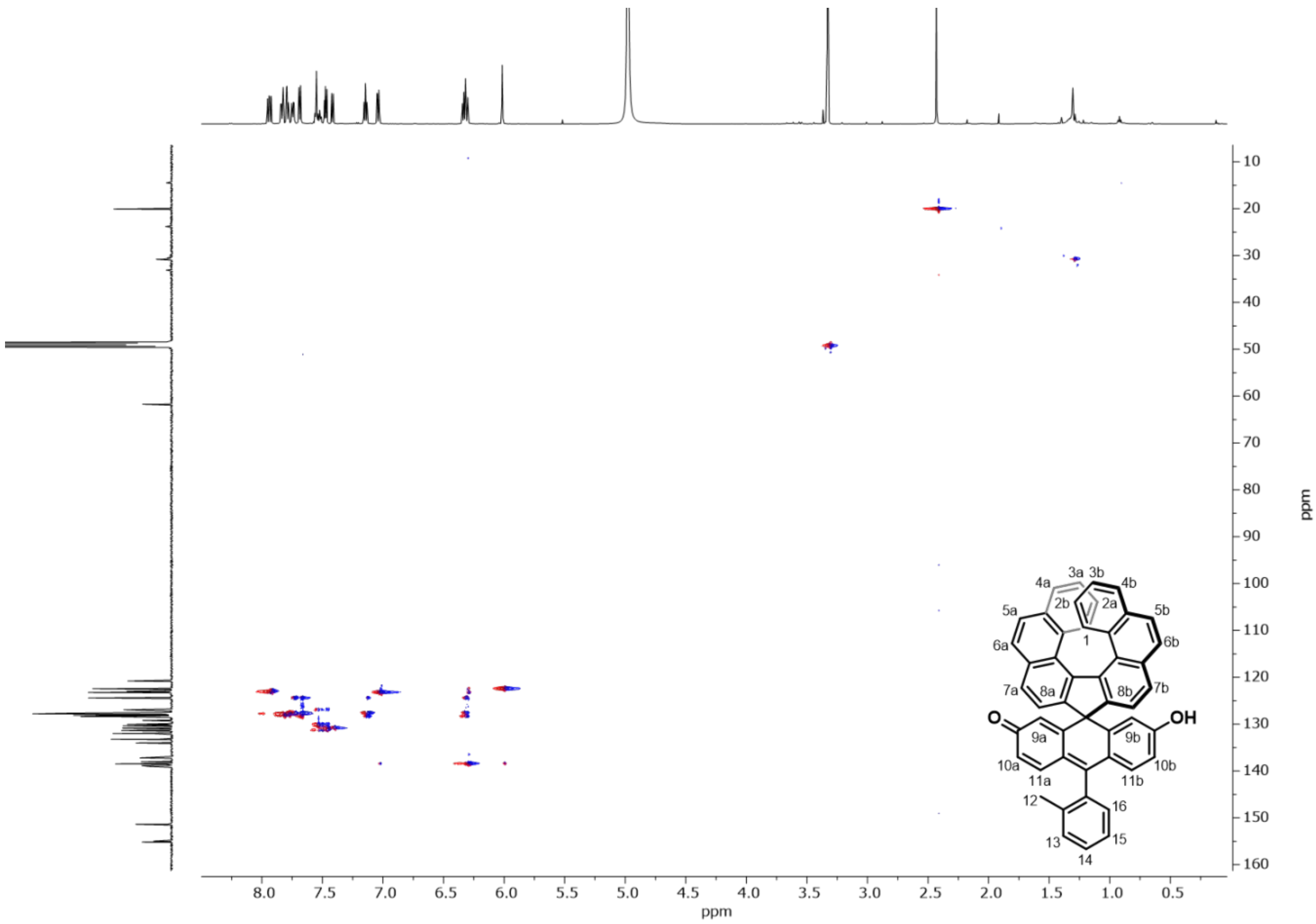

Figure S19. HSQC-TOCSY 2D spectrum of compound *rac*-**7H-CF**.

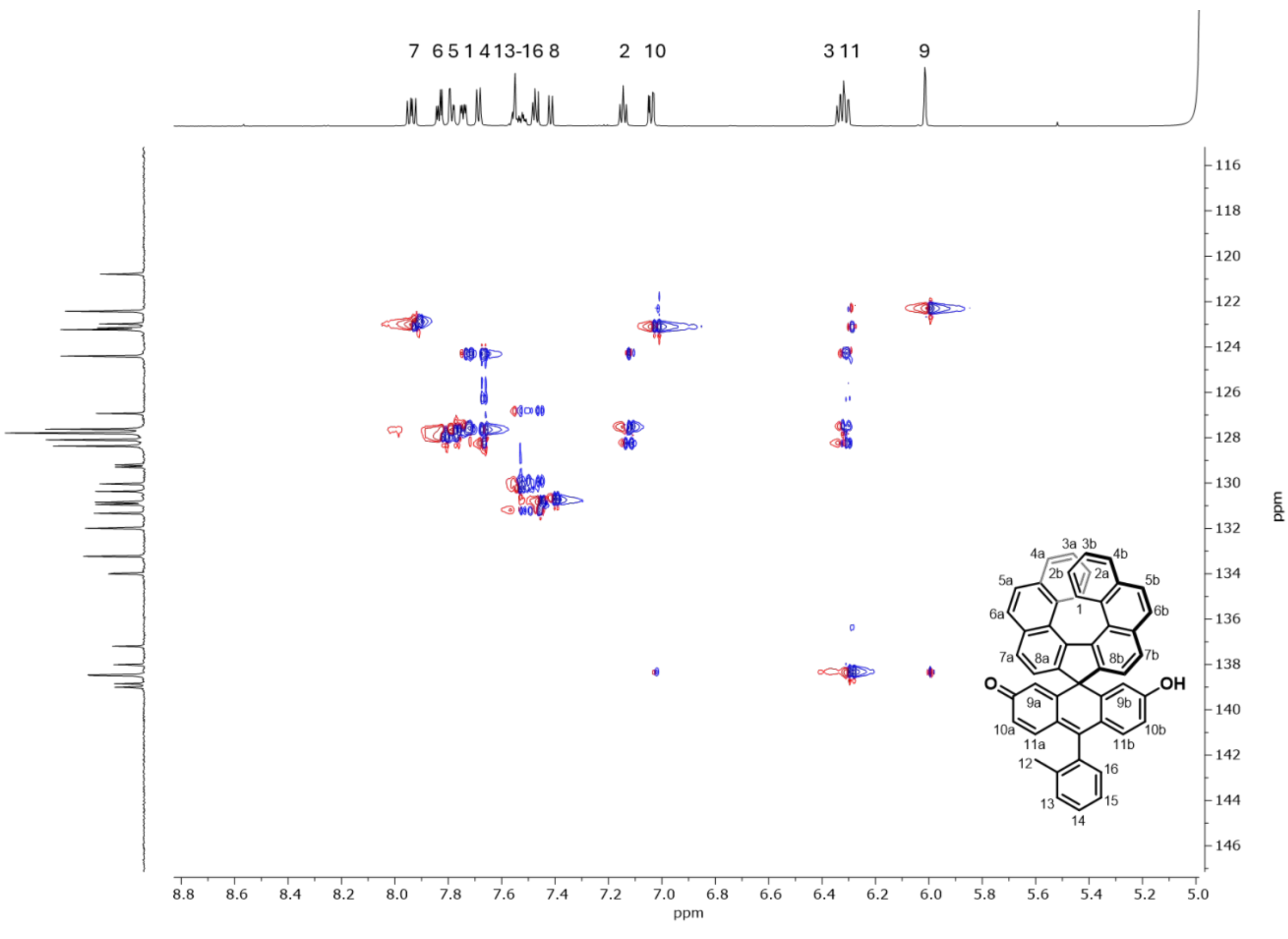

Figure S20. Zoom in the 8.5-5.2 ppm region of HSQC-TOCSY 2D spectrum of compound *rac*-**7H-CF**.

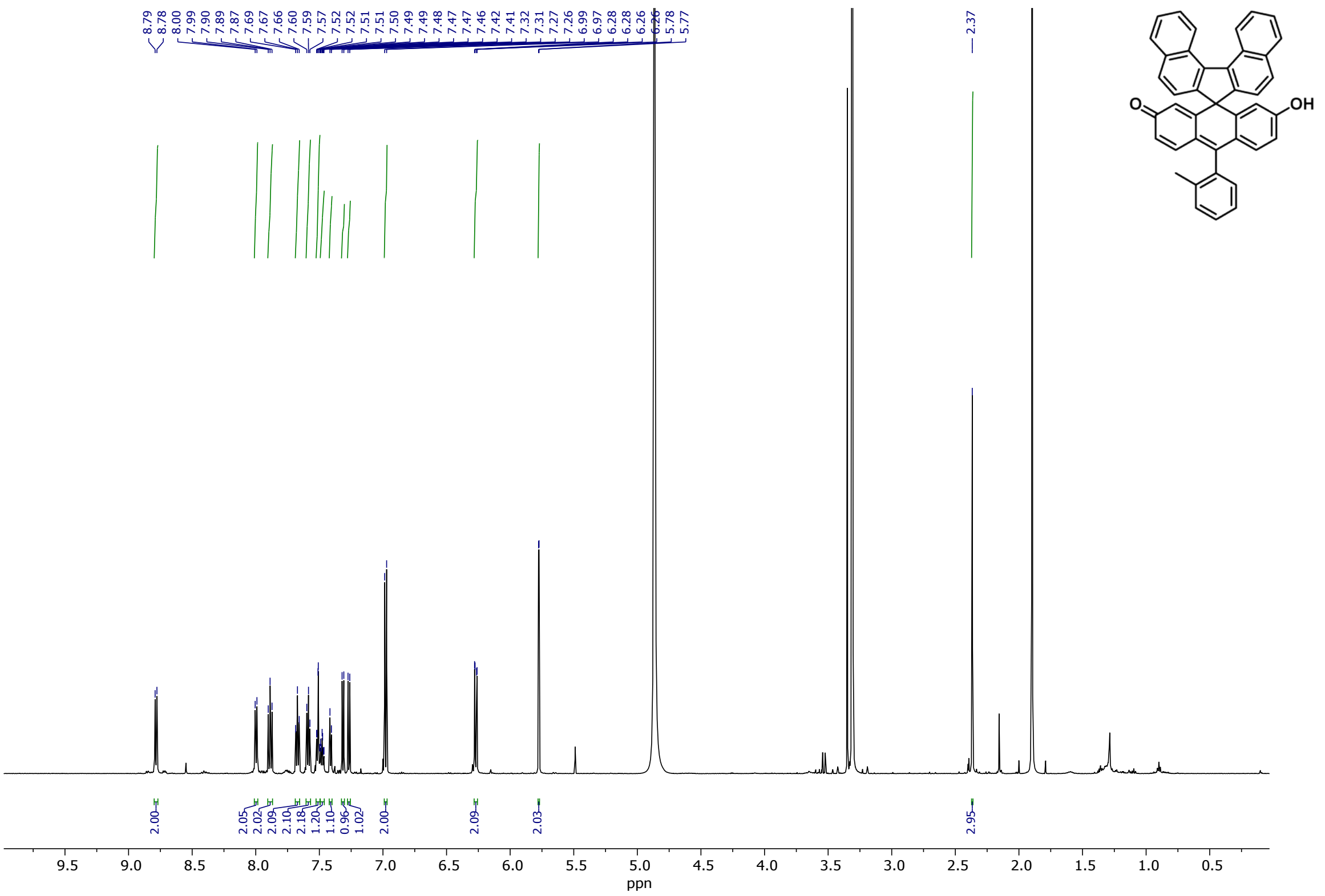

Figure S21. ^{1}H-NMR (600 MHz, CD_3OD) spectrum of compound *rac*-**5H-CF**.

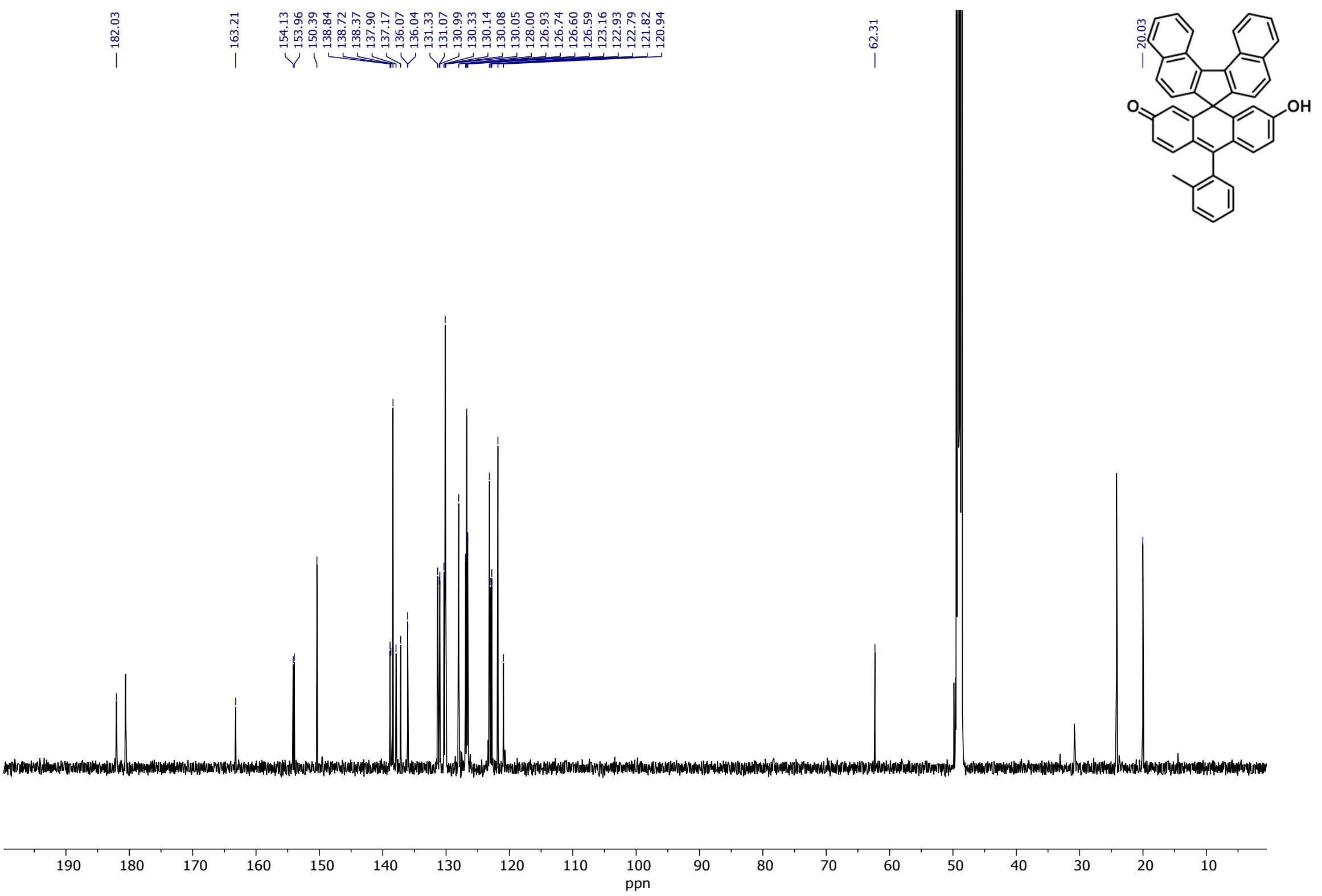

Figure S22. ^{13}C-NMR (151 MHz, CD_3OD) spectrum of compound *rac*-**5H-CF**.

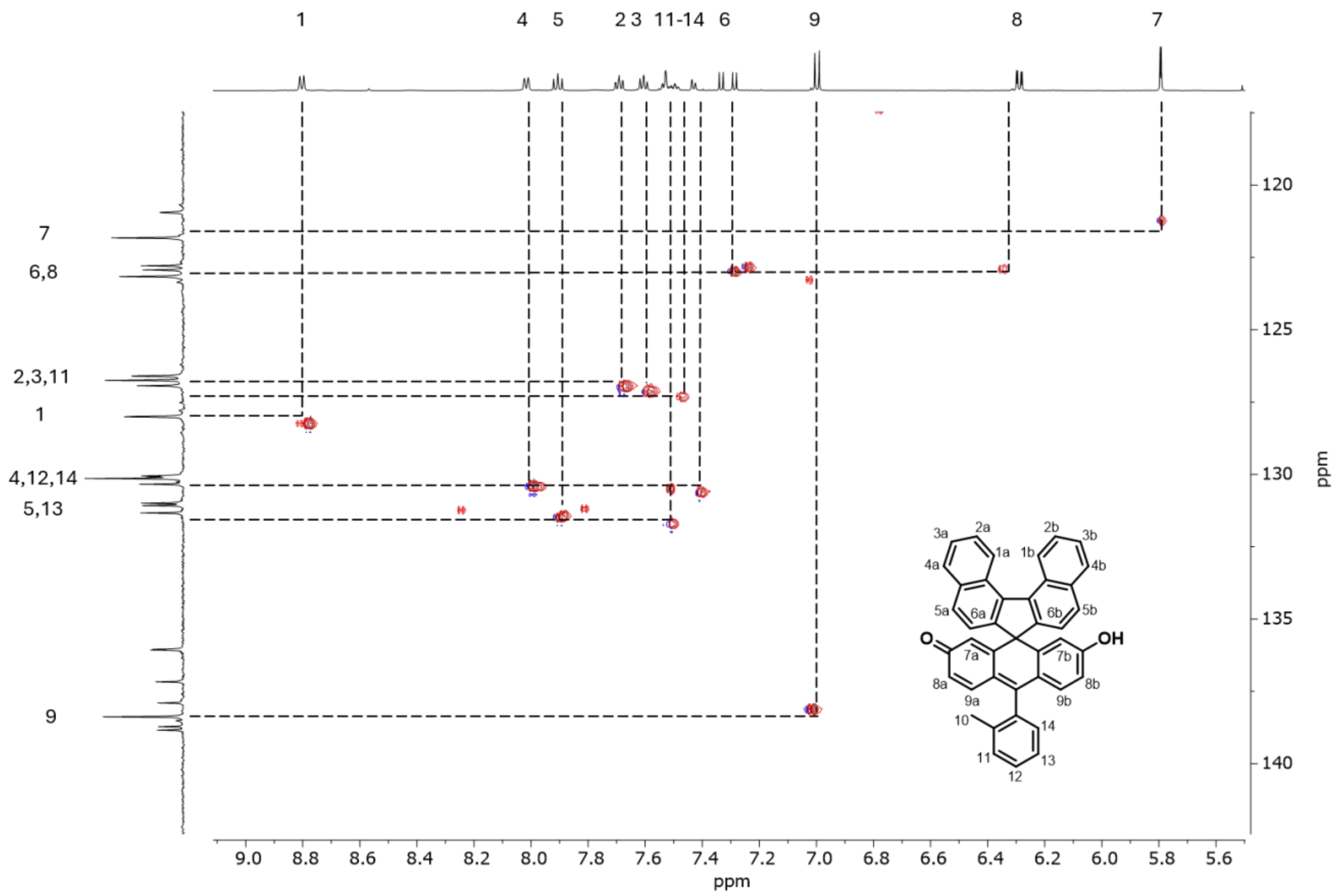

Figure S23. Zoom in the 9-5.6 ppm region of the HSQC spectrum of compound *rac*-**5H-CF**.

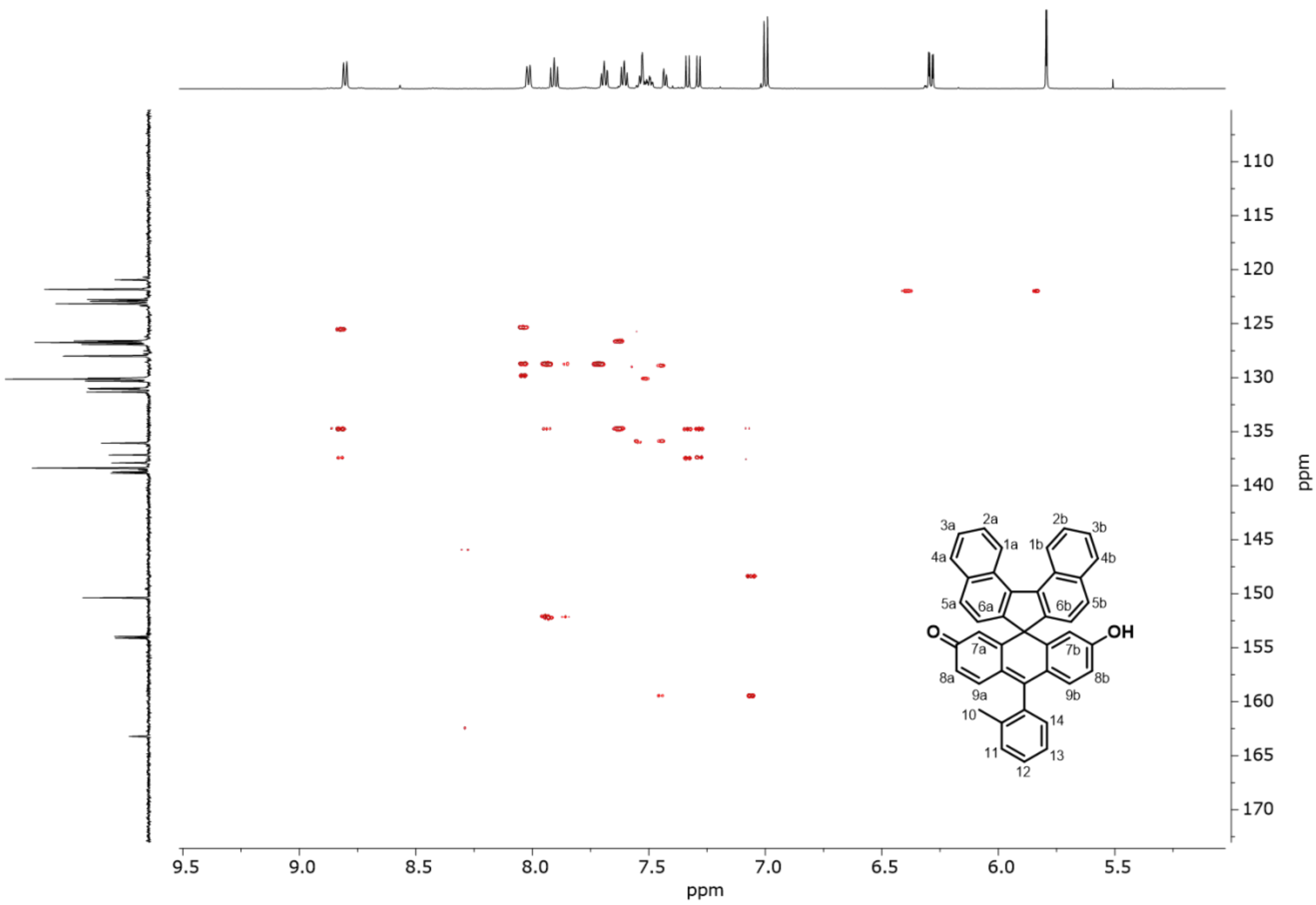

Figure S24. Zoom in the 9-5.5 ppm region of the HMBC spectrum of compound *rac*-**5H-CF**.

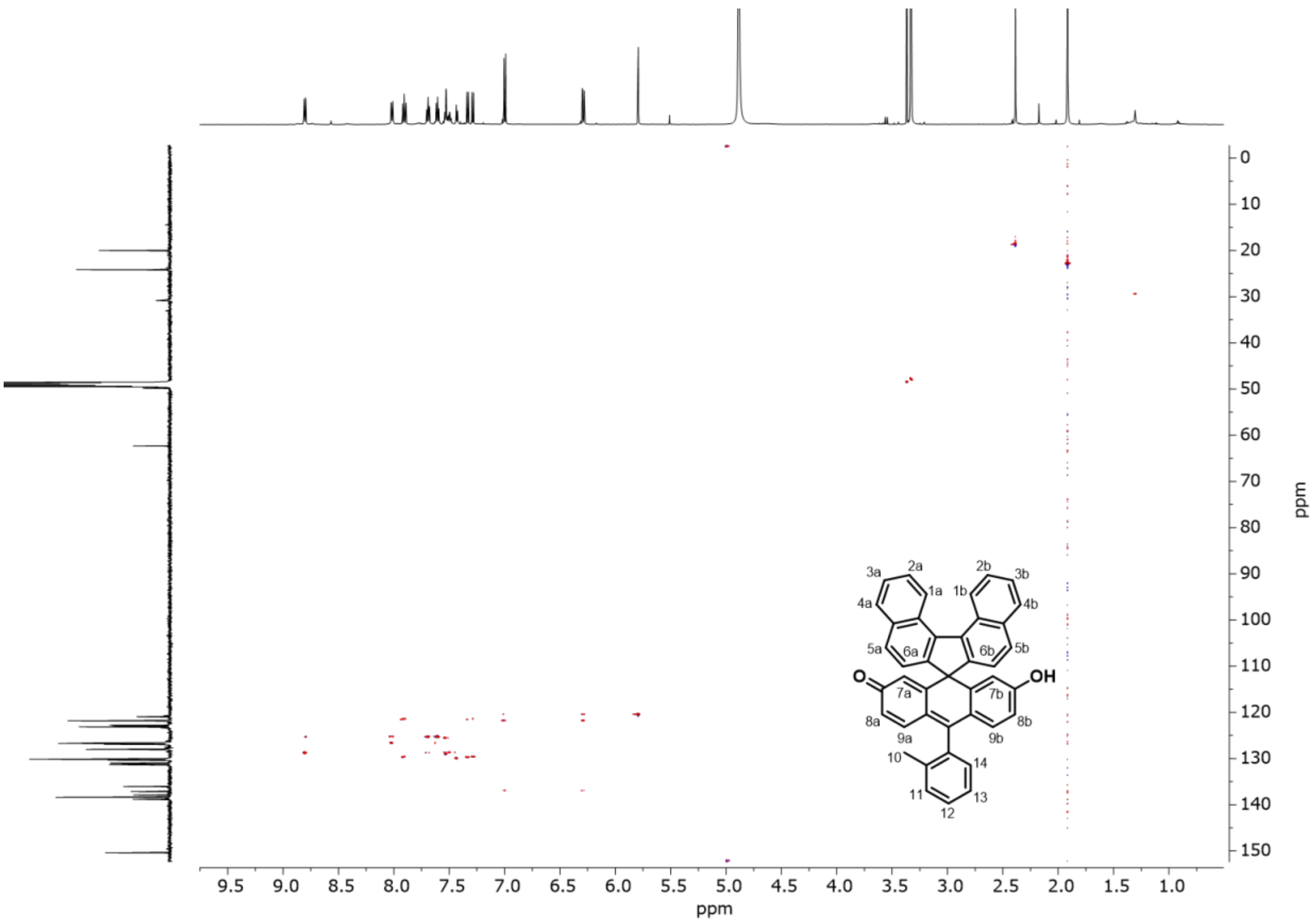

Figure S25. HSQC-TOCSY 2D spectrum of compound *rac*-**5H-CF**.

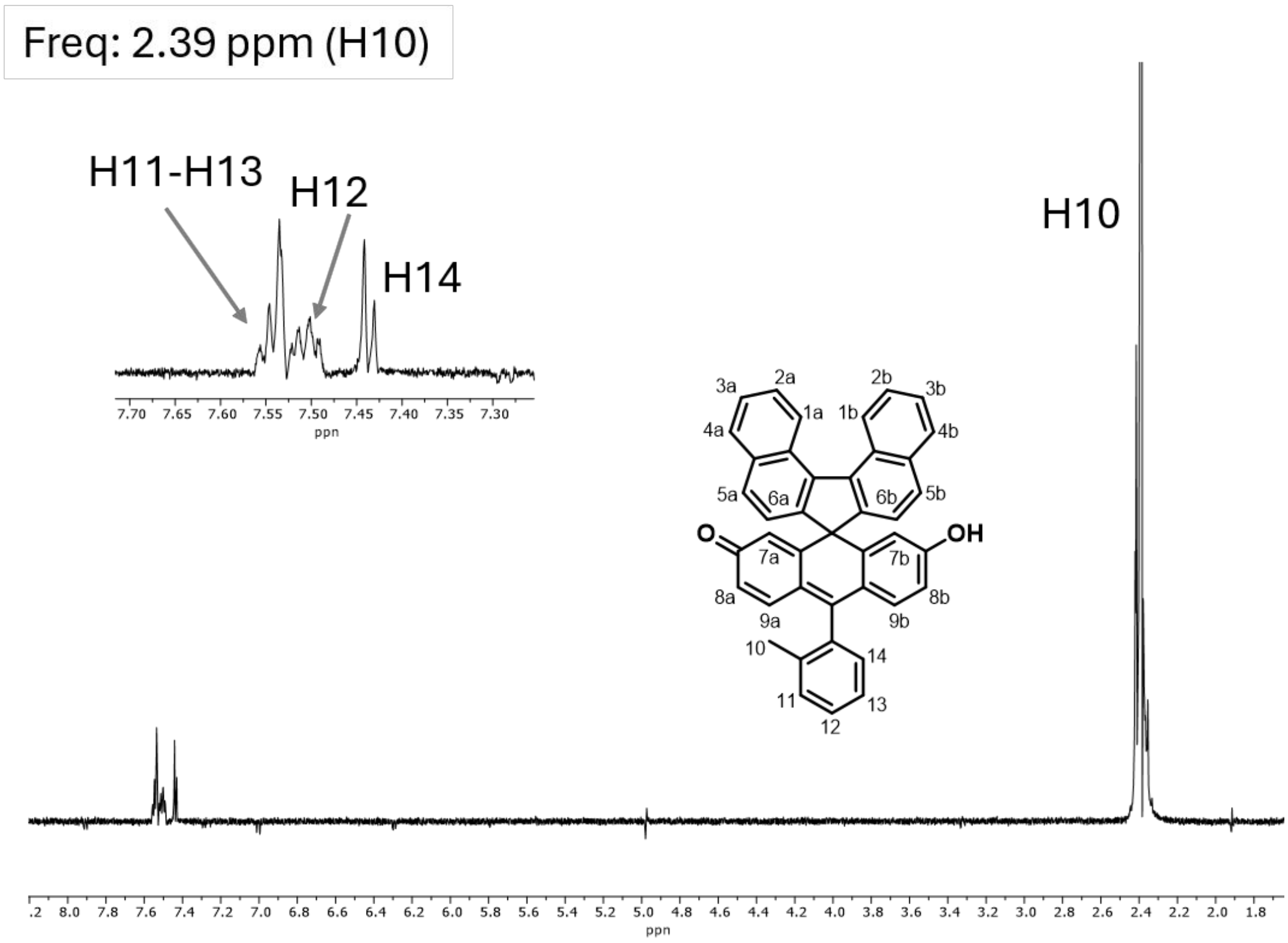

Figure S26. Examples of TOCSY correlations in the o-tolyl fragment for compound *rac*-**5H-CF**.

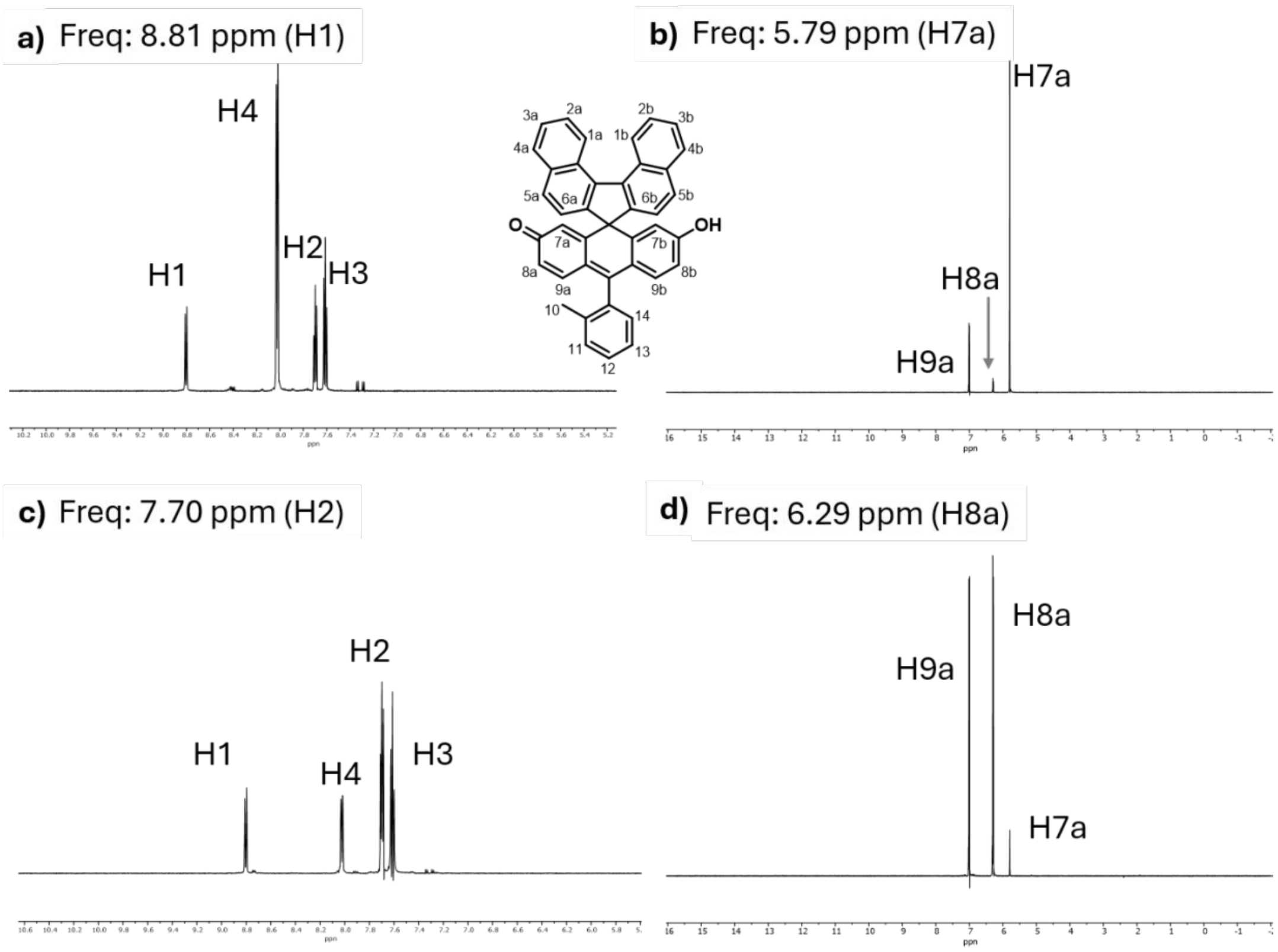

Figure S27. Examples of TOCSY correlations in the helicenoid fragment (a and c) and the central core (b and d) for compound *rac*-**5H-CF**.

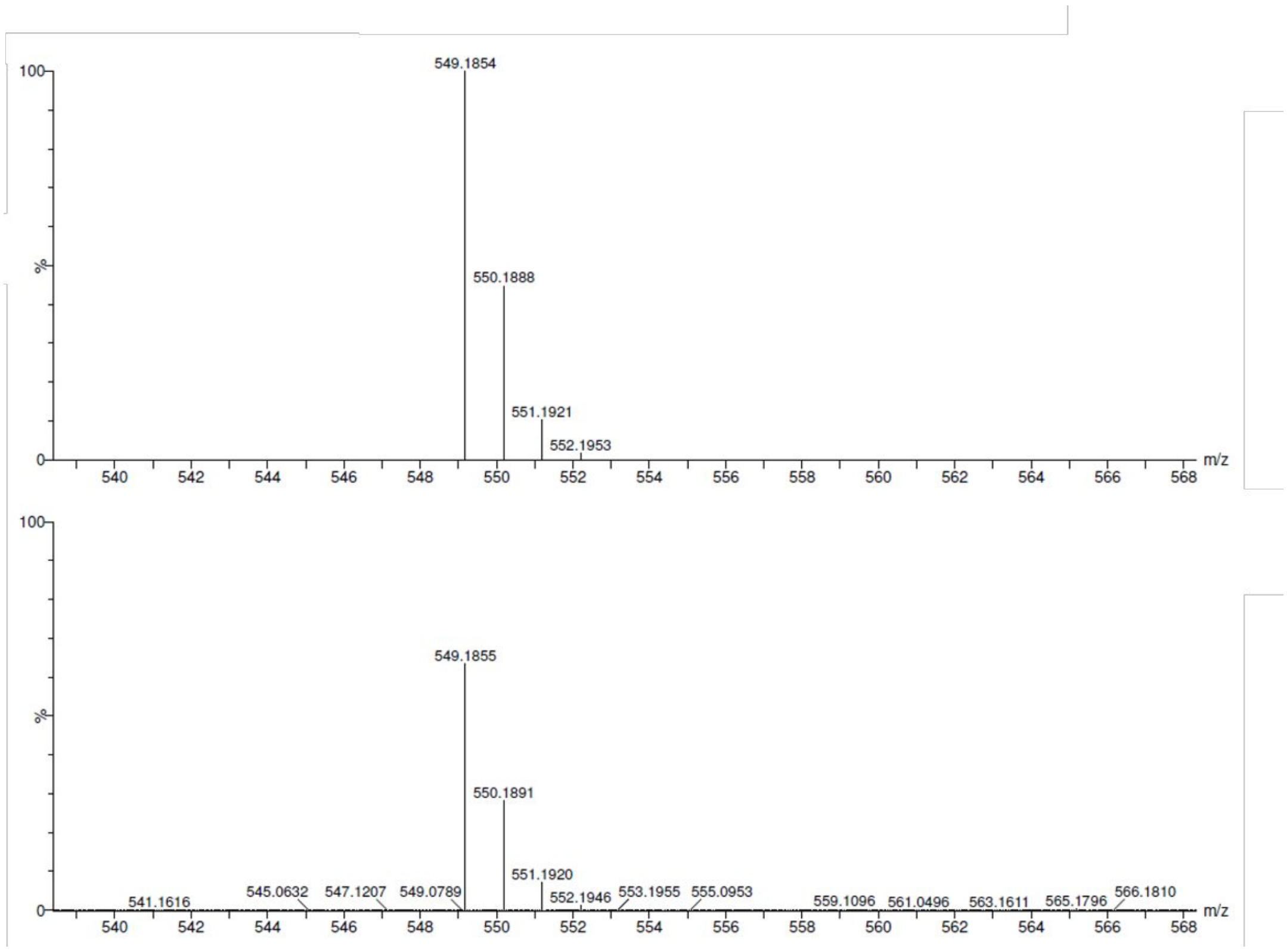

Figure S28. Isotopic distribution of the [M-H]⁺ peak for compound **5H-CF** found by ESI (negative mode). Top: Theoretical. Bottom: Experimental.

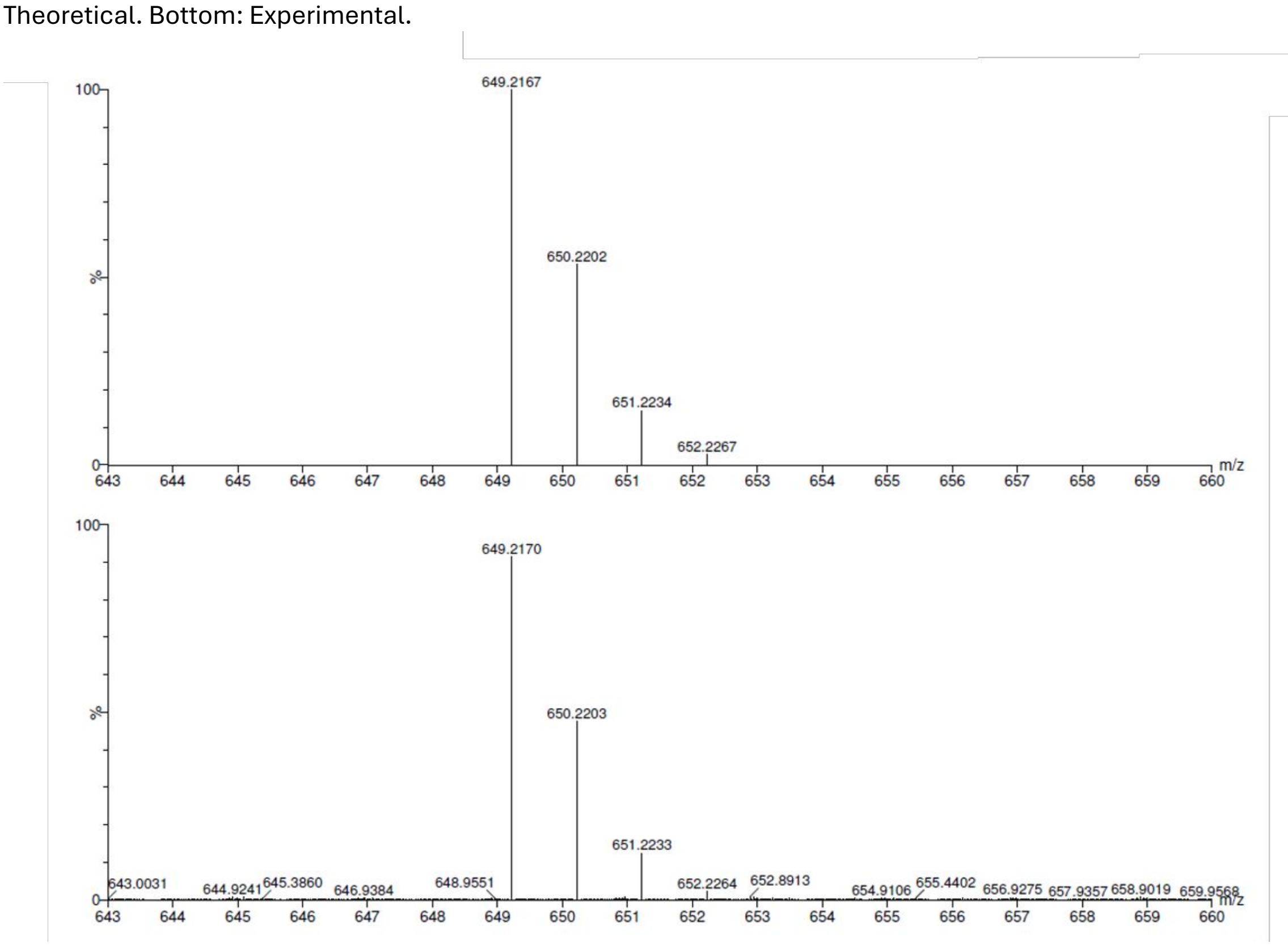

Figure S29. Isotopic distribution of the [M-H]⁺ peak for compound **7H-CF** found by ESI (negative mode). Top: Theoretical. Bottom: Experimental.

2. PHOTOPHYSICAL STUDIES

2.1. General Information

Absorbance was measured using a Cary 60 spectrophotometer. For fluorescence measurements we used a JASCO FP8300 spectrofluorometer and a FluoTime 200 fluorometer (PicoQuant GmbH) for measuring fluorescence decay. A Shimadzu HPLC with a ChiralPak IA column was used for the separation of enantiomers. An Olis DSM172 spectrophotometer was used for the chiroptical characterization (CD and CPL). All measurements were performed in a 1.0 cm path length quartz cuvette, and HPLC grade solvents were used.

2.2. Absorbance and emission in EtOH and DMSO

A 1 mM stock solution of *rac*-**5H-CF** and *rac*-**7H-CF** in ethanol were prepared, from which 1.0 × 10^{-5} M solutions in EtOH and DMSO were analyzed.

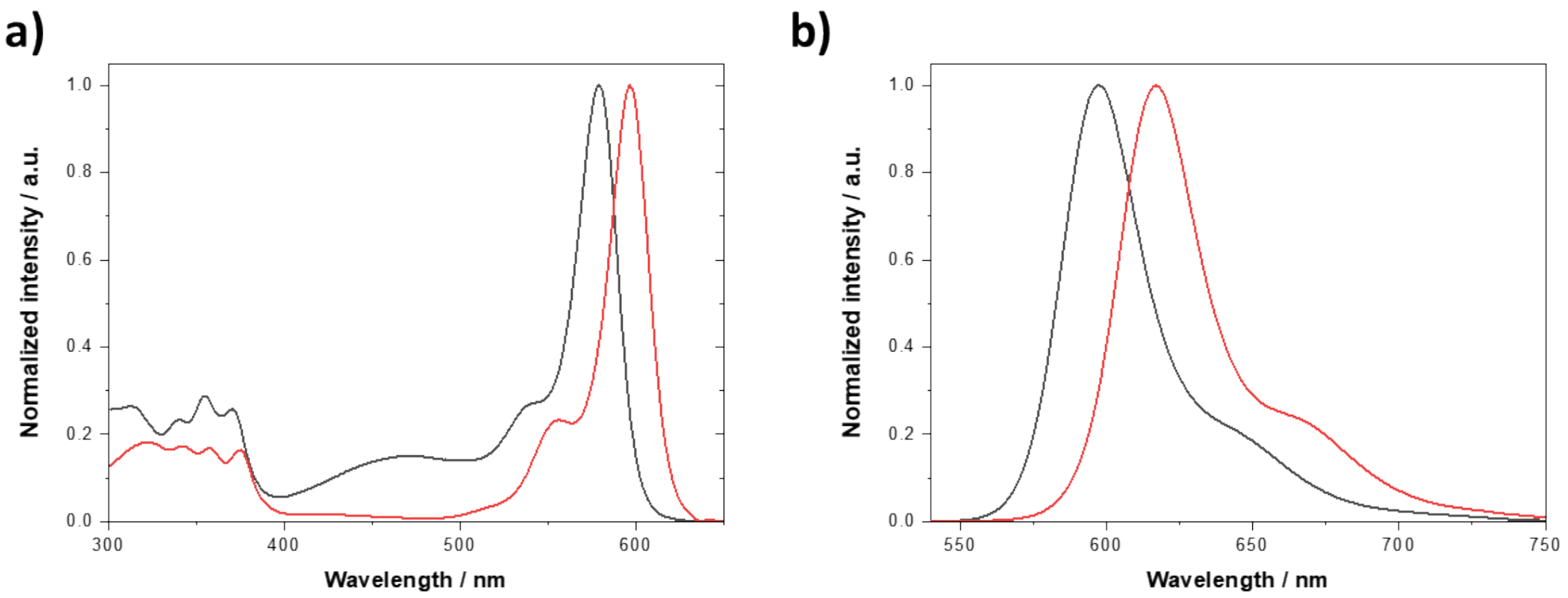

Figure S30. Normalized (a) UV–vis absorption spectra and (b) emission spectra (λ_{exc} = 530 nm) of *rac*-**5H-CF** in EtOH (black) and DMSO (red).

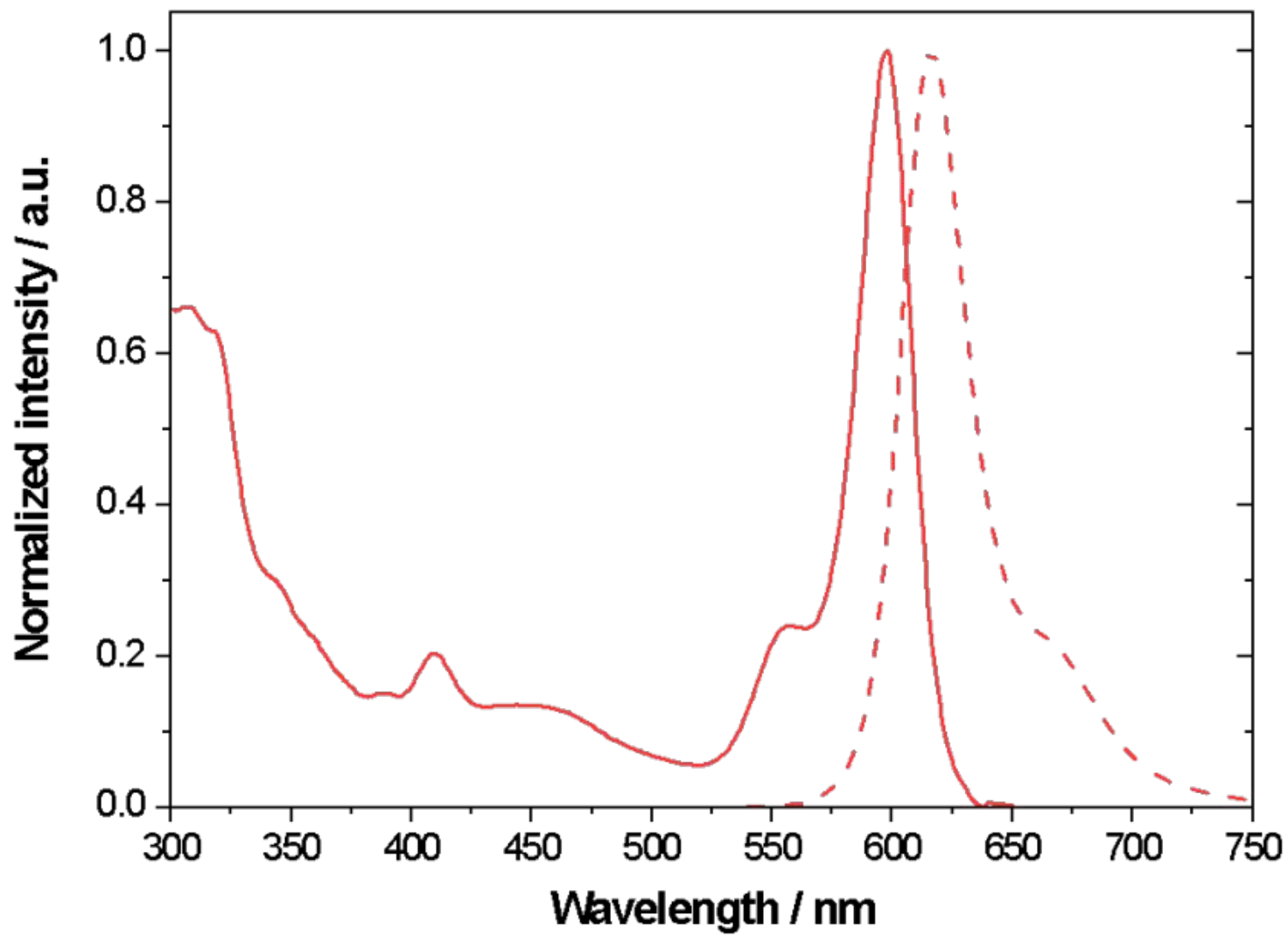

Figure S31. Normalized UV–vis absorption spectra (solid) emission spectra (dashed line, λ_{exc} = 530 nm) of *rac*-**7H-CF** in DMSO.

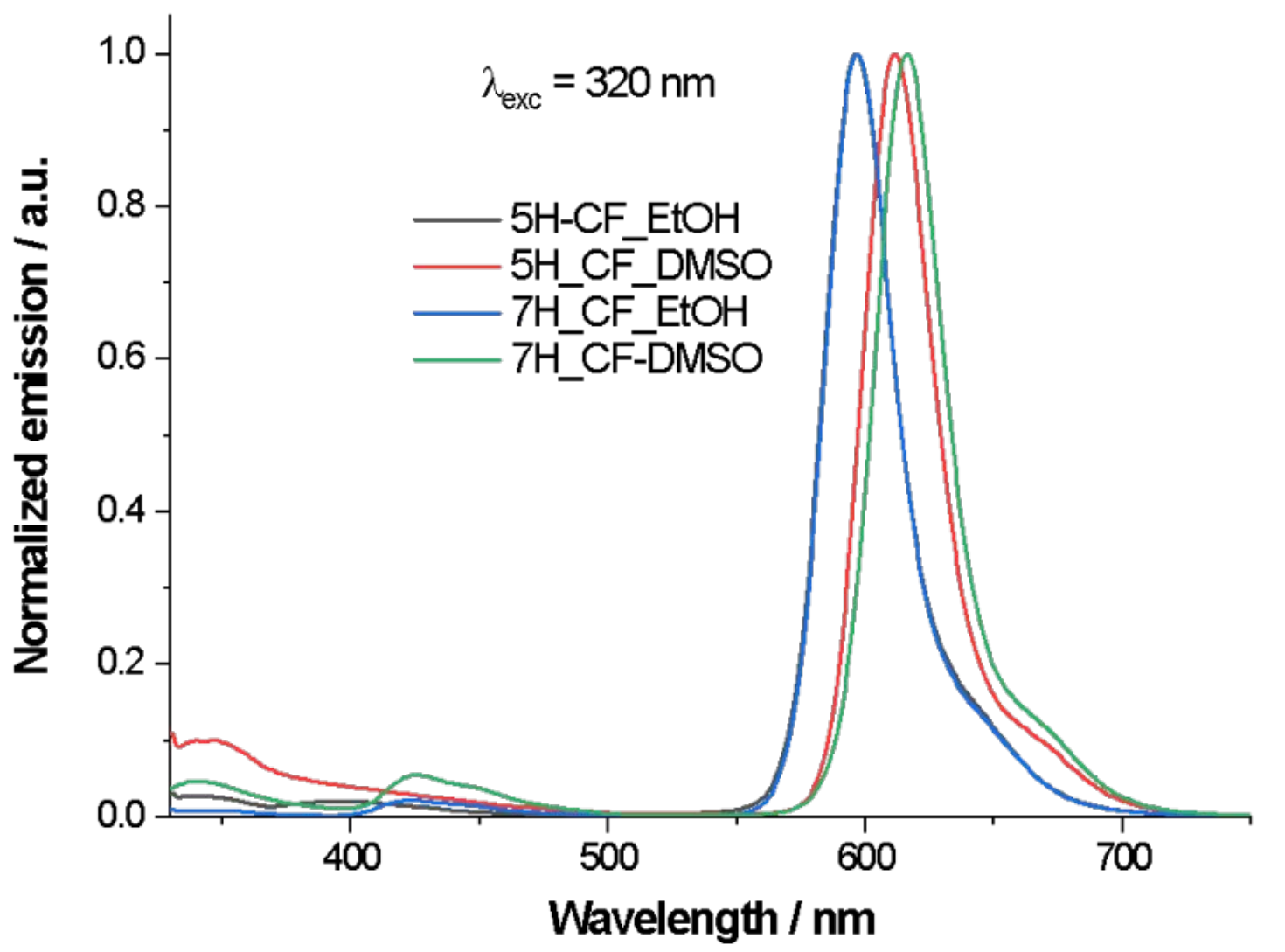

Figure S32. Normalized fluorescence spectra of *rac*-**5H-CF** and *rac*-**7H-CF** in DMSO and EtOH with excitation at 320 nm.

2.3. Determination of molar extinction coefficient (ε)

A stock solution with an absorbance in the range of 1–2 was prepared, from which a series of solutions of increasing concentration were obtained, exhibiting absorbances between 0 and 1.

Measurements were carried out in EtOH and DMSO for both compounds.

The ε value was determined by plotting absorbance intensity as a function of concentration (μM), in accordance with the Beer–Lambert law using Equation 1.

$$A = \varepsilon\, b\, c \tag{1}$$

where A denotes the measured absorbance, ε refers to the molar extinction coefficient, b the optical path length (1.0 cm), and c the concentration of the solution.

For measurements of *rac*-**5H-CF** in DMSO, a stock solution of 3.4 × 10^{-4} M was prepared, affording solutions of 12, 57, 115, 172, 229, and 287 μM. For measurements in EtOH, a stock solution of 2.3 × 10^{-4} M was prepared, affording solutions of 9, 44, 88, 131, 175, and 219 μM.

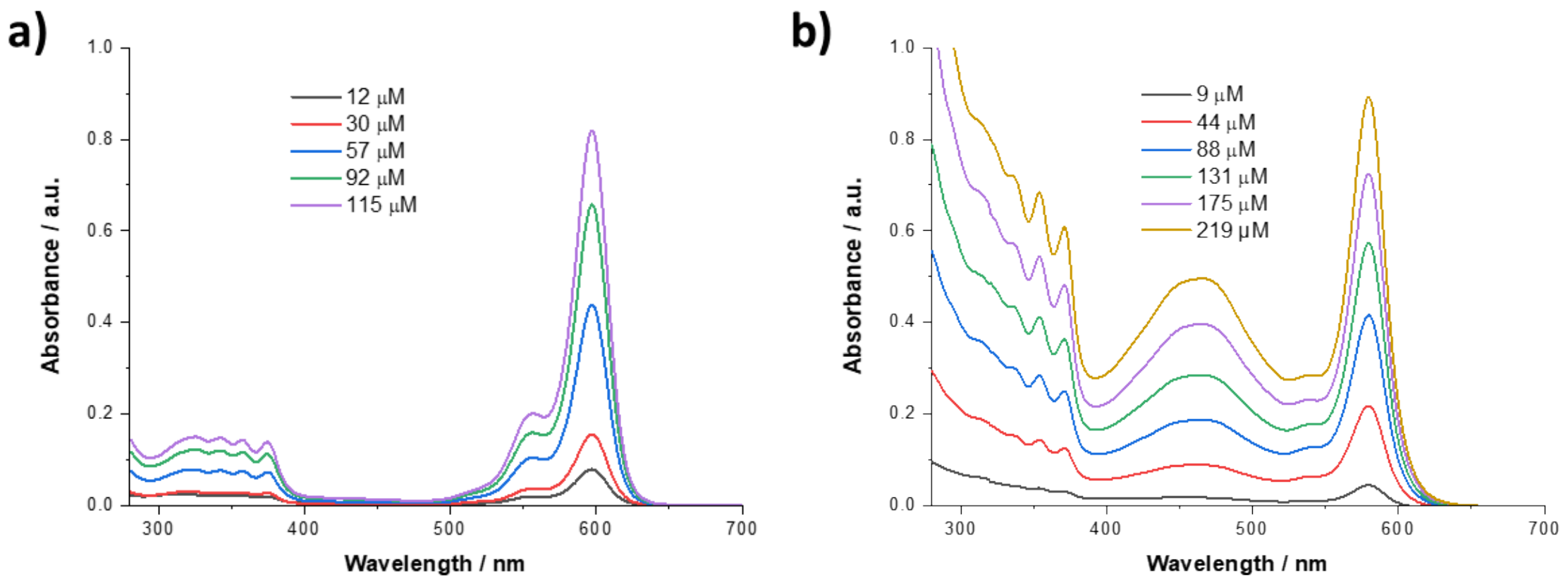

Figure S33. Absorbance of *rac*-**5H-CF** at varying concentrations in (a) DMSO and (b) EtOH.

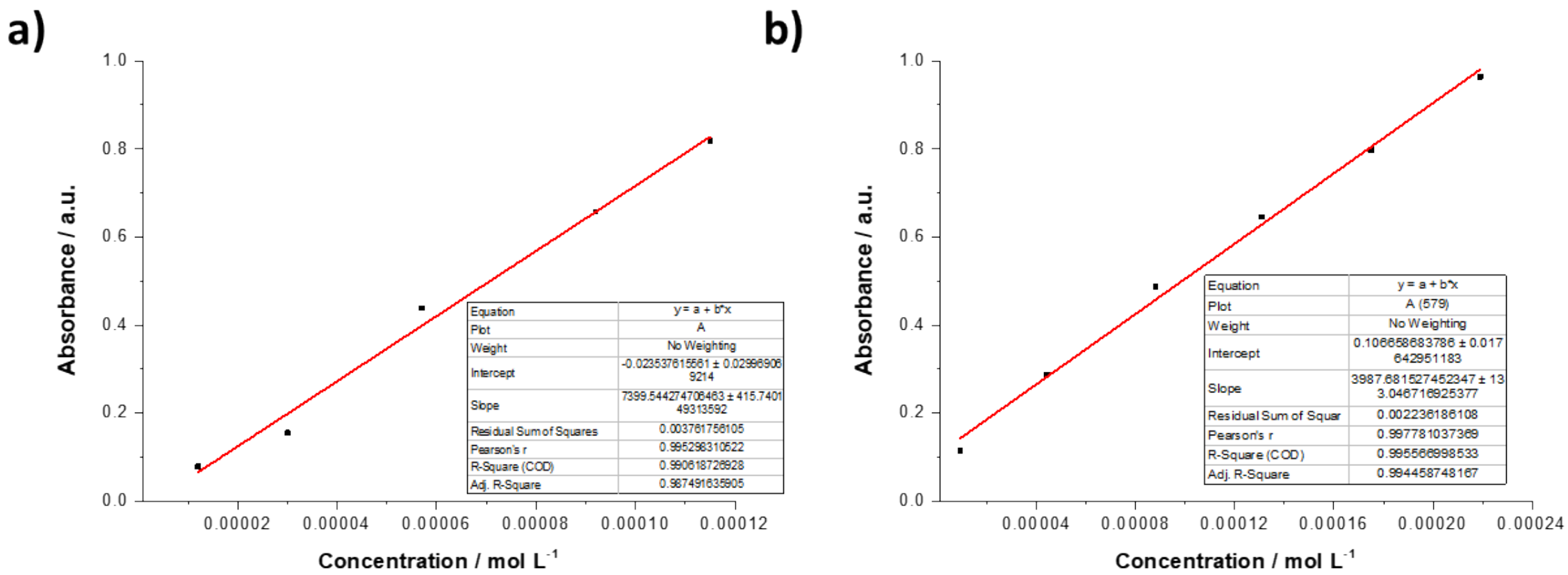

Figure S34. Absorbance of *rac*-**5H-CF** as a function of concentration in a) DMSO at 597 nm, showing ε = 7.40 x 10^3 $M^{-1}\cdot cm^{-1}$; and b) EtOH at 579 nm, showing ε = 3.99 x 10^3 $M^{-1}\cdot cm^{-1}$.

For measurements of *rac*-**7H-CF** in DMSO, a stock solution of 1.3 × 10^{-4} M was prepared, affording solutions of 0.62, 1.56, 3.13, 4.69, 6.25, and 7.81 μM. For measurements in EtOH, a stock solution of 4.0 × 10^{-5} M was prepared, affording solutions of 1, 3, 7, 10, 13, 17 and, 20 μM.

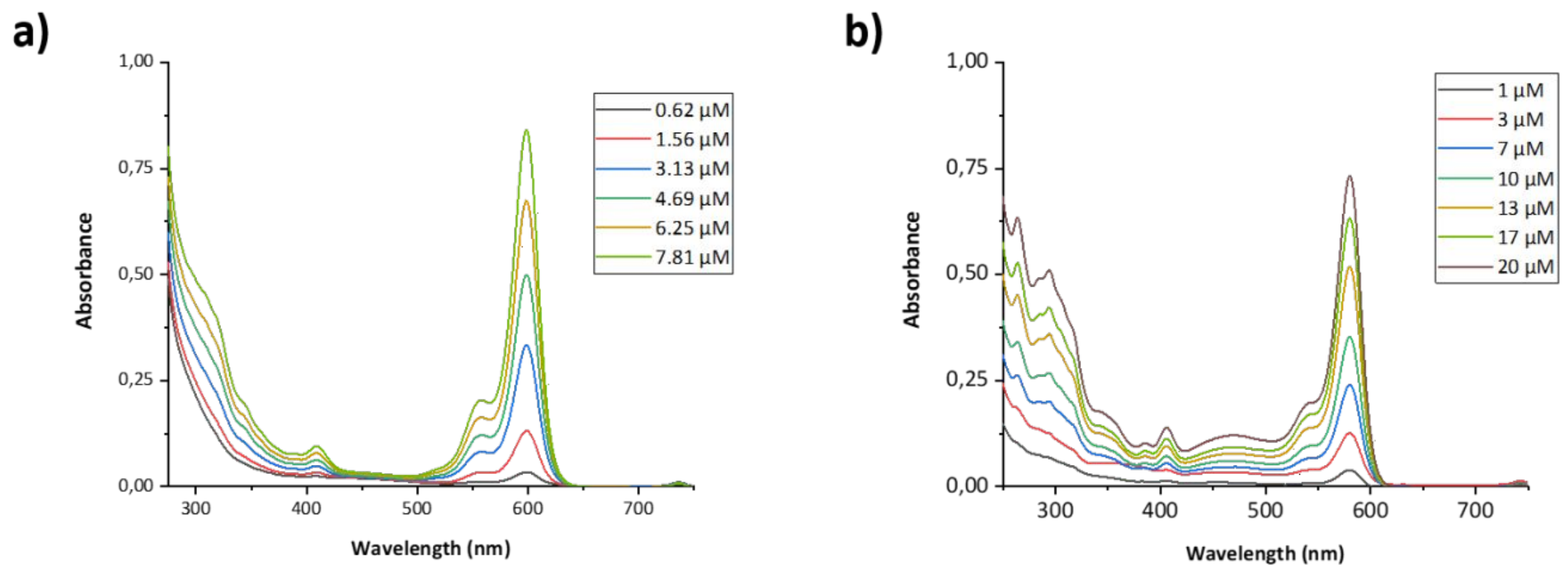

Figure S35. Absorbance of *rac*-**7H-CF** at varying concentrations in (a) DMSO and (b) EtOH.

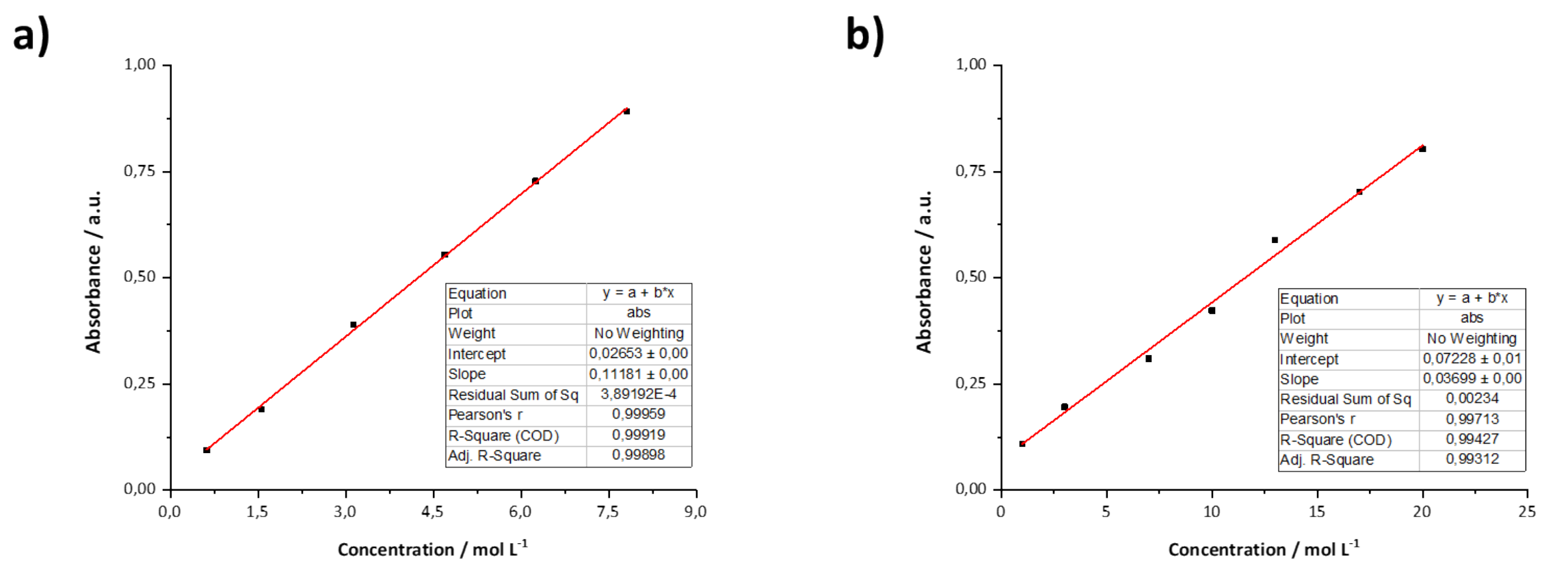

Figure S36. Absorbance of *rac*-**7H-CF** as a function of concentration in a) DMSO at 600 nm, showing ε = 1.12 x 10^5 $M^{-1}\cdot cm^{-1}$; and b) EtOH at 580 nm, showing ε = 3.69 x 10^4 $M^{-1}\cdot cm^{-1}$.

2.4. Quantum Yield and Lifetime

Quantum yields (Φ) of *rac*-**5H-CF** and *rac*-**7H-CF** in different solvents were determined by measuring both absorbance and fluorescence using Rhodamine 101 (Φ_r = 1.0) as the standard.[S3]

Relative fluorescence quantum yields were determined using Equation 2.

$$\Phi_x = \Phi_r \; x \; \frac{F_x}{F_r} \; x \; \frac{1 - 10^{-A_r(\lambda_{ex})}}{1 - 10^{-A_x(\lambda_{ex})}} \; x \; \frac{n_x^2}{n_r^2}$$

(2)

The subscripts x and r refer to the sample and the reference fluorophore with a known quantum yield Φ_r in a specific solvent, respectively. F stands for the spectrally corrected and integrated fluorescence intensity, $A\ (\lambda_{ex})$ denotes the absorbance at the excitation wavelength used, and n corresponds to the refractive index of the solvent.

Time-resolved fluorescence decay traces were collected via the time-correlated single photon counting (TCSPC) method using a FluoTime 200 fluorometer (PicoQuant GmbH). The excitation source was a 530nm laser using a 20 MHz excitation frequency. The full width at half maximum (fwhm) of the laser pulses was around 40 ps. The fluorescence emission was collected at a 90° geometry, focused on the detector after crossing through a polarizer (set at the magic angle), 2 mm slits, and a 2 nm bandwidth monochromator. TCSPC was achieved by a TimeHarp200 board, set at 36 ps/channel. Fluorescence decay traces were collected for the necessary time to reach 20000 counts at the peak channel. For the analyzed compounds, decay traces were collected at three different wavelengths within ± 5nm (Δλ = - 5, 0, +5 nm) of the maximum of the main emission peak.

Table S1. Photoluminescence properties of *rac*-**5H-CF** in EtOH and DMSO.

**SOLVENT**	**λ_{max} abs**	**λ_{max} em (λ_{exc} = 530 nm)**	**ϕ_{FL}**	**τ/ns**
**EtOH**	579	597	0.65±0.06	3.8404±0.0014
**DMSO**	597	616	0.95±0.02	4.8573±0.0048

Table S2. Photoluminescence properties of *rac*-**7H-CF** in EtOH and DMSO.

**SOLVENT**	**λ_{max} abs**	**λ_{max} em (λ_{exc} = 530 nm)**	**ϕ_{FL}**	**τ/ns**
**EtOH**	580	595	0.74±0.06	4.1624±0.0025
**DMSO**	600	613	0.70±0.03	5.0359±0.0041

2.5. Effect of pH

A 1 mM stock solution in DMSO was prepared and subsequently diluted to 1.0×10^{-5} M in phosphate buffer solutions. The buffers were prepared using an acidic phosphate salt (NaH_2PO_4) and a basic phosphate salt (Na_2HPO_4), combined in appropriate ratios to obtain a pH range of approximately 5–10. For *rac*-**5H-CF**, the pH values were 6.22, 6.77, 7.35, 7.88, 8.63, and 9.52. For *rac*-**7H-CF**, the pH values were 5.42, 6.41, 6.86, 7.38, 7.90, 8.14, 8.45, 8.54, 8.92, 9.42, 9.79, and 10.13.

2.5.1. Absorbance and Fluorescence

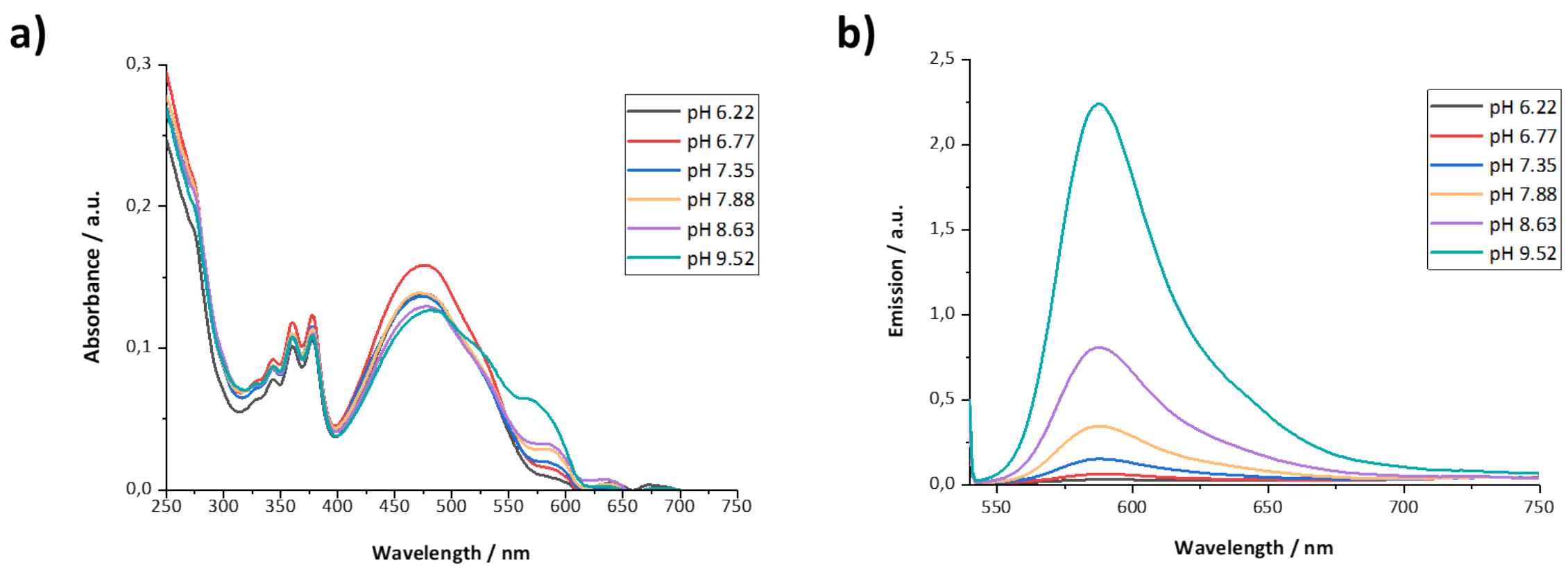

Figure S37. (a) UV–vis absorption spectra of *rac*-**5H-CF** at different pH values, showing a 470 nm maximum for the neutral species at lower pHs and a 580 nm maximum for the anionic species under basic conditions. (b) Fluorescence emission spectra (λ_{ex} = 530 nm) with a 580 nm maximum at basic pH, decreasing as the solution becomes less basic.

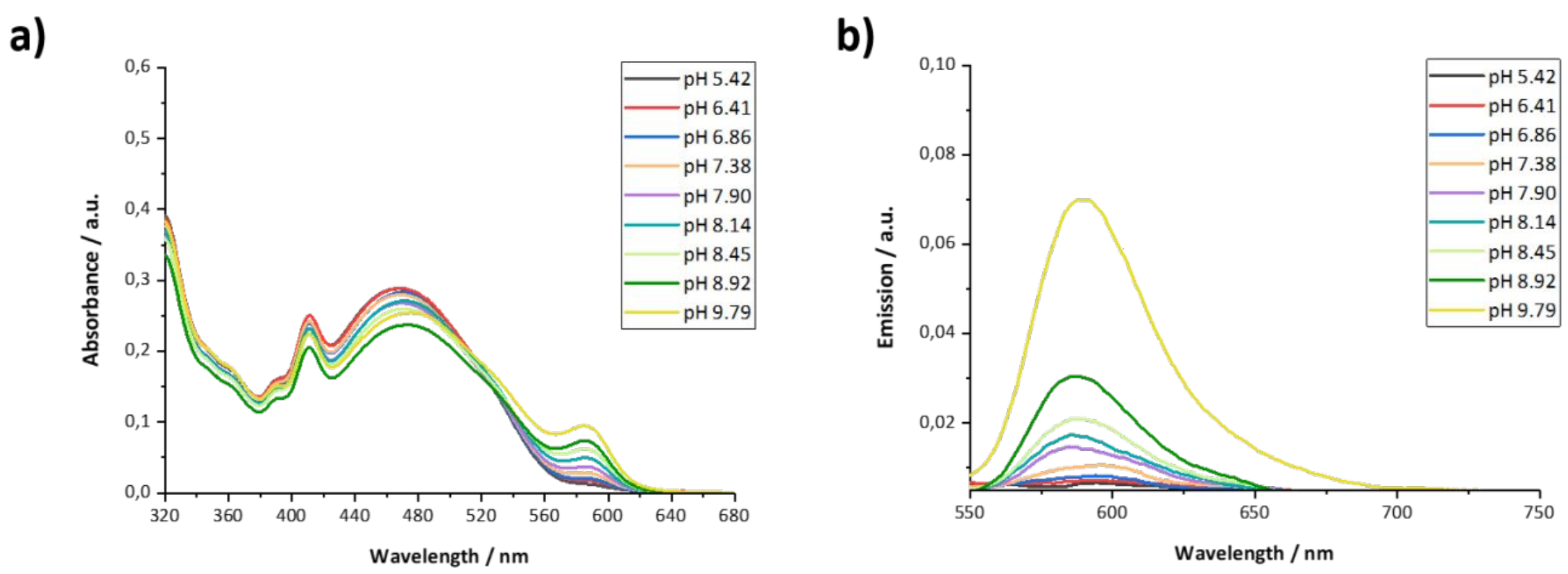

Figure S38. (a) UV–vis absorption spectra of *rac*-**7H-CF** at different pH values, showing a 470 nm maximum for the neutral species at lower pHs and a 580 nm maximum for the anionic species under basic conditions. (b) Fluorescence emission spectra (λ_{ex} = 530 nm) with a 580 nm maximum at basic pH, decreasing as the solution becomes less basic.

2.5.2. Determination of pK_a

The pK_a values were determined by identifying absorption and emission changes at 410, 470, and 580 nm as a function of pH. The fluorescence intensity at 580 nm showed a sigmoidal response characteristic of the acid–base equilibrium. The pK_a was then calculated using Equation 3.

$$y = \frac{f_1}{1 + 10^{\,x - pK_a}} + \frac{f_2}{1 + 10^{x - pK_b}} \tag{3}$$

where y denotes the measured absorbance or fluorescence signal, x represents the solution pH, and f_1 and f_2 correspond to the limiting values of the acidic and basic forms, respectively.

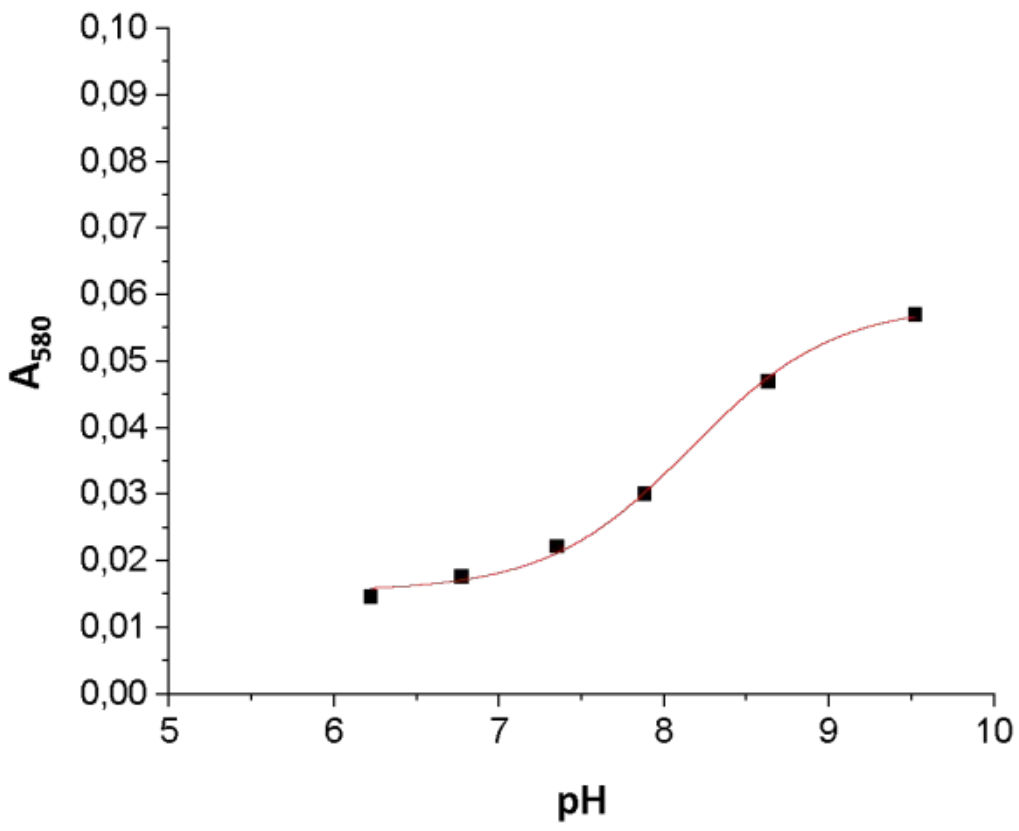

Model	NewFunction (User)
Equation	y=(f1/(1+10^(x-pK)))+(f2/(1+10^(pK-x)))
Plot	I580
f1	0.01543 ± 7.82986E-4
f2	0.05853 ± 0.0012
pK	8.16272 ± 0.05758
Reduced Chi-Sqr	1.18248E-6
R-Square (COD)	0.99756
Adj. R-Square	0.99594

Figure S39. Absorbance of *rac*-**5H-CF** at 580 nm as a function of pH, showing pK_a = 8.16.

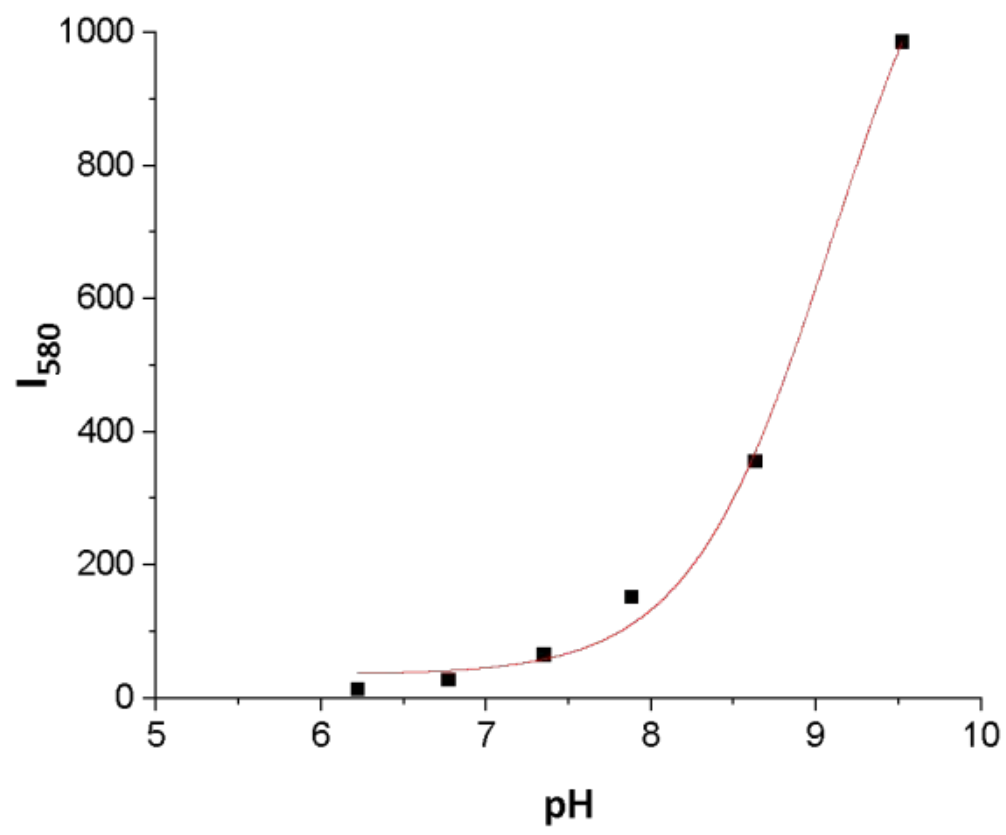

Model	NewFunction1 (User)
Equation	y=(f1/(1+10^(x-pK)))+(f2/(1+10^(pK-x)))
Plot	I580
f1	35.07449 ± 16.85649
f2	1341.02155 ± 102.14912
pK	9.09348 ± 0.10039
Reduced Chi-Sqr	899.96941
R-Square (COD)	0.99615
Adj. R-Square	0.99358

Figure S40. Fluorescence of *rac*-**5H-CF** at 580 nm as a function of pH, showing pK_a* = 9.10.

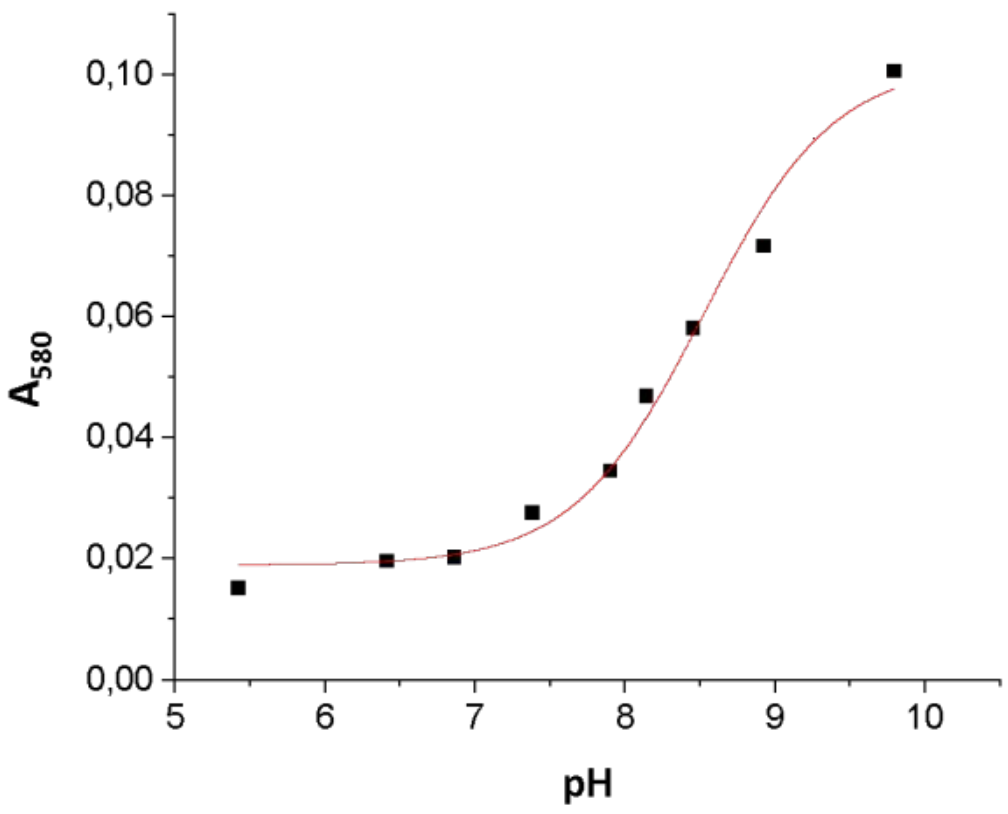

Model	NewFunction (User)
Equation	y=(f1/(1+10^(x-pK)))+(f2/(1+10^(pK-x)))
Plot	I580
f1	0.019 ± 0.00206
f2	0.10194 ± 0.00435
pK	8.52346 ± 0.08265
Reduced Chi-Sqr	1.4979E-5
R-Square (COD)	0.98622
Adj. R-Square	0.98163

Figure S41. Absorbance of *rac*-**7H-CF** at 580 nm as a function of pH, showing pK_a = 8.52.

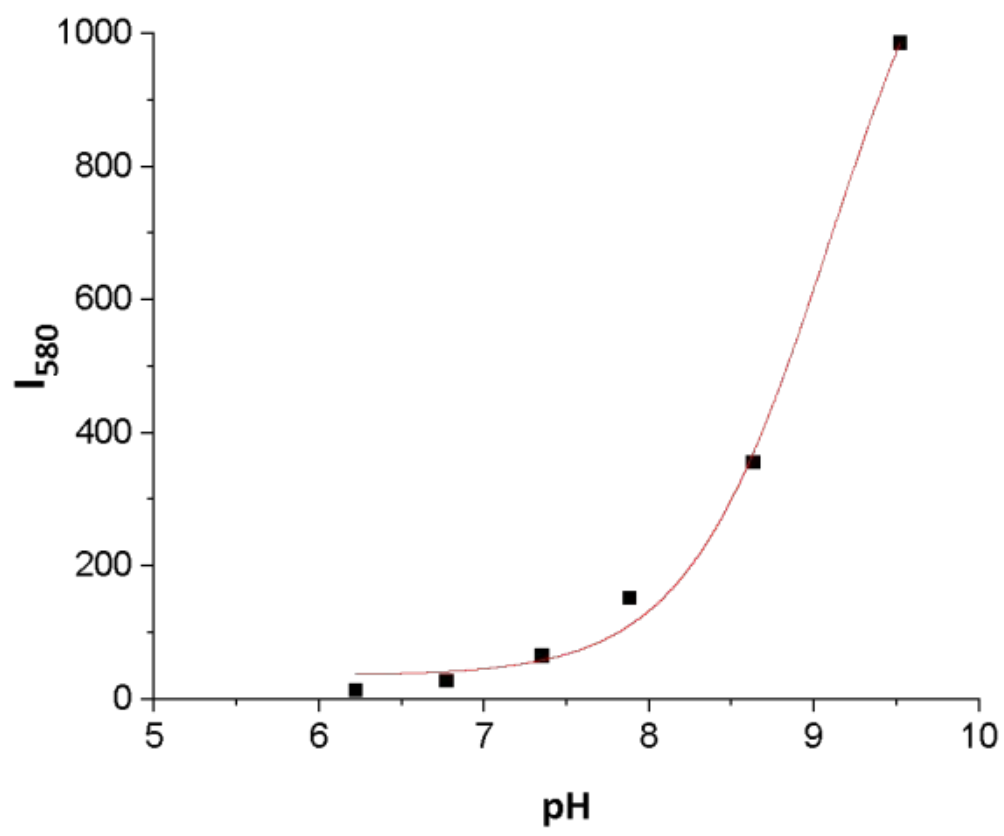

Model	NewFunction3 (User)
Equation	y=(f1/(1+10^(x-pK)))+(f2/(1+10^(pK-x)))
Plot	I580
f1	8.08289 ± 1.00742
f2	75.21763 ± 5.04726
pK	9.21682 ± 0.10032
Reduced Chi-Sqr	4.85678
R-Square (COD)	0.98823
Adj. R-Square	0.9843

Figure S42. Fluorescence of *rac*-**7H-CF** at 580 nm as a function of pH, showing pK_a* = 9.22.

3. CHIROPTICAL STUDIES

3.1. General Information

Chiroptical measurements of **7H-CF** enantiomers were carried out using an Olis DSM172 spectrophotometer. Electronic Circular Dichroism (ECD) spectra were recorded using a 150 W lamp in a 1.0 cm path length quartz cuvette at a concentration of 1.8 μM. Spectra were collected from 250 to 500 nm with a bandwidth of 10 nm, 250 data points, an integration time of 0.1 s, and 50 accumulated scans. Circularly Polarized Luminescence (CPL) spectra were recorded using a 490 nm LED as excitation source in a 1.0 cm path length quartz cuvette at a concentration of 1.8×10^{-5} M. Spectra were collected from 520 to 700 nm with a slit width of 3.0 mm, 380 data points, an integration time of 1.0 s, and 125 accumulated scans.

3.2. HPLC Separation of Enantiomers

Separation of the *rac*-**7H-CF** enantiomers was carried out by analytical chiral HPLC (Shimadzu) with UV detection at 480 nm. HPLC (Chiralpak IA, 250 × 4.6 mm, 5 μm; n-hexane/EtOAc = 3:7 (v/v); flow rate = 2.0 mL min^{-1}; λ = 480 nm) tR = 60.6 min (first enantiomer), 63.2 min (second enantiomer). Under these conditions, 1 mg of the racemic compound, dissolved in 1 mL of *n*-hexane/EtOAc (3:7, v/v) (1.54 mM solution), was separated by recirculating HPLC using multiple 200 μL injections.

The method relies on the recirculation capability of the HPLC system, in which consecutive cycles through the column resulted in complete separation of the enantiomers. Three 15 min recirculation cycles were performed, followed by a fourth cycle during which the separated enantiomers were collected.

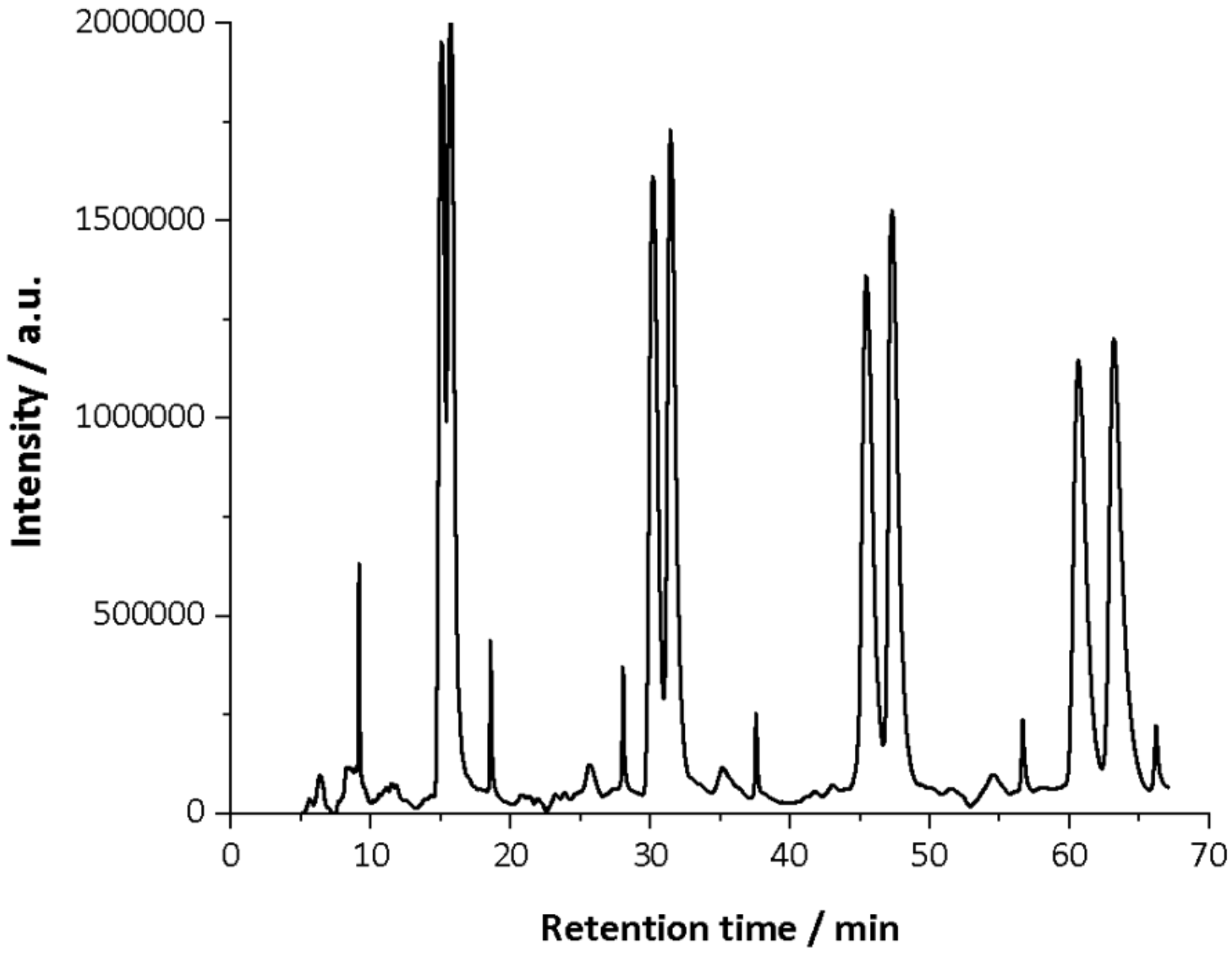

Figure S43. HPLC chromatogram showing the chiral separation of the *rac*-**7H-CF.**

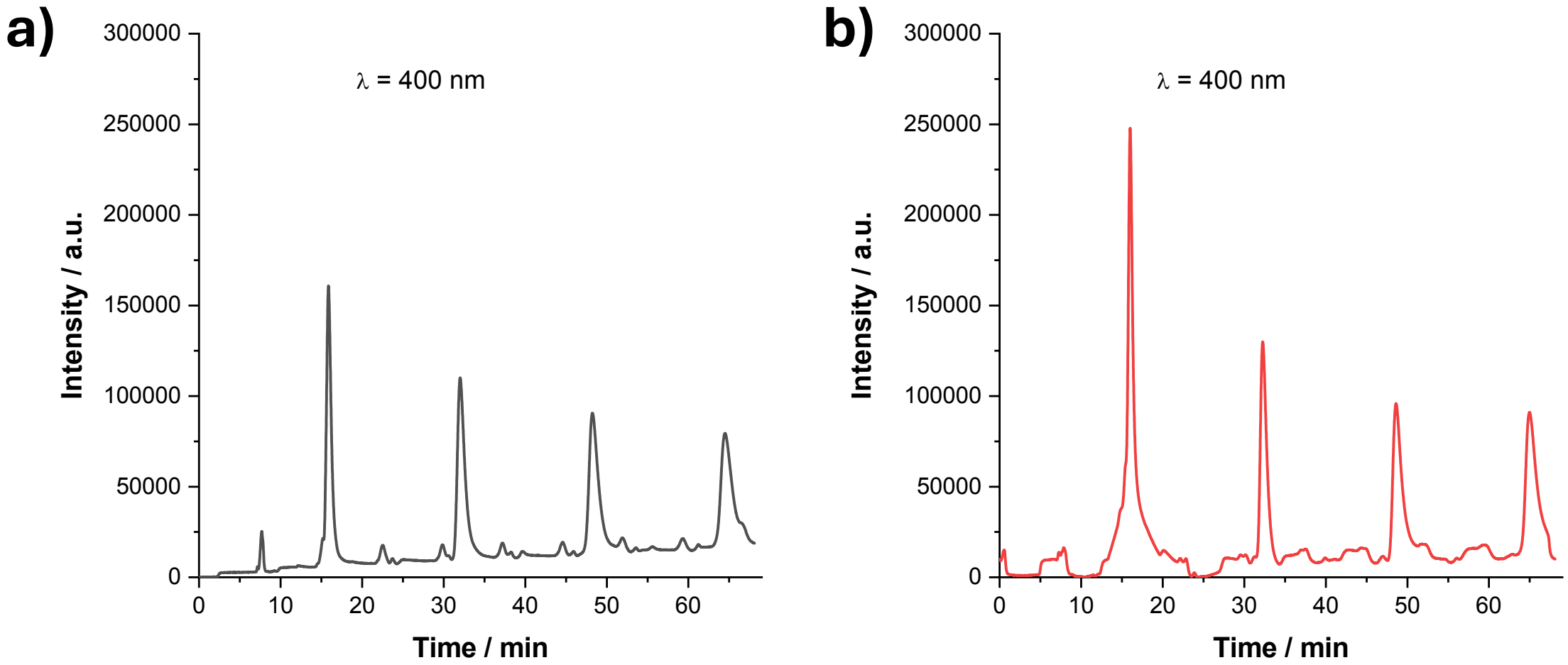

Figure S44. HPLC chromatogram of reinjected fraction of a) (*P*-) and b) *(M)*-**7H-CF.**

3.3. Electronic Circular Dichroism

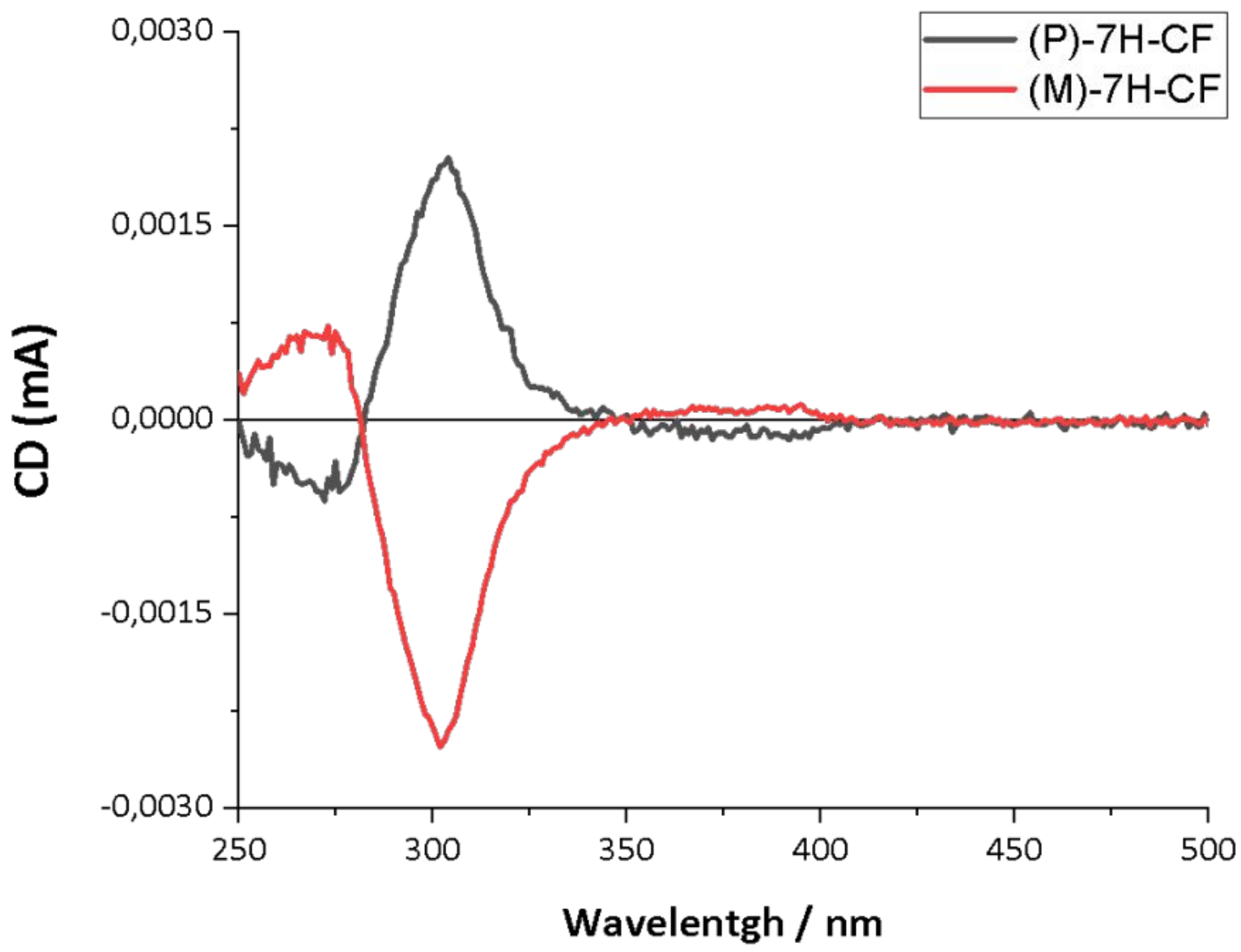

Figure S45. ECD of (*P*)-**7H-CF** (black) and (*M*)-**7H-CF** (red) in a basic solution of EtOH (NaOH was used as base).

3.4. Dissymmetry Factors

The absorption dissymmetry factor (g_{abs}) was then calculated using Equation 4.

$$g_{abs} = \frac{\Delta A}{A} \tag{4}$$

Where A is absorbance and ΔA refers to the differential absorption of left- and right-handed light.

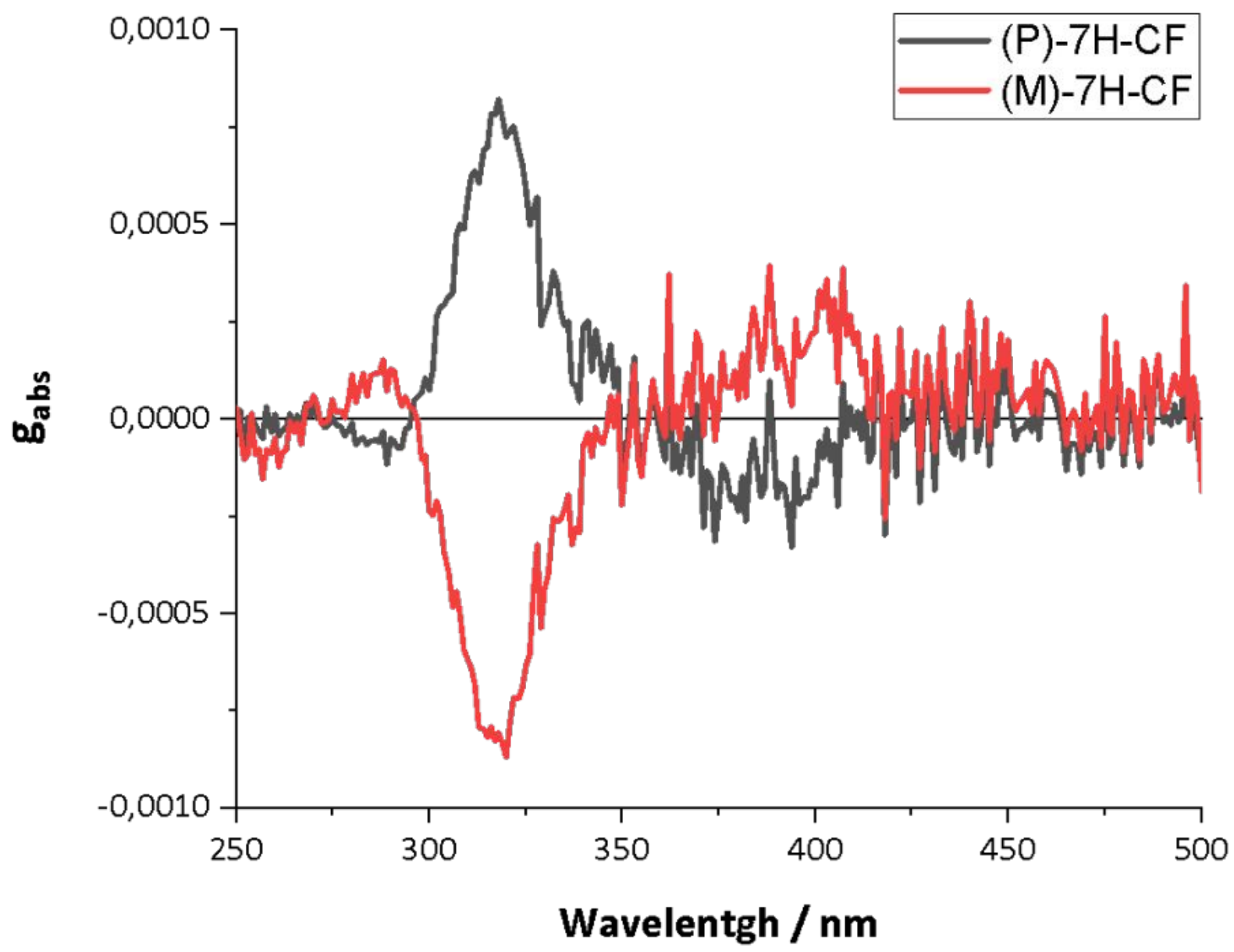

Figure S46. g_{abs} representation for both enantiomers of (*P*)-**7H-CF** (black) and (*M*)-**7H-CF** (red) at 1.8 μM in EtOH, showing mirror-image profiles with opposite signs, with maximum $|g_{abs}|$ values of approximately 8×10^{-4} at 318 nm.

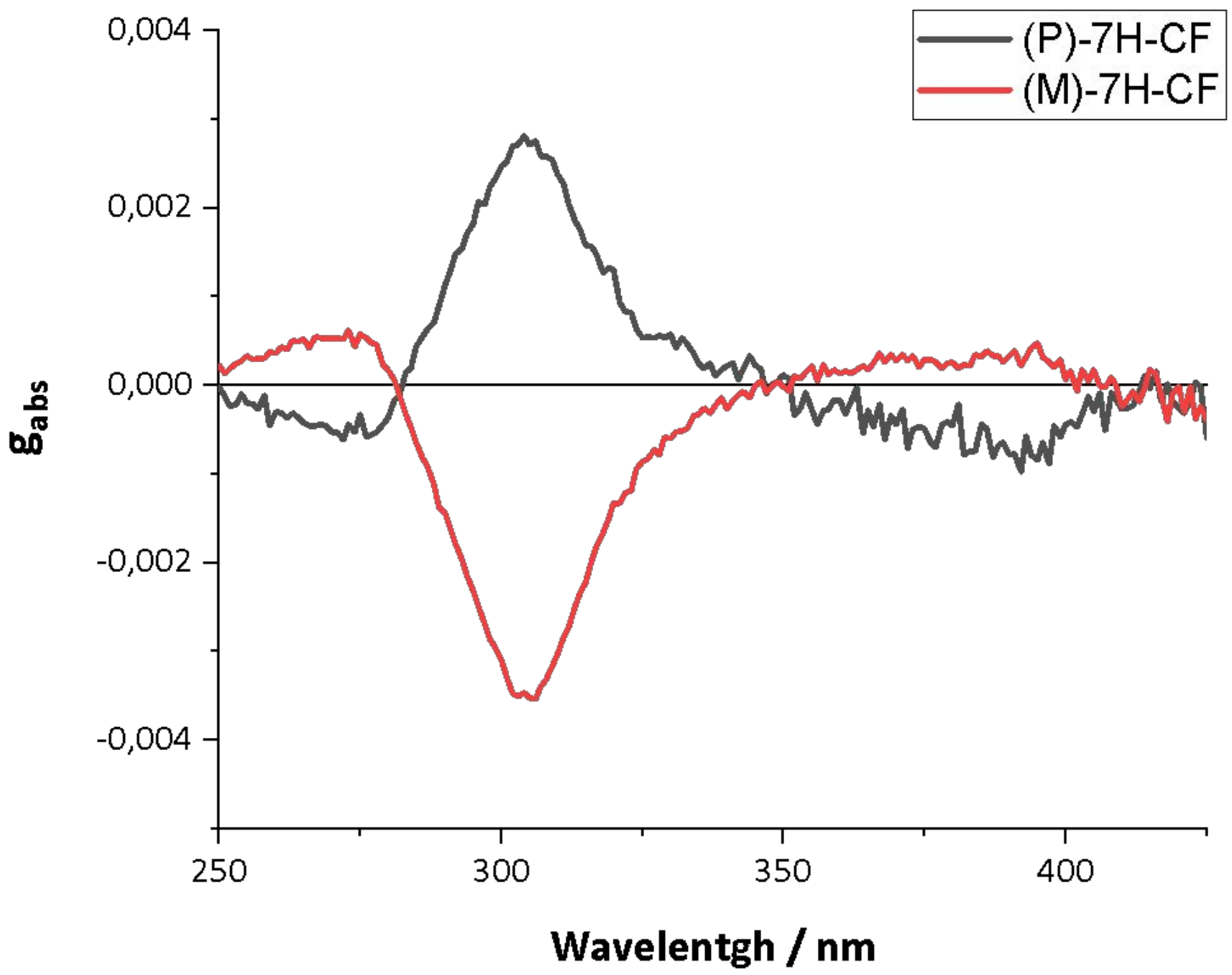

Figure S47. g_{abs} representation for both enantiomers of (*P*)-**7H-CF** (black) and (*M*)-**7H-CF** (red) in a basic solution of EtOH (NaOH was used as base), showing mirror-image profiles with maximum $|g_{abs}|$ values of approximately $3 \times$

10^{-3} at 318 nm.

The luminescence dissymmetry factor (g_{lum}) was then calculated using Equation 5.

$$g_{lum} = \frac{2\,(I_L - I_R)}{(I_L + I_R)} \quad (5)$$

Where I_L and I_R are the left- and right-circularly polarized emission intensities, respectively.

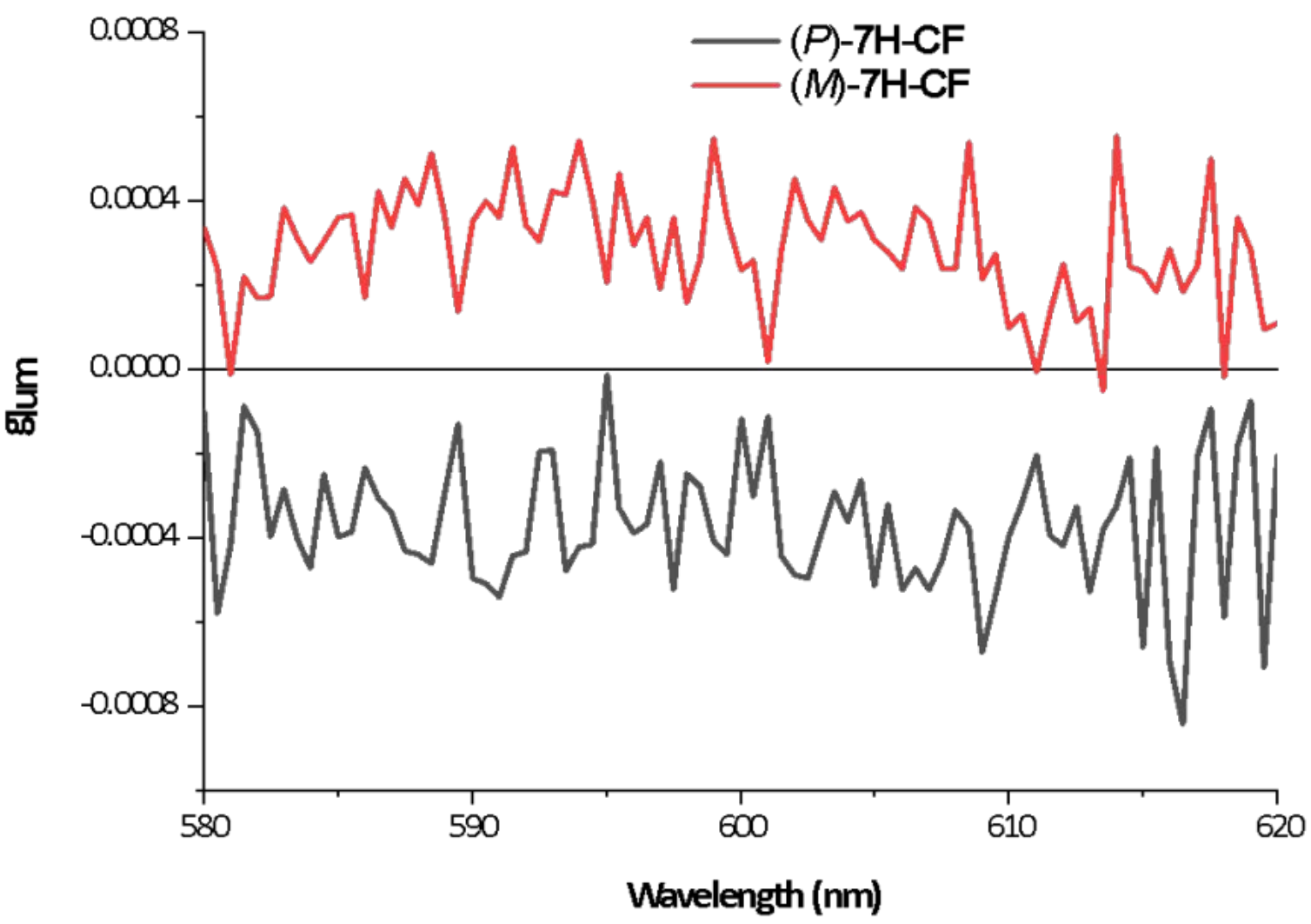

Figure S48. g_{lum} representation for (*P*)-**7H-CF** (black) and (*M*)-**7H-CF** (red) at 1.8 µM in EtOH, showing mirror-image profiles with opposite signs, with g_{lum} values on the order of 10^{-4}.

3.5. Thermal stability of (*P*)-7H-CF

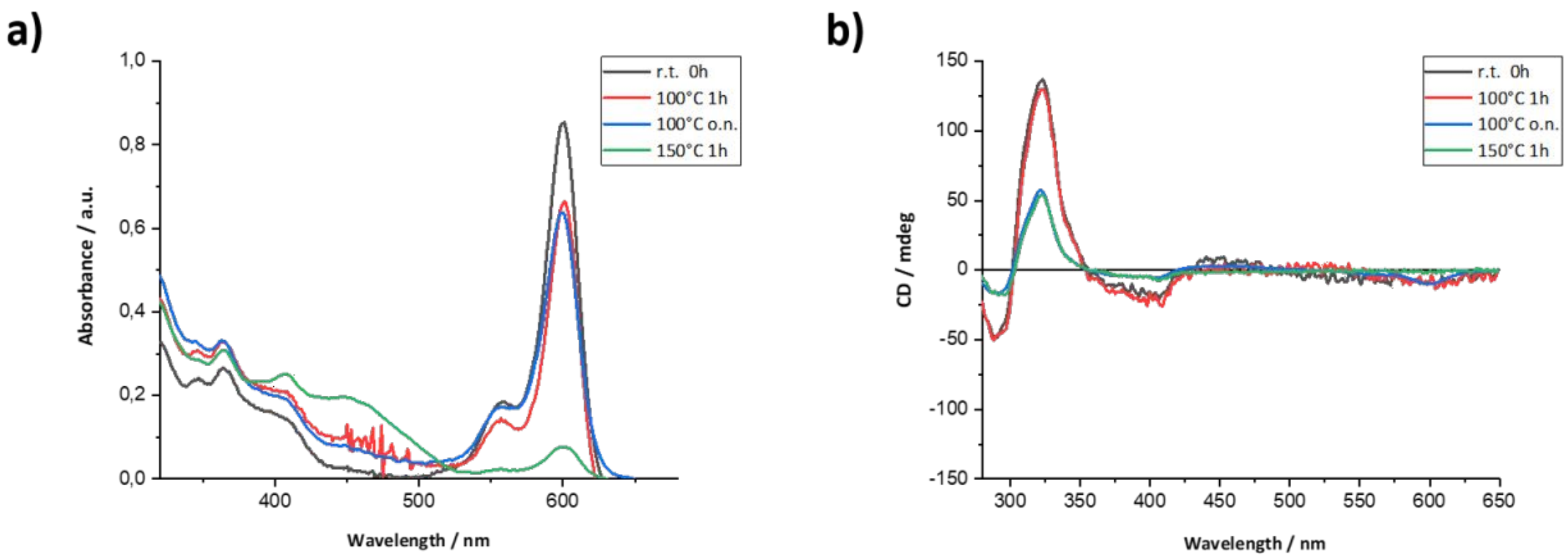

Figure S49. Absorbance profiles (a) and ECD spectra (b) of (*P*)-**7H-CF** in DMSO under different conditions.

4. LIFE-CELL IMAGING

A confocal microscope (Abberior scanning microscope; Abberior Instruments GmbH, Göttingen, Germany) equipped with a UPlanSApo oil-immersion objective (100×, NA 1.4) was used for live-cell imaging. The pinhole size was consistently set to 1 Airy unit (AU) for all measurements. Images were acquired using two-channel excitation and detection.

In the first channel (blue channel), a 375 nm pulsed laser (40 MHz) was used as the excitation source, and fluorescence was collected using an avalanche photodiode (APD) after passing through a 450/40 bandpass filter, corresponding to a detection range of 420–470 nm.

In the second channel (red channel), excitation was provided by a 530 nm pulsed laser (40 MHz), and fluorescence was detected using a hybrid photomultiplier tube (HPMT) after passing through a 655/25 bandpass filter, corresponding to a detection range of 580–630 nm.

Human cervix adenocarcinoma (HeLa, ATCC: CCL-2) cells were supplied by the Cell Culture Facility (University of Granada, Spain). Cells were grown at 37 °C in Dulbecco's modified Eagle's medium (DMEM) supplemented with 10% (v/v) fetal bovine serum (FBS), 2 mM glutamine, 100 U/mL penicillin, and 0.1 mg/mL streptomycin.

For live-cell imaging, HeLa cells were seeded in an 8-well plate (1,6 10^4 cells per well) and cultured in complete medium for 24 hours. Then, the culture medium was removed, and the cells were washed twice with HKBR buffer solution. A 1 x 10^{-3} mM stock solution of the compounds was prepared in DMSO and subsequently diluted to 1.0 x 10^{-6} M in HKBR buffer solution. For live-cell imaging, the culture medium was removed, and the cells were washed twice with HKBR buffer solution. Subsequently, 200 µL of the prepared solution was added to each well, and the cells were incubated for 3h at 37 °C. Cells were then imaged using the confocal microscope configuration described above.

Images were processed using *Fiji is just ImageJ* software [S4], employing custom macros for quantitative intensity analysis. First, a Gaussian smoothing filter (σ = 2 pixels) was applied to the red channel. Regions of interest (ROIs) were then defined based on intensity using automatic thresholding (Percentile, dark), generating binary masks with values of 0 for background and 1 for cells. Background subtraction was applied to the raw images, and both channels were subsequently multiplied by the ROI mask, resulting in images containing only cellular signal. Fluorescence intensities were then quantified within the defined ROIs.

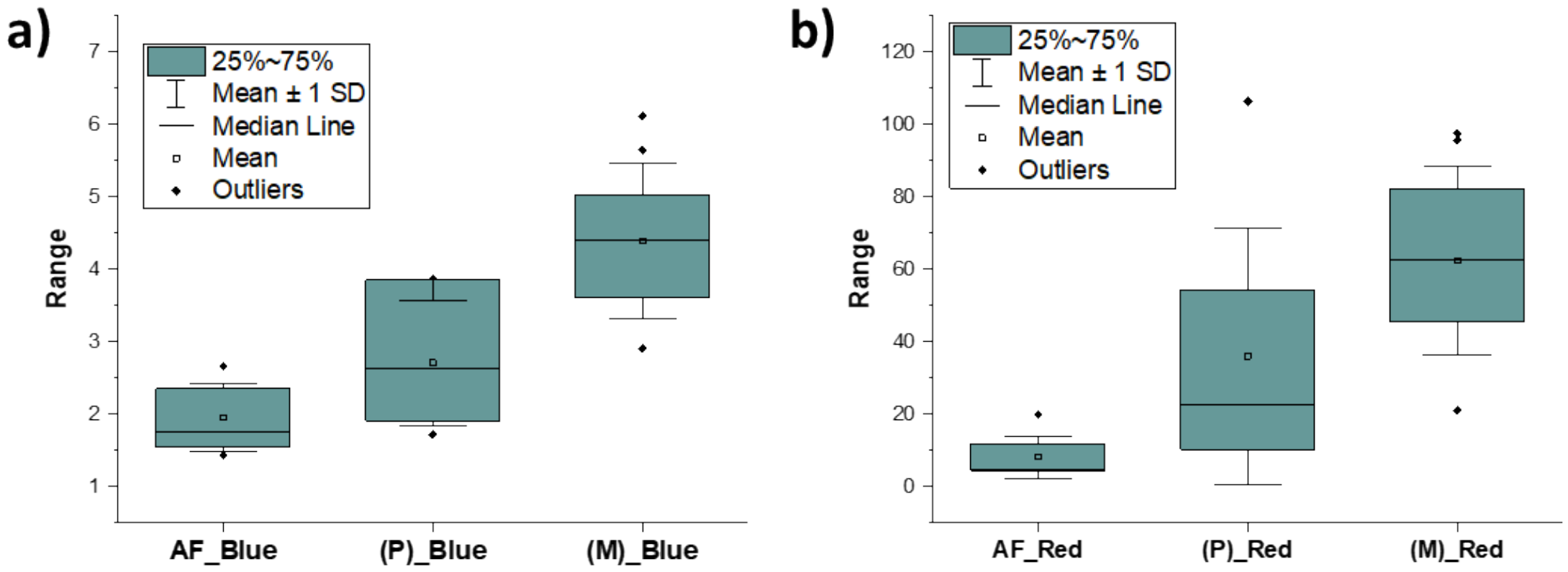

Figure S50. Quantification of both enantiomers in cells. AF = autofluorescence; (P)-Blue: emission of (*P*)-**7H-CF** in the blue channel; (P)-Red: emission of (*P*)-**7H-CF** in the red channel; (M)-Blue: emission of (*M*)-**7H-CF** in the blue channel; (M)-Red: emission of (*M*)-**7H-CF** in the red channel.

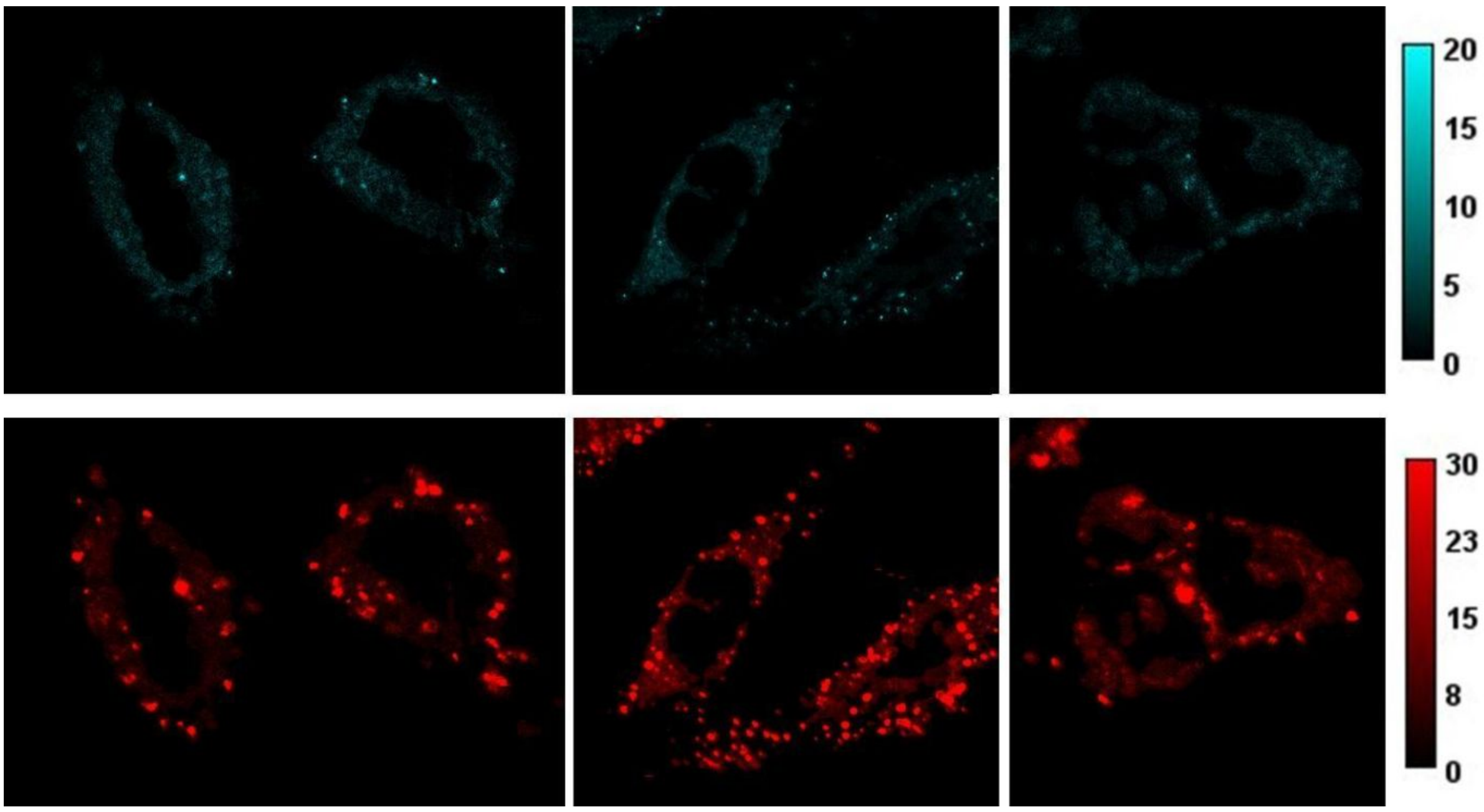

Figure S51. Additional examples of live-cell imaging of (*P*)-**7H-CF** in HeLa cells recorded in the blue (up) and red (down) regions.

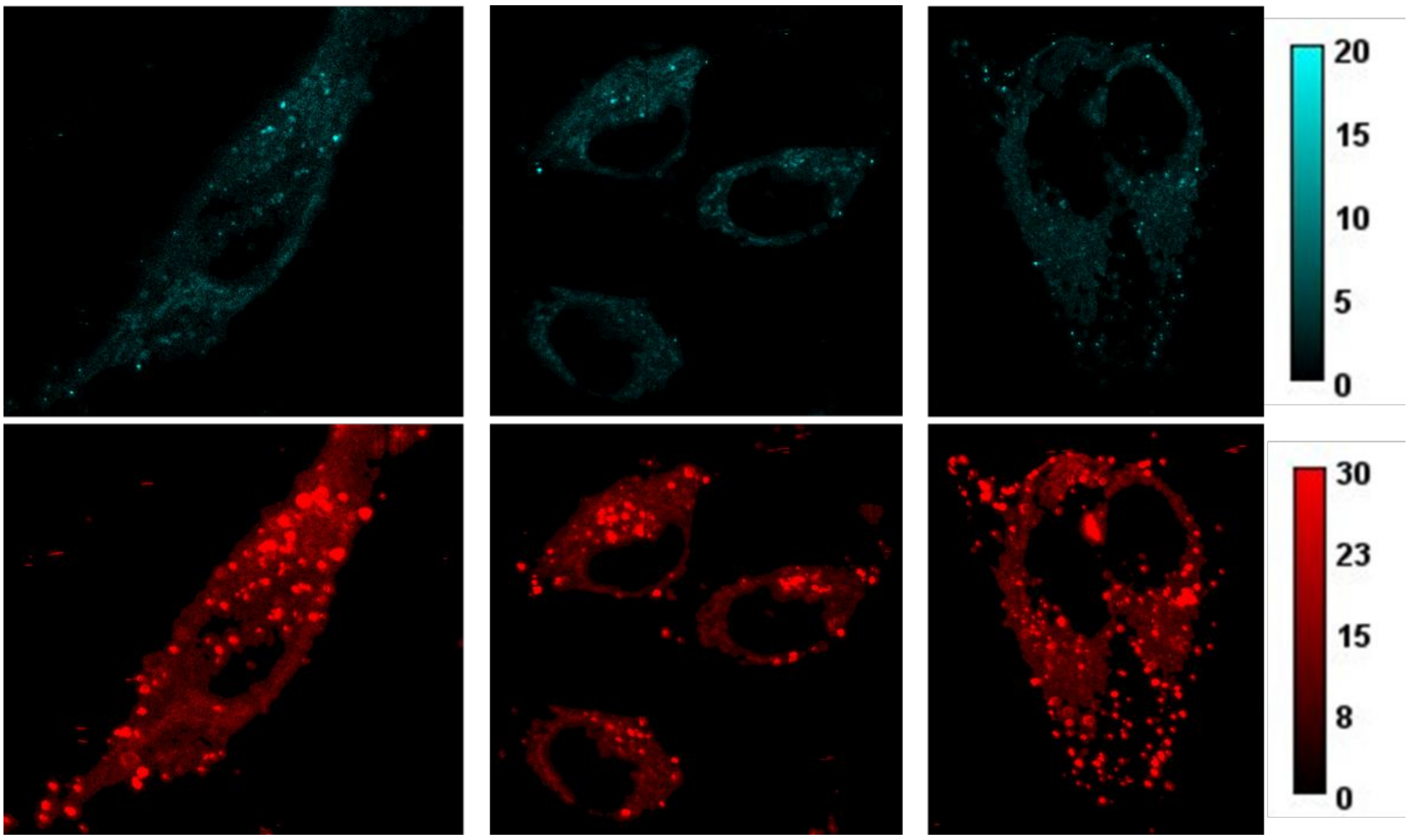

Figure S52. Additional examples of live-cell imaging of (*M*)-**7H-CF** in HeLa cells recorded in the blue (up) and red (down) regions.

5. THEORETICAL CALCULATIONS

All the calculations were performed using Gaussian16.[S5] The absolute configuration of the **7H-CF** helicenoid framework was assigned by comparison of the experimental electronic circular dichroism (ECD) spectrum with the TD-DFT calculated spectrum (50 first transitions) at the CAM-B3LYP/def2SVP level of theory, including PCM solvent corrections for ethanol (EtOH). In particular, the sign and shape of the lowest-energy Cotton effect were found to be consistent with the calculated spectrum of the (*M*) enantiomer, allowing the unambiguous assignment of the absolute helicity. The experimentally observed low-energy ECD band showed the same sign as that predicted for the (*M*) configuration, supporting the configurational assignment of the helicenoid structure in solution. Thus, first HPLC chromatographic peak, displaying a negative sign lower energy band, was determined to be enantiomer (*P*), whilst enantiomer (*M*) would correspond to second HPLC eluted fraction.

The first singlet excited state (S_1) of (*M*)-**7H-CF** was optimized by TD-DFT at the CAM-B3LYP/def2-SVP level of theory (gas phase). The calculation was performed using the optimized ground-state geometry and wavefunction stored in the checkpoint file (Geom=AllCheck, Guess=Read). The S_1 state was followed during the optimization using the TD (NStates=1, Root=1) formalism.

The racemization barriers of the helicenoid framework and the atropisomeric interconversion were investigated by TD-DFT calculations at the M06/TZVP level of theory, including solvent effects through the PCM model using dimethylsulfoxide (DMSO) as solvent. Geometry optimizations of all minima and transition states in all cases were performed without symmetry constraints, and the nature of each stationary point was confirmed by vibrational frequency analysis. Minima displayed no imaginary frequencies, whereas transition states exhibited a single imaginary frequency corresponding to the relevant stereochemical inversion coordinate. The activation barriers were obtained from the electronic energy differences between the optimized ground-state structures and the corresponding transition states. The calculated barrier for the helicenoid racemization process ($M \leftrightarrow P$ interconversion) was found to be 35.02 kcal mol^{-1} in PCM = DMSO. Intrinsic reaction coordinate (IRC) calculations were carried out to confirm the connectivity between transition states and the associated minima whenever convergence could be achieved.

Absolute configuration assignment for 7H-CF enantiomers

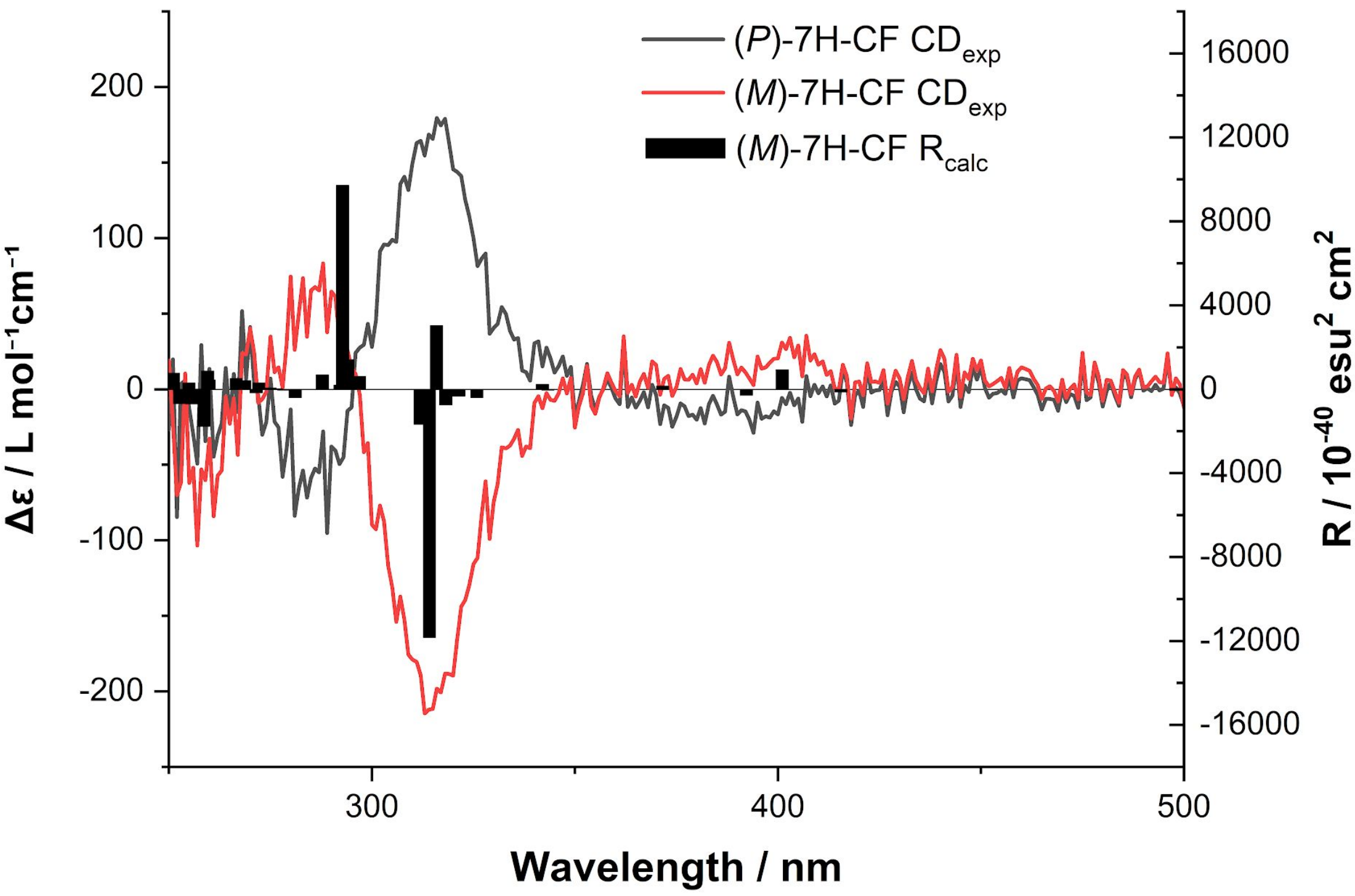

Figure S53. Experimental ECD of (*M*)-**7H-CF** (red line) and (*P*)-**7H-CF** (grey line), along with TD-DFT calculated (CAM-B3LYP/def2SVP, PCM=EtOH) rotational strength (black bars) after energy correction (0.25 eV redshift), employed for absolute configuration assignment.

Analysis of the lowest energy transition for compounds (CAM-B3LYP/def2SVP)

Neutral-(*M*)-**7H-CF** Ground State (PCM = EtOH):

State	λ (nm)	*f*	Orbitals contributing to the lowest energy transition	Gaussian Coefficient	Contribution (%)
S_1	385.48	0.6132	HOMO−1 → LUMO	0.69332	48.1
S_2	371.07	0.0826	HOMO → LUMO	0.63924	40.9

Transition electric dipole moment (esu cm)	Transition magnetic dipole moment (erg G⁻¹)	Angle between moments (°)	Rotatory Strength (erg esu cm G⁻¹)	Calculated g_{abs} value
$7.09038\cdot10^{-18}$	$2.7572\cdot10^{-20}$	90.32513439	$-1.10926\cdot10^{-20}$	$-1.0\cdot10^{-4}$

Anionic-(*M*)-**7H-CF** Ground State (PCM = EtOH):

State	λ (nm)	*f*	Orbitals contributing to the lowest energy transition	Gaussian Coefficient	Contribution (%)
S_1	467.05	0.7274	HOMO → LUMO	0.69343	48.1
S_2	383.78	0.0321	HOMO → LUMO+1	0.67304	45.3

Transition electric dipole moment (esu cm)	Transition magnetic dipole moment (erg G⁻¹)	Angle between moments (°)	Rotatory Strength (erg esu cm G⁻¹)	Calculated g_{abs} value
$8.50039 \cdot 10^{-18}$	$2.58386 \cdot 10^{-20}$	87.96739265	$7.79031 \cdot 10^{-39}$	$4.0 \cdot 10^{-4}$

Neutral-(*M*)-**7H-CF** Excited State (gas phase):

State	λ (nm)	*f*	Orbitals contributing to the lowest energy transition	Gaussian Coefficient	Contribution (%)
S_1	395.71	0.0013	HOMO−5 → LUMO	−0.64705	41.9

Transition electric dipole moment (esu cm)	Transition magnetic dipole moment (erg G⁻¹)	Angle between moments (°)	Rotatory Strength (erg esu cm G⁻¹)	Calculated g_{lum} value
$3.28829 \cdot 10^{-19}$	$5.77085 \cdot 10^{-21}$	28.50624223	$1.66757 \cdot 10^{-39}$	$6.2 \cdot 10^{-2}$

Anionic-(*M*)-**7H-CF** Excited State (gas phase):

State	λ (nm)	*f*	Orbitals contributing to the lowest energy transition	Gaussian Coefficient	Contribution (%)
S_1	485.73	0.0869	HOMO → LUMO	0.67933	46.1

Transition electric dipole moment (esu cm)	Transition magnetic dipole moment (erg G⁻¹)	Angle between moments (°)	Rotatory Strength (erg esu cm G⁻¹)	Calculated g_{lum} value
$2.99535 \cdot 10^{-18}$	$9.33266 \cdot 10^{-21}$	71.78163106	$8.73972 \cdot 10^{-39}$	$3.9 \cdot 10^{-3}$

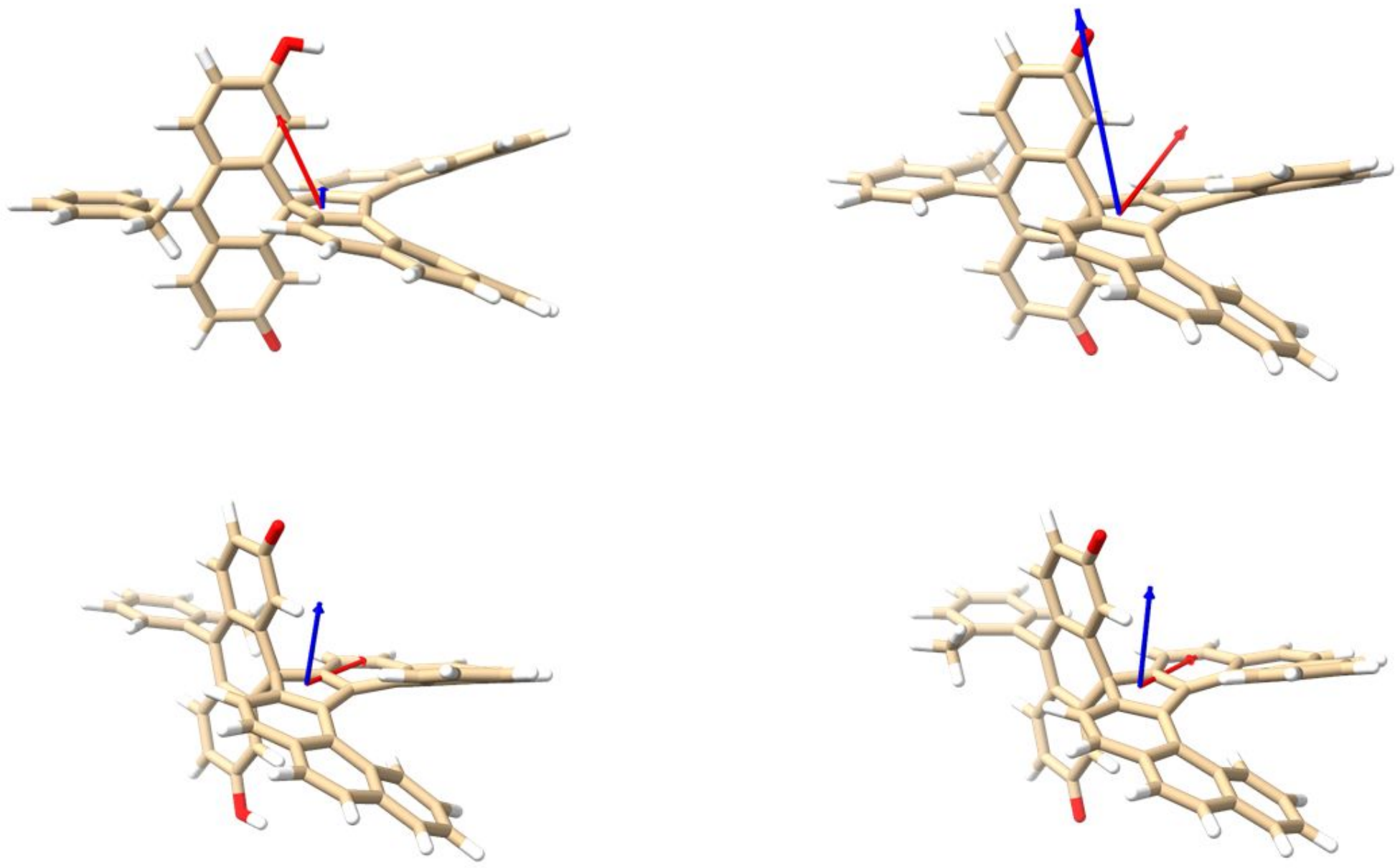

Figure S54. Calculated electric (blue headed arrow) and magnetic (red headed arrow) transition dipole moments (−***m***) for the lowest energy transition of neutral (left) and anionic (right) (*M*)-**7H-CF** in the ground (bottom) and excited (upper part) states. Magnitude of both ⌠ and **m** were multiplied by 5 in order to make the vectors visible.

Frontier molecular orbitals of (M)-7H-CF at the level of theory CAM-B3LYP/def2SVP

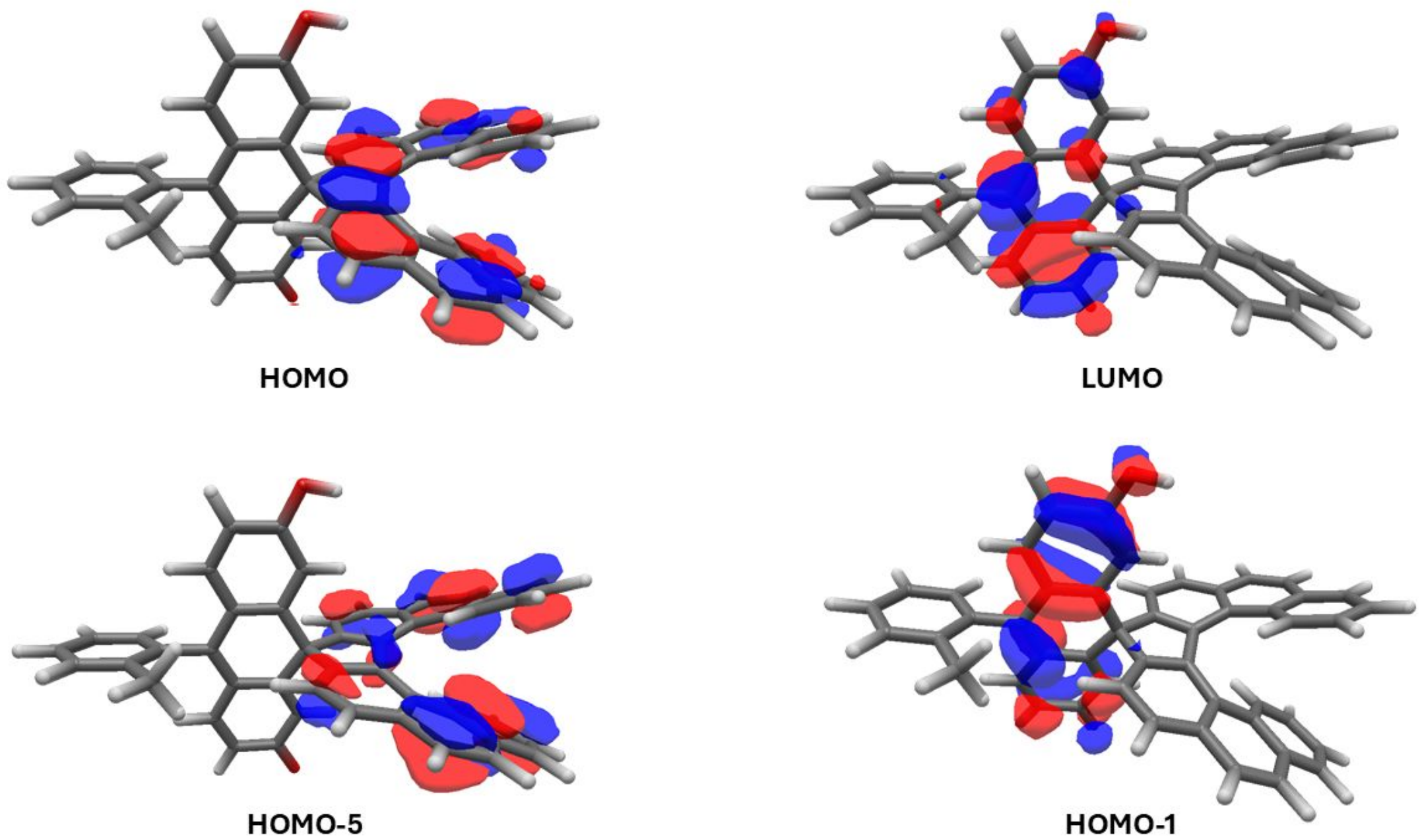

Figure S55. Frontier Molecular Orbitals for neutral (*M*)-**7H-CF**.

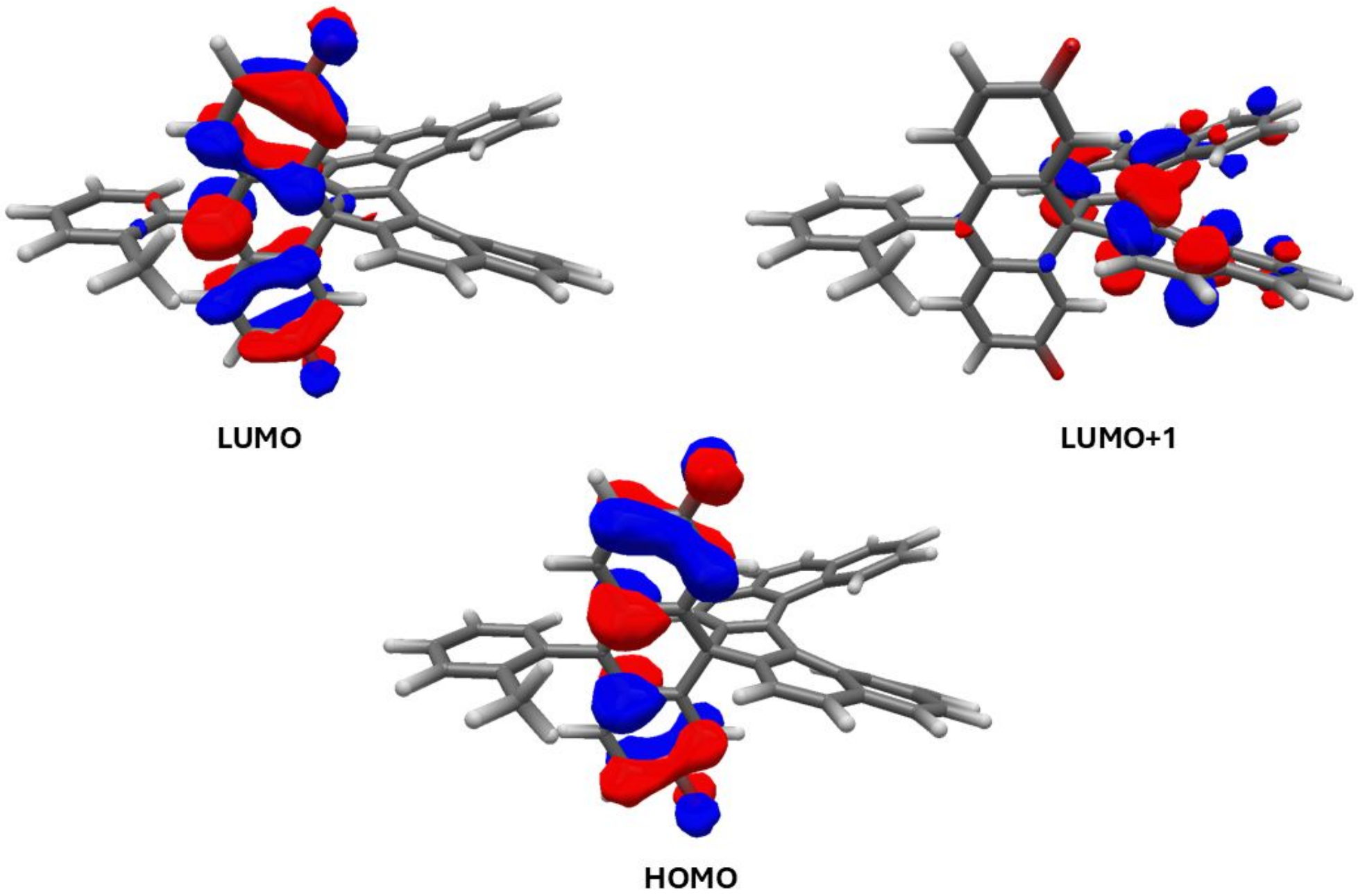

Figure S56. Frontier Molecular Orbitals for anionic (*M*)-**7H-CF**.

TD-DFT calculations revealed that the low-lying excited states of both the neutral and anionic ground-state structures are mainly described by single orbital excitations. For the neutral species, the S_1 state (385.48 nm, f = 0.6132) is predominantly characterized by a HOMO−1 → LUMO transition (CI coefficient = 0.693, contribution ≈ 48.1%), whereas the S_2 state (371.07 nm, f = 0.0826) is mainly associated with a HOMO → LUMO excitation (CI coefficient = 0.639, contribution ≈ 40.9%). In the anionic ground-state structure, the S_1 state (467.05 nm, f = 0.7274) is dominated by a HOMO → LUMO transition (CI coefficient = 0.693, contribution ≈ 48.1%), while the S_2 state (383.78 nm, f = 0.0321) is primarily described by a HOMO → LUMO+1 excitation (CI coefficient = 0.673, contribution ≈ 45.3%). Excited-state calculations performed on the optimized S_1 geometries indicate that the lowest excited state of the neutral species is mainly governed by a HOMO−5 → LUMO excitation (CI coefficient = −0.647, contribution ≈ 41.9%), whereas the corresponding excited state of the anion retains a dominant HOMO → LUMO character (CI coefficient = 0.679, contribution ≈ 46.1%). Overall, the results indicate that electronic excitations are largely governed by a single dominant orbital transition, with only minor contributions from higher-order configurations.

Optimized atomic coordinates

Neutral (*M*)-**7H-CH** (Ground state, CAM-B3LYP/def2SVP, PCM = EtOH)

C	-1.57216200	-2.33444600	-1.96993700
C	-2.09499900	-1.54865100	-0.90287300
C	-0.20437800	-2.27276000	-2.31399300
C	0.66507000	-1.48563700	-1.59293500
C	0.13542800	-0.63646000	-0.62005800
C	-1.23449900	-0.54293100	-0.36100800
C	0.93900900	0.26634100	0.31362000
C	-0.17941400	0.98174000	1.04747400
C	-1.43380200	0.63778300	0.53816500
C	-0.01176300	1.90516000	2.08101800
C	-1.13237700	2.50323600	2.61050300
C	-2.39895200	2.30907300	2.01667100
C	-2.55543800	1.45479100	0.88788300
C	-3.53911800	3.00234100	2.55499300
C	-4.75136300	2.93290400	1.95878100
C	-4.90559800	2.25080600	0.70442400
C	-3.80588900	1.55026500	0.14077900
C	1.77054400	-0.62340500	1.23813300
C	3.21256700	-0.72296000	1.05252300
C	3.87509700	0.09672900	0.17425700
C	3.17099400	1.12083300	-0.59071400
C	1.77152200	1.25087100	-0.50360100
C	3.87669200	2.01869500	-1.41777900
C	3.23390300	3.00816000	-2.13316100
C	1.84207800	3.12985300	-2.03861400
C	1.12577600	2.25014300	-1.22568300
C	1.15785700	-1.37260800	2.18439000
C	1.86828000	-2.32797300	3.03779500
C	3.30856100	-2.44992000	2.79458000
C	3.92515800	-1.69527200	1.86395800
O	1.24821900	4.10538300	-2.74518100
O	1.28779700	-2.98895600	3.89493400
C	5.35751800	-0.00643500	0.00928500
C	5.91322600	-0.78937300	-1.01967700
C	7.30562100	-0.84060800	-1.13324500
C	8.13407600	-0.13901500	-0.26145100
C	7.57622800	0.63372900	0.75235500
C	6.19186800	0.69640500	0.88296500
C	5.03771400	-1.56146500	-1.97060300
C	-3.42267500	-1.89238100	-0.40266000
C	-4.24552300	-2.77524500	-1.15169000
C	-3.73370300	-3.39031300	-2.34442100
C	-2.44045500	-3.21833000	-2.70169700
C	-6.12281400	2.33758600	-0.00688100
C	-6.24501700	1.80801400	-1.27277800
C	-5.13392300	1.19169300	-1.87242300
C	-3.94760400	1.06709300	-1.17950500

C	-3.89669500	-1.47136400	0.85998600
C	-5.53565800	-3.10349100	-0.67980600
C	-5.98971900	-2.63201100	0.53250600
C	-5.14660100	-1.83054600	1.32031600
H	0.16070000	-2.90014400	-3.12974200
H	1.73770700	-1.50176500	-1.79398900
H	0.98264300	2.12028200	2.47657400
H	-1.05185800	3.17728500	3.46579500
H	-3.39839100	3.58819000	3.46596900
H	-5.61521400	3.44865500	2.38389000
H	4.96086700	1.93300700	-1.49063000
H	3.78654600	3.70102200	-2.76889300
H	0.04025100	2.35204200	-1.16252100
H	0.07974100	-1.30876200	2.34444400
H	3.85528500	-3.17266100	3.40359800
H	4.99983800	-1.79609700	1.70844600
H	0.29152400	4.09586300	-2.60292800
H	7.74769800	-1.44841400	-1.92640400
H	9.21853500	-0.19825100	-0.37563400
H	8.21518100	1.18763400	1.44300800
H	5.74573900	1.30068600	1.67610800
H	4.41705700	-2.29680200	-1.43604100
H	4.35046600	-0.89526000	-2.51446000
H	5.64081400	-2.10233000	-2.71152800
H	-4.38981900	-4.05057300	-2.91577400
H	-2.02423100	-3.74857300	-3.56109100
H	-6.96224000	2.86060200	0.45726600
H	-7.18771800	1.89140200	-1.81751200
H	-5.20501200	0.81223200	-2.89383900
H	-3.09621900	0.60869700	-1.67655800
H	-3.25437600	-0.87250200	1.50102700
H	-6.15978100	-3.76559700	-1.28460100
H	-6.98462900	-2.90240200	0.89230200
H	-5.47712300	-1.49274700	2.30466500

Zero-point correction= 0.646032 (Hartree/Particle)
Thermal correction to Energy= 0.682867
Thermal correction to Enthalpy= 0.683811
Thermal correction to Gibbs Free Energy= 0.576446
Sum of electronic and zero-point Energies= -2032.546774
Sum of electronic and thermal Energies= -2032.509939
Sum of electronic and thermal Enthalpies= -2032.508995
Sum of electronic and thermal Free Energies= -2032.616360

Anionic (*M*)-**7H-CH** (Ground state, CAM-B3LYP/def2SVP, PCM = EtOH)

C	-1.60536400	2.40868300	1.91155700
C	-2.11721100	1.58063300	0.87135600
C	-0.23936900	2.36635500	2.26525000

C	0.63764800	1.55762200	1.57808400
C	0.12001500	0.66899900	0.63392100
C	-1.24860400	0.56013500	0.37133500
C	0.93395000	-0.26664600	-0.25580500
C	-0.17505500	-1.00661600	-0.97318900
C	-1.43573500	-0.65147700	-0.48828100
C	0.00196300	-1.96437500	-1.97397100
C	-1.11075600	-2.58560700	-2.49241200
C	-2.38330100	-2.37746500	-1.91560400
C	-2.55156000	-1.48478500	-0.81904900
C	-3.51617400	-3.09467300	-2.43730700
C	-4.73368200	-3.01104900	-1.85330300
C	-4.90015800	-2.28629700	-0.62480900
C	-3.80737300	-1.56118000	-0.07858300
C	1.75951900	-1.21536900	0.62091600
C	3.16030500	-0.95783400	0.81514300
C	3.82515200	0.03514800	0.06935100
C	3.17461000	0.78887600	-0.92678800
C	1.77535000	0.60454900	-1.19388300
C	3.88232600	1.71735300	-1.75673500
C	3.28545000	2.38888100	-2.77939600
C	1.87810600	2.20279600	-3.09373300
C	1.17576200	1.27232400	-2.22435500
C	1.14872700	-2.23915900	1.28982100
C	1.83892200	-3.10933800	2.22878000
C	3.24732900	-2.80601000	2.42255900
C	3.85544200	-1.79011900	1.75124800
O	1.31290200	2.79407200	-4.02827800
O	1.26207100	-4.03629900	2.82078700
C	5.28061800	0.27867700	0.32452800
C	6.27284500	-0.43336100	-0.37509700
C	7.61353700	-0.15808700	-0.08964900
C	7.97674500	0.79359200	0.85996100
C	6.99038400	1.49388400	1.54737300
C	5.64983600	1.23295100	1.27605000
C	5.90718000	-1.46704800	-1.40676900
C	-3.44315800	1.90146700	0.35043700
C	-4.27510100	2.80803600	1.06049900
C	-3.77427100	3.46867100	2.23339100
C	-2.48250900	3.31477200	2.60445100
C	-6.12205400	-2.35373100	0.08088200
C	-6.25578300	-1.78070400	1.32652700
C	-5.15152300	-1.13858400	1.91179600
C	-3.96085700	-1.03319500	1.22318900
C	-3.90700100	1.43290600	-0.89924700
C	-5.56322900	3.11308900	0.56763900
C	-6.00709800	2.59573500	-0.62970200
C	-5.15501700	1.76948600	-1.38146600
H	0.11755900	3.02485500	3.06005000
H	1.70954400	1.58410200	1.78237600
H	1.00091300	-2.18825000	-2.35268900

H	-1.02047300	-3.28817300	-3.32363900
H	-3.36566300	-3.71159700	-3.32611000
H	-5.59177200	-3.54603100	-2.26629400
H	4.94420600	1.87764900	-1.56063500
H	3.84787600	3.08838900	-3.40270400
H	0.11170700	1.13873300	-2.43191700
H	0.08484200	-2.44221800	1.15189700
H	3.80069900	-3.43072700	3.12799500
H	4.91731200	-1.60017100	1.91871600
H	8.39032600	-0.70648700	-0.62839600
H	9.03269000	0.98678700	1.06136100
H	7.26070300	2.24309300	2.29442500
H	4.86963100	1.77908400	1.81138900
H	5.30787800	-1.02682200	-2.21804200
H	6.80520000	-1.91698800	-1.85073600
H	5.30182400	-2.27386500	-0.96663900
H	-4.43678200	4.14668100	2.77597300
H	-2.07402700	3.87774100	3.44676200
H	-6.95574300	-2.89701800	-0.37037000
H	-7.20193200	-1.84965100	1.86743300
H	-5.23117600	-0.72433100	2.91902100
H	-3.11432400	-0.55441300	1.70916000
H	-3.25748500	0.81299500	-1.51236200
H	-6.19432100	3.79426100	1.14349100
H	-7.00060000	2.84855100	-1.00598100
H	-5.47702700	1.39417300	-2.35506100

Zero-point correction= 0.632475 (Hartree/Particle)
Thermal correction to Energy= 0.669069
Thermal correction to Enthalpy= 0.670013
Thermal correction to Gibbs Free Energy= 0.563134
Sum of electronic and zero-point Energies= -2032.084350
Sum of electronic and thermal Energies= -2032.047756
Sum of electronic and thermal Enthalpies= -2032.046812
Sum of electronic and thermal Free Energies= -2032.153692

Neutral (*M*)-**7H-CH** (Ground state, M06/TZVP, PCM = DMSO)

C	-1.62860200	-2.38620200	-1.91900100
C	-2.12078000	-1.56284500	-0.87051500
C	-0.27006900	-2.36591400	-2.27538700
C	0.61957900	-1.57672500	-1.59162400
C	0.11696000	-0.68829300	-0.65231500
C	-1.24283500	-0.55971100	-0.37707500
C	0.94100000	0.23720100	0.22949700
C	-0.14782000	1.00063300	0.93906900
C	-1.41133300	0.64578300	0.47514000
C	0.05802800	1.96671700	1.91315300
C	-1.03853900	2.59281000	2.44662000

C	-2.31813500	2.37438500	1.90798800
C	-2.51178700	1.47875600	0.82185600
C	-3.43769800	3.06799900	2.46220300
C	-4.67150700	2.94694600	1.93192600
C	-4.86951500	2.21684200	0.72309400
C	-3.78038600	1.53511900	0.12700500
C	1.75775500	-0.63019100	1.17258100
C	3.16793400	-0.81630000	0.93074300
C	3.82898600	-0.06836700	-0.01163400
C	3.15702100	0.95084900	-0.78057500
C	1.77486800	1.15608100	-0.64108200
C	3.86772700	1.77280800	-1.67226900
C	3.24687800	2.76159100	-2.39211900
C	1.87468200	2.95426800	-2.24295300
C	1.14978200	2.14885300	-1.37363300
C	1.14867500	-1.29760100	2.17763600
C	1.84651900	-2.23161000	3.04282900
C	3.25850200	-2.42825500	2.75641000
C	3.86761900	-1.76069500	1.76120400
O	1.30360800	3.93347400	-2.96501900
O	1.27258800	-2.82510800	3.96022700
C	5.27407000	-0.29559500	-0.26669800
C	6.25539500	0.40512500	0.44180000
C	7.58892900	0.14875300	0.14099500
C	7.94755000	-0.77064700	-0.83135900
C	6.96769600	-1.45838400	-1.52807200
C	5.63408400	-1.21651500	-1.24384100
C	5.88527800	1.39345900	1.50275600
C	-3.44071000	-1.85472800	-0.35381500
C	-4.30388600	-2.70197500	-1.09043300
C	-3.82165600	-3.35662500	-2.26230600
C	-2.52617900	-3.24862400	-2.62102400
C	-6.11917800	2.22833000	0.08077200
C	-6.28717200	1.64418000	-1.14876100
C	-5.18943900	1.05977600	-1.78859200
C	-3.96781400	1.01163700	-1.16365900
C	-3.88422000	-1.40147600	0.89986800
C	-5.60518700	-2.94645800	-0.62021100
C	-6.03310700	-2.42897100	0.57595500
C	-5.14978000	-1.67610700	1.35629400
H	0.06720100	-3.02015000	-3.07213000
H	1.68430600	-1.61698300	-1.80075500
H	1.06278200	2.18691900	2.25993400
H	-0.93369500	3.29838800	3.26378900
H	-3.26628400	3.69204500	3.33314700
H	-5.52177300	3.45501200	2.37487400
H	4.93467700	1.62332400	-1.79030800
H	3.79998700	3.39623900	-3.07348300
H	0.07891100	2.30499900	-1.27400700
H	0.08746700	-1.16765700	2.37161800
H	3.79859100	-3.13000200	3.38233800

H	4.92445400	-1.91591000	1.57191700
H	0.35736200	3.97850900	-2.78511700
H	8.35957000	0.68565900	0.68503100
H	8.99514700	-0.94947400	-1.04453500
H	7.23815800	-2.17973700	-2.29006900
H	4.85241600	-1.74548200	-1.78167200
H	5.19505400	2.15284800	1.12407300
H	5.38698700	0.90553800	2.34591000
H	6.76885900	1.90350700	1.88743300
H	-4.50269700	-3.99390800	-2.81662300
H	-2.13399400	-3.81214400	-3.46129400
H	-6.94597900	2.73533500	0.56783700
H	-7.25414100	1.66550400	-1.63771500
H	-5.29907900	0.64623700	-2.78472800
H	-3.12793300	0.57312000	-1.68663700
H	-3.20914900	-0.83678300	1.53005600
H	-6.25889100	-3.57898600	-1.21228200
H	-7.03670800	-2.63079800	0.93181100
H	-5.46030100	-1.31263600	2.32927500

Zero-point correction= 0.633380 (Hartree/Particle)
Thermal correction to Energy= 0.671196
Thermal correction to Enthalpy= 0.672140
Thermal correction to Gibbs Free Energy= 0.563789
Sum of electronic and zero-point Energies= -2034.255225
Sum of electronic and thermal Energies= -2034.217409
Sum of electronic and thermal Enthalpies= -2034.216465
Sum of electronic and thermal Free Energies= -2034.324815

Neutral (*M*)-**7H-CH** (Transition state, M06/TZVP, PCM = DMSO)

C	0.95679700	-0.45183300	-0.04569900
C	-0.15403200	-1.39795700	-0.42364500
C	-1.41980200	-0.82775500	-0.26513600
C	-1.21445600	0.47731400	0.48085800
C	0.15801900	0.59045400	0.71323700
C	-2.05530600	1.34951300	1.24491200
C	-1.50677400	1.89088800	2.43883500
C	-0.11947900	1.94405400	2.63063600
C	0.71899600	1.38932800	1.70211300
C	0.06458600	-2.74368400	-0.68903000
C	-0.99604300	-3.60087300	-0.57020300
C	-2.29336800	-3.09004600	-0.42901300
C	-2.53565000	-1.69197700	-0.50809100
C	-3.37580400	1.84802700	0.91405800
C	-4.22906300	2.32930500	1.93886400
C	-3.71320500	2.47995800	3.26153400
C	-2.38363400	2.38214500	3.45995800
C	-3.72364900	2.14928700	-0.40646300

C	-4.92116600	2.74989000	-0.71695600
C	-5.84030000	3.04485000	0.29006400
C	-5.48914200	2.84632600	1.60408300
C	-3.38974800	-3.98433500	-0.20108500
C	-4.66596600	-3.55097900	-0.20618200
C	-4.94980000	-2.23960600	-0.69541000
C	-3.87770000	-1.34306000	-0.93272300
C	-6.23810400	-1.91177700	-1.14253200
C	-6.44414100	-0.81928600	-1.95100200
C	-5.35321700	-0.04667900	-2.34918000
C	-4.10304500	-0.29704400	-1.83336200
C	1.96458000	-1.09217600	0.89400600
C	3.37264300	-0.82272900	0.74358100
C	3.84436600	-0.08542200	-0.31397000
C	2.96812300	0.40510500	-1.34867500
C	1.58008000	0.20025800	-1.27372400
C	3.48605200	1.08352300	-2.46618400
C	2.67672600	1.53439000	-3.47678800
C	1.30343200	1.31412300	-3.39574600
C	0.76852200	0.65086600	-2.29906600
C	1.52890500	-1.83259200	1.93818700
C	2.41514600	-2.38606700	2.94448400
C	3.82691900	-2.07945200	2.78239200
C	4.26455100	-1.34302300	1.74648700
O	0.54222800	1.76502300	-4.40777600
O	1.99308000	-3.06702100	3.88395000
C	5.29624900	0.20238300	-0.43406300
C	5.83490700	1.36772300	0.12106700
C	7.19827500	1.59727100	-0.03037100
C	8.01026800	0.70440000	-0.71061600
C	7.46704700	-0.44723000	-1.25575300
C	6.11131100	-0.69326700	-1.11628200
C	4.97255800	2.33258900	0.87259700
H	0.27081400	2.42701900	3.51988300
H	1.79686700	1.43794100	1.81961200
H	1.07482600	-3.11396800	-0.82294300
H	-0.85252800	-4.67588000	-0.58380900
H	-4.37874000	2.81148700	4.05138500
H	-1.93988200	2.65491100	4.41187100
H	-2.99145800	1.97013400	-1.18283400
H	-5.14764200	2.99928100	-1.74696400
H	-6.79760100	3.48792000	0.04134000
H	-6.14785500	3.16692000	2.40486800
H	-3.15409800	-5.02469100	-0.00214100
H	-5.49070200	-4.22145100	0.01073700
H	-7.05528300	-2.58712800	-0.91005500
H	-7.43522600	-0.59944600	-2.33046800
H	-5.48873800	0.75172200	-3.06938200
H	-3.25206700	0.27218600	-2.18383300
H	4.55493000	1.24870200	-2.53199400
H	3.08139700	2.05382900	-4.33661100

H	-0.30580400	0.49406700	-2.25355000
H	0.46933300	-2.03058900	2.07519200
H	4.50897700	-2.46981400	3.52966600
H	5.32272600	-1.12858600	1.64313200
H	-0.38686400	1.56111500	-4.25007800
H	7.62741900	2.49789400	0.39728500
H	9.06962400	0.90921100	-0.81395600
H	8.09414600	-1.15228800	-1.78846100
H	5.67036600	-1.59035600	-1.53983700
H	4.11717800	2.66069400	0.27418300
H	4.56701600	1.87692500	1.78175100
H	5.53762900	3.21748100	1.16658600

Zero-point correction= 0.632408 (Hartree/Particle)
Thermal correction to Energy= 0.669383
Thermal correction to Enthalpy= 0.670327
Thermal correction to Gibbs Free Energy= 0.564569
Sum of electronic and zero-point Energies= -2034.200387
Sum of electronic and thermal Energies= -2034.163412
Sum of electronic and thermal Enthalpies= -2034.162468
Sum of electronic and thermal Free Energies= -2034.268225

6. REFERENCES

[S1] Gross, B. M.; Oestreich, M. The Trityl Cation Embedded into a [7]Helicene-Like Backbone: Preparation and Application as a Lewis Acid Catalyst. *Synthesis* **2021**, *53*, 2512–2516.

[S2] Lv, M.; Yu, Y.; Sandoval-Salinas, M. E.; Xu, J.; Lei, Z.; Casanova, D.; Yang, Y.; Chen, J. Engineering the Charge-Transfer State to Facilitate Spin–Orbit Charge Transfer Intersystem Crossing in Spirobis[anthracene]diones. *Angew. Chem., Int. Ed.* **2020**, *59*, 22179-22184.

[S3] HORIBA Scientific. A Guide to Recording Fluorescence Quantum Yields; HORIBA UK Limited: Stanmore, U.K. https://www.horiba.com/uk/scientific/ (accessed 2026-04-15).

[S4] J. Schindelin, I. Arganda-Carreras, E. Frise, V. Kaynig, M. Longair, T. Pietzsch, *et al.*, Fiji: an open-source platform for biological-image analysis, Nat. Methods **2012**, *9*, 676–682.

[S5] Gaussian 16, Frisch, M. J.; Trucks, G. W.; Schlegel, H. B.; Scuseria, G. E.; Robb, M. A.; Cheeseman, J. R.; Scalmani, G.; Barone, V.; Petersson, G. A.; Nakatsuji, H.; Li, X.; Caricato, M.; Marenich, A. V.; Bloino, J.; Janesko, B. G.; Gomperts, R.; Mennucci, B.; Hratchian, H. P.; Ortiz, J. V.; Izmaylov, A. F.; Sonnenberg, J. L.; Williams-Young, D.; Ding, F.; Lipparini, F.; Egidi, F.; Goings, J.; Peng, B.; Petrone, A.; Henderson, T.; Ranasinghe, D.; Zakrzewski, V. G.; Gao, J.; Rega, N.; Zheng, G.; Liang, W.; Hada, M.; Ehara, M.; Toyota, K.; Fukuda, R.; Hasegawa, J.; Ishida, M.; Nakajima, T.; Honda, Y.; Kitao, O.; Nakai, H.; Vreven, T.; Throssell, K.; Montgomery, J. A., Jr.; Peralta, J. E.; Ogliaro, F.; Bearpark, M. J.; Heyd, J. J.; Brothers, E. N.; Kudin, K. N.; Staroverov, V. N.; Keith, T. A.; Kobayashi, R.; Normand, J.; Raghavachari, K.; Rendell, A. P.; Burant, J. C.; Iyengar, S. S.; Tomasi, J.; Cossi, M.; Millam, J. M.; Klene, M.; Adamo, C.; Cammi, R.; Ochterski, J. W.; Martin, R. L.; Morokuma, K.; Farkas, O.; Foresman, J. B.; Fox, D. J. Gaussian, Inc., Wallingford CT, 2016.
